# Supplementary material for: TReSR: A PCR-compatible DNA sequence design method for engineering proteins containing tandem repeats
Source: PLoS One. 2023 Apr 12;18(4):e0281228. doi: 10.1371/journal.pone.0281228 (PMC10096509; doi:10.1371/journal.pone.0281228)
Supplement: S1 File — (PDF) [file pone.0281228.s001.pdf]

Supplementary Information for:

TReSR: A PCR-compatible DNA sequence design method for engineering proteins containing tandem repeats

James A Davey* & Natalie K Goto*
Department of Chemistry and Biomolecular Sciences, University of Ottawa, 10 Marie-Curie, Ottawa
Ontario, K1N 6N5, Canada
* Correspondence to JAD: jamesa_davey@dfci.harvard.edu & NKG: natalie.goto@uottawa.ca

Table of Contents

Section 1. TReSR Computer Code and Documentation .... Page 2
Script 1. TReSR .... Page 3
Function 1. CodonTable........ Page 7
Function 2. CompilePaths........ Page 8
Function 3. ComputeCosine .... Page 9
Function 4. ComputePercentile........ Page 9
Function 5. CreateCodonCombinations........ Page 10
Function 6. DepthFirstSearch .... Page 10
Function 7. FilterSegment........ Page 12
Function 8. GenerateAdjacents .... Page 12
Function 9. GenerateSegmentPairs........ Page 13
Function 10. GroupAnalysis........ Page 13
Function 11. ReadAdjacents........ Page 15
Function 12. ReadPairs .... Page 15
Function 13. ReadPaths .... Page 16
Function 14. ReadSegments .... Page 16
Function 15. RecursiveCombination .... Page 17
Function 16. WriteSegmentCodons .... Page 18
Function 17. WritePaths........ Page 18
Function 18. WriteSequences........ Page 18

Section 2. TReSR Results, Thermodynamic Parameters for PCR Primers, and Synthesis of scDBD Constructs .... Page 20
S1 Table. Thermodynamic parameter thresholds and grouping analysis for segment codon combinations........ Page 20
S2 Table. Thermodynamic parameter thresholds for adjacent segment codon combinations .... Page 21
S3 Table. Calculated thermodynamic parameters for TReSR designed DNA segments .... Page 21
S1 Fig. Calculated hybridization melting temperatures for aPCR primers........ Page 22
S2 Fig. Assembly PCR and SOE synthesis workflow........ Page 23
S3 Fig. Agarose gel electrophoresis of PCR products used in synthesis of scDBD constructs .... Page 24
S4 Fig. Annotated sequences encoding single-chain tandem repeat repressor constructs. .... Page 25

Section 3. Engineering and Optimization of the *three*-Component Genetic Circuit .... Page 26
S5 Fig. Annotated plasmid sequence encoding the *three*-component genetic circuit .... Page 29
S4 Table. Data and regression analysis for the genetic circuit pDBD(eGFP)·pGFP(dGFP)·pLacI(LacI)........ Page 30
S6 Fig. Inducer series plots for the genetic circuit pDBD(eGFP)·pGFP(dGFP)·pLacI(LacI)........ Page 31
S5 Table. Data and regression analysis for the genetic circuit pDBD(eGFP)·pGFP(dGFP)·pLacI^{Q}(LacI)........ Page 32
S7 Fig. Inducer series plots for the genetic circuit pDBD(eGFP)·pGFP(dGFP)·pLacI^{Q}(LacI)........ Page 33
S6 Table. Data and regression analysis for the genetic circuit pDBD(eGFP)·pGFP(dGFP)·pLacI(LacI$_{W220F}$) .... Page 34
S8 Fig. Inducer series plots for the genetic circuit pDBD(eGFP)·pGFP(dGFP)·pLacI(LacI$_{W220F}$) .... Page 35
S7 Table. Data and regression analysis for the genetic circuit pDBD(eGFP)·pGFP(dGFP)·pLacI^{Q}(LacI$_{W220F}$)........ Page 36
S9 Fig. Inducer series plots for the genetic circuit pDBD(eGFP)·pGFP(dGFP)·pLacI^{Q}(LacI$_{W220F}$)........ Page 37
S8 Table. Inducer series non-linear regression analysis for genetic circuit engineering .... Page 38

Section 4. Expression Controls for the *three*-Component Genetic Circuit........ Page 39
S10 Fig. Expression controls for the pDBD(dGFP)·pGFP(dGFP)·pLacI(LacI$_{W220F}$) genetic circuit .... Page 41
S11 Fig. Expression controls for the pDBD(eGFP)·pGFP(dGFP)·pLacI(LacI$_{W220F}$) genetic circuit .... Page 42
S12 Fig. Expression controls for the pDBD(dGFP)·pGFP(eGFP)·pLacI(LacI$_{W220F}$) genetic circuit .... Page 43
S13 Fig. Expression controls for the pDBD(eGFP)·pGFPedGFP)·pLacI(LacI$_{W220F}$) genetic circuit .... Page 44
S9 Table. Regression analysis of expression controls for the pDBD·pGFP·pLacI(LacI$_{W220F}$) genetic circuit .... Page 45
S14 Fig. Summary of expression controls for the three-component genetic circuit........ Page 45

Section 5. Characterization of scDBD Repressor Function .... Page 46
S15 Fig. Expression plots for the pDBD(scDBD$_{IAN/IAN}$)·pGFP(eGFP)·pLacI(LacI$_{W220F}$) genetic circuit .... Page 47
S16 Fig. Expression plots for the pDBD(scDBD$_{IAN/DFT}$)·pGFP(eGFP)·pLacI(LacI$_{W220F}$) genetic circuit........ Page 48
S17 Fig. Expression plots for the pDBD(scDBD$_{DFT/IAN}$)·pGFP(eGFP)·pLacI(LacI$_{W220F}$) genetic circuit........ Page 49
S18 Fig. Expression plots for the pDBD(scDBD$_{DFT/DFT}$)·pGFP(eGFP)·pLacI(LacI$_{W220F}$) genetic circuit .... Page 50
S19 Fig. Expression plots for the pDBD(scDBD$_{IAN/IAN/\Delta CT}$)·pGFP(eGFP)·pLacI(LacI$_{W220F}$) genetic circuit .... Page 51
S20 Fig. Expression plots for the pDBD(scDBD$_{IAN/DFT/\Delta CT}$)·pGFP(eGFP)·pLacI(LacI$_{W220F}$) genetic circuit .... Page 52
S21 Fig. Expression plots for the pDBD(scDBD$_{DFT/IAN/\Delta CT}$)·pGFP(eGFP)·pLacI(LacI$_{W220F}$) genetic circuit .... Page 53
S22 Fig. Expression plots for the pDBD(scDBD$_{DFT/DFT/\Delta CT}$)·pGFP(eGFP)·pLacI(LacI$_{W220F}$) genetic circuit........ Page 54
S10 Table. Regression analysis of expression plots for characterization of scDBD repressor function .... Page 55

Section 6. References for Supplementary Information........ Page 56

Section 1. TReSR Computer Code and Documentation

TReSR Instructions. The TReSR package included with this manuscript contains the executable script TReSR.py, input files that were used to make the scDBD sequence shown in Fig 2, and output files generated by the calculation. All scripts were written for and executed using the Python 3.10 programming language (https://www.python.org/downloads/). The TReSR algorithm was executed in a series of five steps as indicted in the comments included with the TReSR.py script (Script 1). Comments (red), built-in Python functions (purple), functions coded for TReSR (blue), characters and strings (green), and built-in expressions (orange) are annotated by colour for ease of interpretation. To generate a redesigned TR-encoding DNA sequence for a different target protein, the TReSR.py script must be modified as specified below, and the protocol executed according these instructions:

Step 1. Specify the directory location (Line 6) and encode the *single*-letter amino acid sequence for your TR protein (1 copy) separated into segments 5 – 7 residues in length (Line 9). Ensure that each segment has a unique identification character or string (*e.g.,* 'A', 'B', 'C',…) specified in Line 8. The comma-delimited file 'segments.csv' will be created listing the set of all DNA sequence codon combinations encoding the amino acid sequence for each segment when TReSR is executed (Line 15).

Step 2.1. For each sequence in 'segments.csv', calculate melting temperature (Tm) for thermodynamic parameters using the UNAFold webserver (http://www.unafold.org/Dinamelt/applications/two-state-melting-hybridization.php) *two*-state melting hybridization application for: homodimerization of forward sequence (T_{FF}), homodimerization of the reverse complement sequence (T_{RR}), hybridization of the forward and reverse complement sequences (T_{FR}), and if needed, hybridization of the forward sequence with the reverse complement belonging to the WT sequence (T_{WT}). If consideration of the wild-type sequence is to be excluded from thermodynamic parameter analysis, then enter a T_{WT} value of 0.0 for all sequence entries. Add Tm values to the file 'segments.csv' and save as 'evaluated_segments.csv' ensuring that the top row includes the labels defining each thermodynamic parameter: T(FF), T(RR), T(FR), and T(WT).

Step 2.2. Reinitialize execution of TReSR to implement the percentile-based (Line 26) pruning of segment sequences and write the retained sequences to the file 'filtered_segments.csv'. The fraction of filtered sequences can be altered by adjusting the percentile rank passed to the function ComputePercentile (Line 26) or the thermodynamic parameter thresholds passed directly to the function FilterSegment (Line 31).

Step 3. Conduct a pair-wise comparison of sequence identity for each segment's set of filtered sequences and write the results to the file 'paired_segments.csv'. The results of this pair-wise comparison are then read back into TReSR to perform a grouping analysis assigning a group identification index number for each sequence (in square parentheses following the segment identifier assigned in Step 1) whose results are then written to the file 'grouped_segments.csv'. Grouping analysis identifies segment-specific sets of similar (same group index number) and dissimilar (different group index number) sequences by comparing the identity between sequence pairs in addition to the cosine similarity of their identities with respect to every other sequence in the segment. The degree of cluster aggregation can be adjusted by altering the parameter thresholds set in the GroupAnalysis function (Line 57).

Step 4.1. To reduce the size of the computational problem, the total number of filtered and grouped sequences listed in 'grouped_segments.csv' can be reduced by pruning the number of sequence groupings considered by TReSR (performed external to and independent of Script 1). For the purposes of this manuscript, TReSR was restricted to sequences belonging to four randomly selected groups for each segment, written to the file 'select_grouped_segments.csv'.

Step 4.2. Modify the list of paired adjacent segments in Line 71 in TReSR.py so that it specifies adjacent paris of segments for your sequence following the format provided in the script (originally applied to the DNA sequence encoding the 89-residue TR gene from the 11 segments that had been defined in Step 1). Resume execution of TReSR to create a list of adjacent segment sequence pairs from the set of select grouped and filtered sequences compiled in the file 'select_grouped_segments.csv'. The list of adjacent segment sequence pairs is written to the file 'select_adjacent_segments.csv'.

Step 4.3. For each sequence listed in 'select_adjacent_segments.csv' calculate the Tm values for T_{FF}, T_{RR}, and T_{FR} thermodynamic parameters using the UNAFold webserver as was done previously in Step 2.1. Add these Tm values to the file 'select_adjacent_segments.csv' along with their respective labels (*e.g.,* 'T(FF)', 'T(RR)', and 'T(FR)') to the top row and save the file as 'evaluated_adjacent_grouped_segments.csv'.

Step 4.4. Reinitialize execution of TReSR to implement the pruning of the adjacent segment sequence pairs listed in the file 'evaluated_adjacent_grouped_segments.csv' using percentile-based thresholds for T_{FF} and T_{RR} (Line 86) and a fixed Tm value of 80.0 °C for T_{FR} (Line 92) thermodynamic parameters. The fraction of filtered sequences can be altered by adjusting the percentile rank passed to the function ComputePercentile (Line 86) or the thermodynamic parameter thresholds passed directly to the function FilterSegment (Line 91). The list of filtered adjacent segment sequence pairs is then written to the file 'filtered_adjacent_grouped_segments.csv'.

Step 5.1. Generate a set of segment sequences suitable for encoding the TR gene by a depth-first-search (Line 110) using the path specified on Line 103 describing the order of corresponding segments and groups used to reassemble the full-length DNA sequence by specifying the set of adjacent segments pairs using the segment designation and group assignment (*i.e.,* ['A[1]', 'B[2]'] to employ codon combinations belonging to segment A, group 1 and segment B, group 2) as a nest list. The list of segment paths solved by depth-first-search is then written to the file 'segment_paths.csv'. The DepthFirstSearch function includes settings (Line 111) specifying the target number of paths to assemble (goalCount) as well as the permitted number of unsuccessful attempts (failCount) which can be adjusted according to the difficulty of the computational problem.

Step 5.2. The TReSR algorithm is terminated with a final step compiling the set of segmented paths specified in 'segment_paths.csv' into assembled DNA sequences encoding the TR gene which are then written to the file 'sequence_paths.csv'. The sequence output is organized according to the cassette (set of tandem repeat duplications specified as a dictionary, Line 120) and fragment (assembled set of contiguous segments specified as a dictionary, Line 122) formatting passed to the function CompilePaths (Line 130). This formatting assigns a tandem repeat identifier (*e.g.,* 'N' or 'C' representing N-terminal and C-terminal domain duplications comprised of fragments 2 and 4, and 5 and 7, respectively) to the cassette dictionary (Line 120). The formatting also requires that the list of segments belonging to each fragment be specified in the fragments dictionary (Line 122) using the fragment nomenclature employed in the cassette dictionary (Line 120).

Script 1. TReSR

```
 1  # TReSR Script
 2  from math import sqrt
 3  from random import randrange
 4  # Step 1| Generate all DNA sequence codon combinations for protein segments
 5  print('START')
 6  directory = ''
 7  codons = CodonTable(directory + 'codons.txt', delim=' ')
 8  segments = ['A', 'B', 'C', 'D', 'E', 'F', 'G', 'H', 'I', 'J', 'K']
 9  proteins = ['MKPVTL', 'YDVAE', 'YAGVS', 'DFTVST', 'VVNQASH', 'QSLLI', 'GVATS',\
10              'SLALH', 'APSQI', 'VAAIK', 'SRADQ']
11  sequences = []
12  for segment, protein in zip(segments, proteins):
13      sequences = sequences + CreateCodonCombinations(segment, protein, codons,\
14                                                      idStart='(', idEnd=')')
15  WriteSegmentCodons(directory + 'segments.csv', sequences,\
16                     'Segment(Codons),FWD,REV,FRQ')
17  # Step 2.2| Evaluate the thermodynamic properties (Tm for FF, RR, FR, and WT) for
18  # Step 2.2| all codon combinations and write to .csv file, then filter codon
19  # Step 2.2| combinations for each segment and write to .csv file
20  sequences = ReadSegments(directory + 'evaluated_segments.csv', delim='(',\
21                           name='Segment(Codons)', fwd='FWD', rev='REV',\
22                           FRQ='FRQ', T_FF='T(FF)', T_RR='T(RR)', T_FR='T(FR)',\
23                           T_WT='T(WT)')
24  filterSequences = []
25  for segment in segments:
26      parameters = ComputePercentile(sequences[segment],\
27                                     prcs=[0.5, 0.5, 0.1, 0.5],\
28                                     reverse=[True, True, False, True],\
29                                     cols=[4, 5, 6, 7],\
30                                     headers=['T_FF', 'T_RR', 'T_FR', 'T_WT'])
31      filterSequences = filterSequences + FilterSegment(sequences[segment],\
```

```
                                                      T_FF=parameters['T_FF'],\
                                                      T_RR=parameters['T_RR'],\
                                                      T_FR=parameters['T_FR'],\
                                                      T_WT=parameters['T_WT'],\
                                                      ADJ=False, idxT_FF=4,\
                                                      idxT_RR=5, idxT_FR=6,\
                                                      idxT_WT=7, delim=',')
WriteSegmentCodons(directory + 'filtered_segments.csv', filterSequences,\
                   'Segment(Codons),FWD,REV,FRQ,T(FF),T(RR),T(FR),T(WT)')
# Step 3| Generate all DNA sequence codon combination pairs for each protein
# Step 3| segment and evaluate their percent sequence identity, then assign
# Step 3| group identities according to their comparative percent identities
sequences = ReadSegments(directory + 'filtered_segments.csv', delim='(',\
                         name='Segment(Codons)', fwd='FWD', rev='REV',\
                         FRQ='FRQ', T_FF='T(FF)', T_RR='T(RR)', T_FR='T(FR)',\
                         T_WT='T(WT)')
pairs = []
for segment in segments:
    pairs = pairs + GenerateSegmentPairs(sequences[segment])
WriteSegmentCodons(directory + 'paired_segments.csv', pairs,\
                   'iSegment,jSegment,iFWD,iREV,jFWD,jREV,BP(n/N)')
pairs = ReadPairs(directory + 'paired_segments.csv', iName='iSegment',\
                  jName='jSegment', BP='BP(n/N)', delim='(')
groups = []
for segment in segments:
    group = GroupAnalysis(segment, sequences[segment], pairs[segment],\
                          iCmp=0, jCmp=1, ijBP=2, grpDelim='+',\
                          groupStart='[', groupEnd=']', codonDelim='(',\
                          segName=0, cos=0.9975, prct=0.8)
    groups = groups + group
WriteSegmentCodons(directory + 'grouped_segments.csv', groups,\
                   'Segment[Group](Codons),FWD,REV,FRQ,T(FF),T(RR),T(FR),T(WT)')
# Step 4.2| DNA sequence codon combinations belonging to four randomly selected
# Step 4.2| groups from each protein segment are used to generate pairs of
# Step 4.2| segment adjacent codon combinations
sequences = ReadSegments(directory + 'select_grouped_segments.csv', delim='[',\
                         name='Segment[Group](Codons)', fwd='FWD', rev='REV',\
                         FRQ='FRQ', T_FF='T(FF)', T_RR='T(RR)', T_FR='T(FR)',\
                         T_WT='T(WT)')
adjacentMap = [['A', 'B'], ['B', 'C'], ['C', 'D'], ['D', 'E'], ['F', 'G'],\
               ['G', 'H'], ['H', 'I'], ['I', 'J'], ['J', 'K'], ['K', 'A']]
adjacents = GenerateAdjacents(sequences, adjacentMap, idxName=0, idxFwd=1,\
                              idxRev=2, adjDelim='+', strDelim=',')
WriteSegmentCodons(directory + 'select_adjacent_segments.csv', adjacents,\
                   'Segment[Group](Codons),FWD,REV')
# Step 4.4| Thermodynamic properties of segment adjacent codon combinations were
# Step 4.4| evaluated (Tm for FF, RR, and FR) and filtered, writing the remaining
# Step 4.4| adjacent DNA sequence codon combinations to a .csv file
sequences = ReadAdjacents(directory + 'evaluated_adjacent_grouped_segments.csv',\
                          name='Segment[Group](Codons)', fwd='FWD', rev='REV',\
                          T_FF='T(FF)', T_RR='T(RR)', T_FR='T(FR)', adj='+',\
                          grp='[')
parameters = dict()
for key in sequences:
    parameters[key] = ComputePercentile(sequences[key], prcs=[0.200, 0.200],\
                                        reverse=[True, True], cols=[3, 4],\
                                        headers=['T_FF', 'T_RR'])
output = []
for key in sequences:
    filtered = FilterSegment(sequences[key], T_FF=parameters[key]['T_FF'],\
                             T_RR=parameters[key]['T_RR'], T_FR=80.0, T_WT=0.0,\
                             ADJ=True, idxT_FF=3, idxT_RR=4, idxT_FR=5)
    output = output + filtered
```

```
 95 WriteSegmentCodons(directory + 'filtered_adjacent_grouped_segments.csv', output,\
 96                      'Segment[Group](Codons),FWD,REV,T(FF),T(RR),T(FR)')
 97 # Step 5.1| Depth-first-search of paths visiting select segment groupings to
 98 # Step 5.1| reconstruct the full-length tandem repeat DNA sequence
 99 sequences = ReadAdjacents(directory + 'filtered_adjacent_grouped_segments.csv',\
100                           name='Segment[Group](Codons)', fwd='FWD', rev='REV',\
101                           T_FF='T(FF)', T_RR='T(RR)', T_FR='T(FR)', adj='+',\
102                           grp='[')
103 groupPath = [['A[6]', 'B[6]'], ['B[6]', 'C[16]'], ['C[16]', 'D[1]'],\
104              ['D[1]', 'E[7]'], ['F[23]', 'G[46]'], ['G[46]', 'H[45]'],\
105              ['H[45]', 'I[16]'], ['I[16]', 'J[4]'], ['J[4]', 'K[6]'],\
106              ['K[6]', 'A[4]'], ['A[4]', 'B[1]'], ['B[1]', 'C[13]'],\
107              ['C[13]', 'D[15]'], ['D[15]', 'E[5]'], ['F[76]', 'G[76]'],\
108              ['G[76]', 'H[8]'], ['H[8]', 'I[3]'], ['I[3]', 'J[15]'],\
109              ['J[15]', 'K[15]']]
110 paths = DepthFirstSearch(sequences, groupPath, paths=[], rejects=[],\
111                          goals=0, goalCount=100, fails=0, failCount=1000,\
112                          groupDelim='[', codonDelim='(', adjDelim='+')
113 WritePaths(directory + 'segment_paths.csv', paths, delimPair='+', delimGroup='[')
114 # Step 5.2| Write the full length sequence for each reconstructed path
115 paths = ReadPaths(directory + 'segment_paths.csv', ['N', 'C'])
116 segments = ReadSegments(directory + 'select_grouped_segments.csv', delim='[',\
117                         name='Segment[Group](Codons)', fwd='FWD', rev='REV',\
118                         FRQ='FRQ', T_FF='T(FF)', T_RR='T(RR)', T_FR='T(FR)',\
119                         T_WT='T(WT)')
120 cassettes = {'Fragment.2': 'N', 'Fragment.4': 'N',\
121              'Fragment.5': 'C', 'Fragment.7': 'C'}
122 fragments = {'Fragment.2': ['Segment.A', 'Segment.B', 'Segment.C',\
123                             'Segment.D', 'Segment.E'],
124              'Fragment.4': ['Segment.F', 'Segment.G', 'Segment.H',\
125                             'Segment.I', 'Segment.J', 'Segment.K'],
126              'Fragment.5': ['Segment.A', 'Segment.B', 'Segment.C',\
127                             'Segment.D', 'Segment.E'],
128              'Fragment.7': ['Segment.F', 'Segment.G', 'Segment.H',\
129                             'Segment.I', 'Segment.J', 'Segment.K']}
130 sequences = CompilePaths(paths, segments, cassettes, fragments)
131 WriteSequences(directory + 'sequence_paths.csv', sequences)
132 print('END')
```

TReSR Technical Description. The TReSR algorithm is executed from a python script (Script 1) over a series of *five* steps. The TReSR algorithm employs dictionary objects and a functional programming architecture to read, execute, and save data producing comma delimited file format outputs (*i.e.,* .csv files). The TReSR algorithm employs 18 functions whose programming and technical descriptions are included and described in the section (TReSR Functions) following this technical description of the TReSR script.

The **first step** (Lines 6 through 16) involves the generation of all codon combinations (*sequences*, executed by calling the function CreateCodonCombinations, Line 13) encoding a list of amino acids (*protein*, Line 9) belonging to a series of protein segments (*segments*, Line 8). These codon combinations are built using definitions provided via an input file (*codons*, the space delimited input file 'codons.txt' is read into a dictionary using the function CodonTable, Line 7). Each codon combination is assigned a designation which includes their segment name followed by an identification code (an integer, 1 ≤ index ≤ 6) identifying the index of the selected silent mutations from *codons* enclosed in curved parentheses. For example, the designation A(1.2.3.4.1.6) indicates that the codon combination for segment A, encoding the protein sequence MKPVTL, is constructed from the 1st, 2nd, 3rd, 4th, 1st, and 6th codons listed in the *codons* dictionary. The codon combinations are then written to the file 'segments.csv' via the function WriteSegmentCodons (Line 15).

Initiation of the **second step** (Lines 20 through 40) first requires that the external-to-program calculation of thermodynamic parameters be conducted providing melting temperature (Tm) values for homodimerization and hybridization of DNA sequences listed in 'segments.csv', including: 1. forward and forward (T_{FF}), 2. reverse complement and reverse complement (T_{RR}), 3. forward and reverse complement (T_{FR}), and 4. forward and wild-type reverse complement (T_{WT}). For the purposes of this study, Tm values were calculated

using the DINAMelt two-state melting hybridization application made available through the UNAFold Web Server. These thermodynamic parameters are incorporated into the 'segments.csv' file, amending the first row to include the identity of each parameter: T(FF), T(RR), T(FR), and T(WT), and saved as 'evaluated_segments.csv'. This file is then read back into TReSR (*sequences*, executed by calling the function ReadSegments, Line 20). A set of thresholds for each thermodynamic parameter are solved by computing percentiles (T_{FF} = 0.5, T_{RR} = 0.5, T_{FR} = 0.1, and T_{WT} = 0.5) for each segment (*parameters*, using the function ComputePercentile, Line 26). These thermodynamic parameter thresholds are then used to prune codon combination sequences (*filterSequences*, using the function FilterSegment, Line 31) which are then written to the file 'filtered_segments.csv' using the function WriteSegmentCodons (Line 39).

The **third step** of the TReSR algorithm (Lines 44 through 63) generates pairs of codon combinations (*pairs*, by calling the function GenerateSegmentsPairs, Line 50) restricted to filtered sequences belonging to the same segment listed in and read into TReSR (*sequences*, using the function call ReadSegments, Line 44) from the file 'filtered_segments.csv'. The function GenerateSegmentsPairs (Line 50) performs a comparison of sequence identities between the codon combination pairs (reported as a fraction) and the results are written to the file 'paired_segments.csv' using the function WriteSegmentCodons (Line 51). This output is then read back into the program (*pairs*, using the function call ReadPairs, Line 53) for grouping analysis. Grouping analysis utilizes the percent sequence identities to define the edges of a network graph consisting of vertices representing individual codon combinations. This graph is used to group codon combinations belonging to the same segment according to their pair-wise similarity (*groups*, using the function call GroupAnalysis, Line 57). Codon combinations are considered to be similar (*i.e.*, belong to the same group) if: 1. they share at least 0.80 sequence identity, and 2. they share a highly similar profile of percent sequence identities with the remaining codon combinations in the segment, as determined by cosine similarity (threshold set to 0.9975, function ComputeCosine is called within the function GroupAnalysis). Codon combination designations are then amended to include their group assignment indicated as an integer enclosed in square parentheses. For example, the codon combination A[2](1.2.3.4.1.6) belongs to the 2nd grouping of codon combinations for segment A. This grouping analysis is then written to the file 'grouped_segments.csv' executed by calling the function WriteSegmentCodons (Line 62). The incorporation of group assignment in this manner allows for the rapid recall and identification of dissimilar codon combinations which encode for the same amino acid sequence.

The **fourth step** of the TReSR algorithm is conducted in four parts (Lines 67 through 76 and Lines 80 through 96, for Steps 4.2 and 4.4, respectively) starting with Step 4.1 involving the reduction of the total sequence space considered in the calculation by randomly selecting sequences from four groups from each segment, written to the file 'select_grouped_segments.csv'. Step 4.2 reads this reduced sequence space into TReSR (*sequences*, using the function call ReadSegments, Line 67) to compile a list of sequences assembled from pairs of adjacent segments (*adjacents*, using the function GenerateAdjacents, Line 73) for a set of user specified segment pairs (*adjacentMap*, Line 71) which defines the order and assembly of the redesigned TR DNA sequence. This set of adjacent sequences are then written to the file 'select_adjacent_segments.csv' using the function WriteSegmentCodons (Line 75). Step 4.3 involves the external-to-program evaluation of thermodynamic parameters (T_{FF}, T_{RR}, and T_{FR}) for sequences listed in 'select_adjacent_segments.csv' using the UNAFold Web Server (as was performed in Step 2). The list of adjacent codon combinations and their thermodynamic parameters is then saved to the file 'evaluated_adjacent_grouped_segments.csv' and read back into the program (*sequences*, executed by calling the function ReadAdjacents, Line 80) to initiate Step 4.4. This list is then pruned (*filtered*, implemented by calling the function FilterSegment, Line 91) employing thermodynamic parameter thresholds computed from percentiles for each adjacent segment pair (*parameters*, percentiles set at T_{FF} = 0.2 and T_{RR} = 0.2 using the function call ComputePercentiles, Line 86) or set at a fixed value (*parameters*, T_{FR} = 80.0 °C). This pruned list of grouped sequences from adjacent segment pairs is then written to the file 'filtered_adjacent_grouped_segments.csv' using the function WriteSegmentCodons (Line 95).

The **fifth** and final **step** of the TReSR algorithm (Lines 99 through 131) solves for adjacent segment sequence paths that assemble the full-length DNA sequence encoding the TR gene using the set of pruned and grouped adjacent sequences pairs listed in the file 'filtered_adjacent_grouped_segments.csv' (*sequences*, read into TReSR by calling the function ReadAdjacents, Line 99). Solution of adjacent segment sequence paths is guided by a user-specified list of segments that includes specific and unique grouping designations (*groupPath*, Line 103). This path search is conducted using a depth-first-search algorithm (*paths*, executed by calling the function DepthFirstSearch, Lines 110) to generate a list of segments

representing DNA sequences that encode for duplicated amino acid sequences. The result is a list of paths assembled from dissimilar codon combinations (*i.e.,* sequences belonging to unique segment groupings) that are then written to the output file 'segment_paths.csv' using the function WritePaths (Line 113). The list of segment paths is then read back into TReSR using the function ReadPaths (Line 115) and transcribed into DNA sequences (*sequences*, executed by calling the function CompilePaths, Line 130) serving as candidate DNA templates for construction of the TR gene. DNA sequences are then written to the file 'sequence_paths.csv' using the function WriteSequences (Line 131) having output organized as a list of fragments (*e.g.,* 'Fragment.2', 'Fragment.4', 'Fragment.5', and 'Fragment.7') conforming to user specified definitions describing the identity (*e.g.,* 'N' or 'C' for N-terminal or C-terminal domain, respectively) of duplicated cassettes (*cassettes*, Line 120) matching the names of their constituent segments (*fragments*, Line 122) as written in the file 'segments_paths.csv'.

TReSR Calculation Considerations. The complexity of design problems solved using the TReSR protocol is determined by *multiple* factors, including: the number of amino acid residues encoded for each protein segment, the number of codon combination pairs to be evaluated, the number of adjacent segments used to assemble the gene sequence, and the efficiency of thresholds employed to eliminate non-productive codon combinations and adjacent segment sequence pairs. While it may initially appear advantageous to design TR DNA sequences by simply considering segments encompassing full-length domain sequences, the number of codon combinations constituting increasingly larger amino acid sequences, calculated as the product for the number of all degenerate codons encoding each residue in the segment, is computationally prohibitive (*e.g.*, there are 1,024 and 1,048,576 unique DNA sequence combinations built from the *four* degenerate codons encoding poly-alanine segments 5 and 10 residues in length, respectively). The size of this problem is further compounded by the task of evaluating pairs of codon combinations prior to grouping analysis, with the number of compared pairs computed as: $n \times (n - 1) \div 2$, where n is the number of codon combinations (*e.g.*, there are 523,776 and 549,755,289,600 paired sequences generated from unique codon combinations encoding poly-alanine segments 5 and 10 residues in length, respectively). To simplify the scope of the design problem, thresholds for calculated thermodynamic parameters (T_{FF}, T_{RR}, T_{FR}, and T_{WT}) were applied to filtered codon combinations based on their suitability for PCR. Importantly, while application of stringent thresholds will reduce the computational bottleneck associated with evaluating and grouping pairs of codon combinations, the application of severely selective thresholds may reduce the diversity of codon combinations impacting the feasibility of identifying unique DNA sequence groupings. To circumvent this difficulty, we chose to implement percentile-based filtering thresholds which ensured selection of PCR compatible primers, while simultaneously restricting the adjacent segment path search space by randomly selecting *four* groups of unique codon combinations. Because it is difficult to anticipate the parameters and scope of the codon design space for future applications, the computer code and documentation for the TReSR protocol has been included allowing users to independently select which parameters and protocols best suit their calculation needs.

TReSR Functions. This section provides computer code and technical descriptions for the 18 programmed functions employed by the executable script TReSR.py. Function names (blue), input arguments and keywords (red), output variables (**red**), built-in Python functions (purple), characters and strings (green), and built-in expressions (orange) are annotated by colour for ease of interpretation.

Function 1. CodonTable

```
def CodonTable(inputFile, delim=' '):
    table = open(inputFile, 'r')
    codons = dict()
    for data in table:
        data = data.replace('\n', '')
        aa, no, fwd, rev, frq = data.split(delim)
        if all([codon != aa for codon in codons]):
            codons[aa] = dict()
        codons[aa][no] = {'FWD': fwd, 'REV': rev, 'FRQ': frq}
    table.close()
    return(codons)
```

Function 1. Technical description: The function CodonTable reads a user-generated input text file (with location and file name specified via the argument inputFile) containing codon information used to generate

codon combinations for all protein segments. The input text file (inputFile) is organized with a unique codon on each line, with in-line information separated by a delimiter (specified via the keyword delim), organized in columns with the following order: the amino acid character code (aa, *single*-character or *multiple*-character string), the codon index number (no, an integer: 1 ≤ no ≤ number of degenerate codons), the codon's forward DNA sequence (fwd, *three*-characters {'A', 'C', 'G', 'T'} organized 5′ to 3′ in the forward sequence direction), the codon's reverse complement DNA sequence (rev, *three*-characters {'A', 'C', 'G', 'T'} organized 5′ to 3′ in the reverse complement direction), and the codon's frequency (frq, a floating point number: 0 ≤ frq ≤ 1). The information is compiled into a set of nested dictionary objects (Line 9, **codons**) such that each codon is referenced using *three* dictionary keys, indicating: its amino acid character code (aa), its codon number (no), and followed by the dictionary keys 'FWD', 'REV', or 'FRQ' to retrieve the codon's forward sequence (fwd), reverse compliment sequence (rev), or codon frequency (frq), respectively. The function returns this compiled set of nested dictionary objects (**codons**).

Function 2. CompilePaths

```
1  def CompilePaths(paths, segments, cassettes, fragments,\
2                   segIndex=0, seqIndex=1, rmv=[';'], groupDelim='['):
3      seqdict = dict()
4      for path in paths:
5          seqdict[path] = dict()
6          for frg in fragments:
7              ss = ''
8              for seg in fragments[frg]:
9                  idSeg = paths[path][seg][cassettes[frg]]
10                 nameSeg = idSeg[0:idSeg.find(groupDelim)]
11                 search = True
12                 idx = 0
13                 while search:
14                     idx = idx + 1
15                     name = segments[nameSeg][idx][segIndex]
16                     search = False if name == idSeg or\
17                              idx + 1 >= len(segments[nameSeg]) else True
18                 seq = segments[nameSeg][idx][seqIndex]
19                 for it in rmv:
20                     seq = seq.replace(it, '')
21                 ss = ss + seq
22             seqdict[path][frg] = ss
23     return(seqdict)
```

Function 2. Technical description: The function CompilePaths transcribes and returns sequences in a nested dictionary (**seqdict**, keyed using the path index string and fragment name) from a set of segments (paths, passed to the function as a dictionary and keyed using the path index integer) for segment paths which assemble to produce the full-length DNA template encoding the TR gene computed by the TReSR algorithm. CompilePaths utilizes a dictionary of segment sequences passed to the function as an argument (segments, keyed using the segment name delivering a nested list of sequences for that segment). The function keywords segIndex and seqIndex identify the index of list elements corresponding to the sequence identification (*e.g.,* A[2](1.2.3.4.1.6), representing the sequence encoding segment A constructed from the set of 1st, 2nd, 3rd, 4th, 1st, and 6th codon combinations) and forward DNA sequence for each list element in the dictionary segments, respectively. CompilePaths operates by iteratively (Line 4) extracting the segment names (*nameSeg*, Lines 9 and 10) from each path in paths. These segment names are iteratively (Line 6) matched (Lines 13 through 17) to the segment names in fragments (a dictionary passed to this function as an argument), organized by the dictionary cassettes (a dictionary passed to this function as an argument). Upon identification of a matched segment, CompilePaths then appends the forward sequence (*seq*, Line 18) to the string of sequences (*ss*, Line 21) encoding the current fragment (*frg*, Line 6) following the removal (Lines 19 and 20) of any characters belonging to the list rmv (a function keyword). The completed sequence for each fragment is then written to the output nested dictionary **seqdict** (keyed using the path and fragment names, Line 22) and returned by the function.

Function 3. ComputeCosine

```
1  def ComputeCosine(iVec, jVec):
2      ijCos = 0.0
3      iiCos = 0.0
4      jjCos = 0.0
5      for i, j in zip(iVec, jVec):
6          ijCos = ijCos + (i * j)
7          iiCos = iiCos + (pow(i, 2))
8          jjCos = jjCos + (pow(j, 2))
9      iiCos = sqrt(iiCos)
10     jjCos = sqrt(jjCos)
11     ijCos = ijCos / (iiCos * jjCos)
12     return(ijCos)
```

Function 3. Technical description: The function ComputeCosine calculates and returns the cosine similarity (**ijCos**, a floating point number: −1.0 ≤ ijCos ≤ +1.0) between *two*-equal length (paired) input vectors (iVec and jVec, each passed to the function as arguments organized as a list of floating point numbers). The calculation iteratively (Lines 5 through 8) evaluates the dot product between the input vectors (Line 6) and their respective magnitudes (iVec: Lines 7 and 9, jVec: Lines 8 and 10). The cosine similarity (**ijCos**) is then calculated (Line 11) by normalizing the dot product by the product of each vector's magnitude: $\cos\theta = \frac{\mathrm{iVec}\cdot\mathrm{jVec}}{\|\mathrm{iVec}\|\cdot\|\mathrm{jVec}\|} = \frac{\sum \mathrm{iVec}\cdot\mathrm{jVec}}{\sqrt{\sum \mathrm{iVec}^2}\cdot\sqrt{\sum \mathrm{jVec}^2}}$. Two identical vectors are reported as having a cosine similarity value of one ($\cos\theta = 1$), while two completely dissimilar (orthogonal) vectors have a cosine similarity value of zero ($\cos\theta = 0$), and two completely opposite vectors have a cosine similarity value of negative one ($\cos\theta = -1$).

Function 4. ComputePercentile

```
1  def ComputePercentile(codons, prcs=[], reverse=[], cols=[], headers=[]):
2      values = dict()
3      idxPrc = len(codons)
4      thresholds = dict()
5      for col in cols:
6          values[str(col)] = []
7      for codon in codons:
8          for key, col in zip(values, cols):
9              values[key] = values[key] + [float(codon[col])]
10     for key, cmd, prc, header in zip(cols, reverse, prcs, headers):
11         key = str(key)
12         data = values[key]
13         data = sorted(data, reverse=cmd)
14         it = int(round(idxPrc * prc, 0)) - 1
15         thresholds[header] = data[it]
16     return(thresholds)
```

Function 4. Technical description: The function ComputePercentile calculates and returns a dictionary of threshold values (**thresholds**, Lines 14 and 15) for a corresponding list of user-specified percentiles (prcs, set as list of floating point numbers passed to the function as a keyword) across data sets belonging to a dictionary of codon combinations (codons, passed to the function as an argument). Each dictionary entry in codons is keyed by the codon combination designation (*i.e.,* a string of characters indicating the segment name in square parentheses followed by the codon combination in curved parentheses) that includes a list of parameters, whose identities (headers, list of strings) and list element indices (cols, list of integers) are specified via function keywords. The function compiles a dictionary of parameter lists (*values*, Lines 7 through 9) and sorts (*sorted*, Lines 10 through 13) these lists from least to greatest (reverse is False) or greatest to least (reverse is True) based on a list of Boolean elements (reverse, passed to the function as a keyword) having the same organization as the function keywords headers, cols, and prcs.

Function 5. CreateCodonCombinations

```python
1  def CreateCodonCombinations(segment, sequence, codons, idStart='(', idEnd=')'):
2      sequence = [seq for seq in sequence]
3      elements = [len(codons[seq]) for seq in sequence]
4      combinations = RecursiveCombination(elements, vector=[[]], iteration=0)
5      output = []
6      for combination in combinations:
7          sfwd = ''
8          srev = ''
9          sfrq = 0.0
10         name = segment + idStart
11         for it, aa in zip(combination, sequence):
12             name = name + it + '.'
13             sfwd = sfwd + codons[aa][it]['FWD']
14             sfrq = sfrq + float(codons[aa][it]['FRQ'])
15         for it, aa in zip(combination[::-1], sequence[::-1]):
16             srev = srev + codons[aa][it]['REV']
17         sfrq = sfrq / len(combination)
18         output.append(name[:-1] + idEnd + ',' + sfwd + ';,' + srev + ';,' +\
19                       str(sfrq))
20     return(output)
```

Function 5. Technical description: The function CreateCodonCombinations generates all codon combinations (codons, a dictionary of DNA codons passed to this function as an argument) for a user specified amino acid sequence (sequence, a string composed of characters matching keys belonging to the codons dictionary). The function names each codon combination beginning with the segment designation (segment, an identifying character or string unique to the segment and passed to this function as an argument) and includes the integer index of the codons used to assemble the sequence, flanked by the characters idStart and idEnd (set as function keywords). The function returns a comma-delimited list of codon combinations (**output**) which includes the name, forward DNA sequence (terminated with a semi-colon), reverse complement DNA sequence (terminated with a semi-colon), and average codon frequency for the assembled sequence (Lines 18 and 19).

Function 6. DepthFirstSearch

```python
def DepthFirstSearch(adjacents, groupPath, paths=[], rejects=[],\
                     goals=0, goalCount=100, fails=0, failCount=1000,\
                     groupDelim='[', codonDelim='(', adjDelim='+'):
    path = ''
    for iPair, jPair in groupPath:
        iSeg = iPair[:iPair.index(groupDelim)]
        jSeg = jPair[:jPair.index(groupDelim)]
        ijSeg = iSeg + adjDelim + jSeg
        curAdj = []
        if path != '':
            prvSeg = path.split(adjDelim)[-1]
            if prvSeg[:prvSeg.index(groupDelim)] == iSeg:
                for it in adjacents[ijSeg]:
                    iGroup, prvJ = it[0].split(adjDelim)
                    jGroup = prvJ[:prvJ.index(codonDelim)]
                    if prvSeg == iGroup and jGroup == jPair and\
                       all([prvJ != test for test in rejects]):
                        curAdj.append(it[0].split(adjDelim)[-1])
            else:
                for it in adjacents[ijSeg]:
                    iGroup, jGroup = it[0].split(adjDelim)
                    iGroup = iGroup[:iGroup.index(codonDelim)]
                    jGroup = jGroup[:jGroup.index(codonDelim)]
```

```
24                     if iGroup == iPair and jGroup == jPair:
25                         curAdj.append(it[0])
26         else:
27             for it in adjacents[ijSeg]:
28                 iGroup, jGroup = it[0].split(adjDelim)
29                 iGroup = iGroup[:iGroup.index(codonDelim)]
30                 jGroup = jGroup[:jGroup.index(codonDelim)]
31                 if iGroup == iPair and jGroup == jPair:
32                     curAdj.append(it[0])
33         if curAdj == []:
34             fails = fails + 1
35             if path != '':
36                 rejects.append(path.split(adjDelim)[-1])
37             if fails >= failCount:
38                 return(paths)
39             else:
40                 return(DepthFirstSearch(adjacents, groupPath, paths=paths,\
41                                         rejects=rejects, goals=goals,\
42                                         goalCount=goalCount, fails=fails,\
43                                         failCount=failCount,\
44                                         groupDelim=groupDelim,\
45                                         codonDelim=codonDelim,\
46                                         adjDelim=adjDelim))
47         else:
48             path = path + adjDelim + curAdj[randrange(0, len(curAdj))]
49     goals = goals + 1
50     paths.append(path[1:])
51     if goals >= goalCount:
52         return(paths)
53     else:
54         return(DepthFirstSearch(adjacents, groupPath, paths=paths,\
55                                 rejects=rejects, goals=goals,\
56                                 goalCount=goalCount, fails=fails,\
57                                 failCount=failCount, groupDelim=groupDelim,\
58                                 codonDelim=codonDelim,\
59                                 adjDelim=adjDelim))
```

Function 6. Technical description: The function DepthFirstSearch solves for and returns a list of **paths** of adjacent segment codon combinations (adjacents, a dictionary passed to the function as an argument) which are assembled to form a larger sequence via a user-defined order of segments belonging to specific groupings (groupPath, a nested list of grouped-segment pairs passed to the function as an argument). For example, if groupPath is passed to the function as: [['A[1]', 'B[2]'], ['B[2]', 'C[3]'], ['C[3]', 'A[3]'], ['A[3]', 'B[1]'], ['B[1]', 'C[2]']], the search function will be constrained to codon combinations that assemble a path which has the structure: A[1]+B[2]+C[3]+A[3]+B[1]+C[2], where the character specifies the segment and the number in square parentheses indicates the group.

The function conducts this search by first assembling the set of all adjacent codon combinations for the current iteration (*curAdj*, Lines 10 through 32). If this is the first iteration of the search function (Lines 26 through 32) or if the current iteration visits a segment which was not present in the previous iteration (Lines 19 through 25) then the search function collects any adjacent codon combination satisfying the pair of segment and group constraints. However, if the search function must continue to assemble a sequence path through a contiguous set of segments (Lines 11 through 18) then the function must compile adjacent segment pairs which begin with the identical codon combination terminating the path from the previous iteration. If no adjacent segment codon combinations can be found (Line 33), then a failure counter is updated (fails, Line 34) and the terminal codon combination from the previous iteration is stored in a list (rejects, Lines 35 and 36) to be avoided in future solution paths. If the number of failed paths encountered (fails) exceeds the maximum allotment (failCount, Line 37) then the function terminates returning the set of all solutions (**paths**, Line 38) compiled to that instance. Should the search algorithm remain within the

maximum allotment of failed paths encountered, the function recursively calls itself (Lines 39 through 46) passing the updated list of rejected codon combinations to be avoided (rejects). Conversely, if the search function finds one or more adjacent codon combinations satisfying the segment and group constraints specified in groupPath for the current iteration, the function will randomly select an adjacent codon combination from the compiled list (*curAdj*, Line 48) to append it to the end of the path and continue on to the next iteration. Should a full-length path be assembled, the current path solution is appended onto a list of solutions (**paths**, Line 50). If the number of solved paths (goals) exceeds the user specified amount (goalCount) the function terminates returning the list of solved paths (Lines 51 and 52). However, if more paths may be searched, the function recursively calls itself updating the list of solved paths and the goal counter (Lines 54 through 59).

Function 7. FilterSegment

```
1  def FilterSegment(segment, T_FF=0.0, T_RR=0.0, T_FR=0.0, T_WT=0.0, ADJ=False,\
2                    idxT_FF=4, idxT_RR=5, idxT_FR=6, idxT_WT=7, delim=','):
3      output = []
4      for codon in segment:
5          text = ''
6          if ADJ:
7              if float(codon[idxT_FF]) <= T_FF and\
8                 float(codon[idxT_RR]) <= T_RR and\
9                 float(codon[idxT_FR]) >= T_FR:
10                 text = codon[0]
11         else:
12             if float(codon[idxT_FF]) <= T_FF and\
13                float(codon[idxT_RR]) <= T_RR and\
14                float(codon[idxT_FR]) >= T_FR and\
15                float(codon[idxT_WT]) <= T_WT:
16                 text = codon[0]
17         if text != '':
18             for it in codon[1:]:
19                 text = text + delim + str(it)
20             output.append(text)
21     return(output)
```

Function 7. Technical description: The function FilterSegment prunes a list of codon combinations passed to the function as an argument (segment, a nested list of lists with each entry containing a unique codon combination and its set of thermodynamic parameter values) based on a set of user specified thresholds. These thresholds passed to the function as keywords and are set as melting temperatures (Tm, °C) and include: the maximum Tm of homodimerization for the forward sequence (T_FF), the maximum Tm of homodimerization for the reverse complement sequence (T_RR), the minimum heterodimerization Tm between the forward and reverse complement sequence (T_FR), and the maximum Tm of hybridization between the forward sequence and the reverse complement sequence of the wild-type gene (T_WT). The function accesses each thermodynamic parameter value using an index provided by a set of keywords: index for the T_FF parameter (idxT_FF), index for the T_RR parameter (idxT_RR), index for the T_FR parameter (idxT_FR), and index for the T_WT parameter (idxT_WT). The function performs a comparison for the parameters T_FF, T_RR, and T_FR (Lines 7 through 9) if the keyword ADJ is True, and T_FF, T_RR, T_FR, and T_WT (Lines 12 through 15) if the ADJ keyword is False. The function returns the pruned list of codon combinations (**output**) with each entry included as a text string, delimited using a character keyword (delim, Lines 17 through 19), having the same organization as the list of codon combinations in the input argument segment.

Function 8. GenerateAdjacents

```
def GenerateAdjacents(segments, adjmap, idxName=0, idxFwd=1, idxRev=2,\
                      adjDelim='+', strDelim=','):
    output = []
    for iAdj, jAdj in adjmap:
        iSeq, jSeq = segments[iAdj], segments[jAdj]
```

```
6          for iVal in iSeq:
7              for jVal in jSeq:
8                  name = iVal[idxName] + adjDelim + jVal[idxName]
9                  fwd = iVal[idxFwd][:-1] + jVal[idxFwd]
10                 rev = jVal[idxRev][:-1] + iVal[idxRev]
11                 output.append(name + strDelim + fwd + strDelim + rev)
12     return(output)
```

Function 8. Technical description: The function GenerateAdjacents assembles the complete list of codon combination pairs (**output**) from a dictionary (segments, passed to the function as an argument) listing codon combinations keyed by their respective segment names. Specific pairs of segments are generated (Lines 6 through 11) specified by the user as an argument (adjmap, a nested list of lists containing pairs of segment names corresponding to the keys in the segments dictionary). The compiled list of adjacent codon combinations (**output**) is returned as a list of strings, with each entry containing a string of the codon pair name (a contraction using the names from each respective codon combination joined by the delimiter specified by the keyword adjDelim, Line 8), forward sequence, and reverse complementary sequence, delimited according to the keyword strDelim. Additional function keywords (idxName, idxFwd, and idxRev) indicate the index of the name, forward sequence, and reverse complement for each list of dictionary entries in segments.

Function 9. GenerateSegmentPairs

```
1  def GenerateSegmentPairs(codons, idxName=0, idxFwd=1, idxRev=2, strDelim=','):
3      output = []
4      for i in range(len(codons) + 1):
5          for j in range(i + 1, len(codons)):
6              count = 0
7              length = 0
8              for x, y in zip(codons[i][idxFwd][:-1], codons[j][idxFwd][:-1]):
9                  count = count + 1 if x == y else count + 0
10                 length = length + 1
11             count = count / length
12             output.append(codons[i][idxName] + strDelim +\
13                           codons[j][idxName] + strDelim +\
14                           codons[i][idxFwd] + strDelim +\
15                           codons[i][idxRev] + strDelim +\
16                           codons[j][idxFwd] + strDelim +\
17                           codons[j][idxRev] + strDelim + str(count))
18     return(output)
```

Function 9. Technical description: The function GenerateSegmentPairs compiles and returns a list of paired (Lines 4 and 5) codon combinations (**output**) organized as a list of delimited strings (strDelim) where each element includes the codon combination pair (idxName) and their forward (idxFwd) and reverse complement (idxFwd) sequences, in addition to a comparison of their percent identity (Lines 8 through 11). The function operates on codon combinations that belong to the same segment, passed to the function as an argument (codons, a list of codon combinations belonging to the same segment).

Function 10. GroupAnalysis

```
def GroupAnalysis(key, segments, pairs, iCmp=0, jCmp=1, ijBP=2, grpDelim='+',\
                  groupStart='[', groupEnd=']', segName=0, cos=0.950, prct=0.800):
    BP = dict()
    for compare in pairs:
        BP[compare[iCmp] + grpDelim + compare[jCmp]] = float(compare[ijBP])
        BP[compare[jCmp] + grpDelim + compare[iCmp]] = float(compare[ijBP])
    count = 1
    groupList = {segments[0][segName]: key + groupStart + str(count) + groupEnd}
    for segment in segments[1:]:
        name = segment[segName]
        notfound = True
```

```
12          updateGroup = ''
13          for groupKey in groupList:
14              if BP[name + grpDelim + groupKey] >= prct:
15                  vecName = []
16                  for it in segments:
17                      if it[0] is not name and it[0] is not groupKey:
18                          vecName.append(it[0])
19                  iBP = [BP[name + grpDelim + it] for it in vecName]
20                  jBP = [BP[groupKey + grpDelim + it] for it in vecName]
21                  cosValue = ComputeCosine(iBP, jBP)
22                  if cosValue >= cos:
23                      notfound = False
24                      updateGroup = groupList[groupKey]
25          if notfound:
26              count = count + 1
27              groupList[name] = str(key) + groupStart + str(count) + groupEnd
28          else:
29              groupList[name] = updateGroup
30      output = []
31      for segment in segments:
32          name = groupList[segment[segName]] +\
33                 segment[segName][segment[segName].index(codonDelim):]
34          content = ''
35          for it in range(len(segment)):
36              if it == segName:
37                  content = content + name + ','
38              else:
39                  content = content + segment[it] + ','
40          output.append(content[:-1])
41      return(output)
```

Function 10. Technical description: The function GroupAnalysis groups a list of codon combinations (segments, passed to this function as an argument) belonging to a specific protein segment (name identified by the argument key) by comparing the percent sequence identity between pairs of codon combinations (pairs, passed to this function as an argument). The function begins by compiling a dictionary (*BP*, Lines 4 through 6) to facilitate the rapid look-up of percent sequence identity values, by extracting the metric (ijBP, keyword indicates the nested index location in the pairs list) for the first (iCmp, keyword indicates the nested index location for the codon identifier in the pairs list) and second (jCmp, keyword indicates the nested index location for the codon identifier in the pairs list) codon combinations. The first codon combination in segments is automatically assigned to the first grouping (Line 8) while all successive codon combinations are subjected to the grouping analysis (Lines 9 through 29). This grouping analysis is conducted in two steps, with the first step identifying pairs of codon combinations which meet or exceed a threshold (set as the keyword prct) for percent sequence identity (Line 14). This is followed by a second step where the cosine similarity (function ComputeCosine, Line 21) is computed for the codon combination, using a pair of vectors generated by retrieving their percent sequence identity with the remaining codon combinations (*vecName*, Lines 16 through 18) in the segment (vectors *iBP* and *jBP*, Lines19 through 21). If the compared pair of codon combinations share similar percent sequence identities values with the remaining codon combinations in the segment (specified by the threshold set as the cos keyword for this function) then the pair of codon combinations are considered to belong to the same group (Lines 28 through 29). If the codon combination pair is considered to be distinct (*i.e.,* different groups) then the comparison is completed for the remaining codon combinations in the segment. If no grouping can be assigned to the codon combination, then the codon combination is assigned to its own unique group (Lines 25 through 27). This process is iterated until all codon combinations belonging to segments have been assigned a group identifier delimited between start (groupStart) and end (groupEnd) characters and formatted as a list (*groupList*) of these assignments. The function then inserts the corresponding group designation into each entry of segments, using the character string specified via the keyword codonDelim to identify the point of insertion, and returns a list of these concatenated coma delimited entries (**output**).

Function 11. ReadAdjacents

```
1  def ReadAdjacents(inputFile, name='Segment[Group](Codons)', fwd='FWD', rev='REV',\
2                     T_FF='T(FF)', T_RR='T(RR)', T_FR='T(FR)', adj='+', grp='['):
4      data = open(inputFile, 'r')
5      content = data.read()
6      content = content.split('\n')
7      header = content[0].split(',')
8      indx_name = header.index(name)
9      indx_fwd = header.index(fwd)
10     indx_rev = header.index(rev)
11     indx_ff = header.index(T_FF)
12     indx_rr = header.index(T_RR)
13     indx_fr = header.index(T_FR)
14     segments = dict()
15     for element in content[1:]:
16         element = element.split(',')
17         curr_name = element[indx_name]
18         key = curr_name.split(adj)
19         key = key[0][0:key[0].find(grp)] + adj + key[1][0:key[1].find(grp)]
20         curr_fwd = element[indx_fwd]
21         curr_rev = element[indx_rev]
22         curr_ff = element[indx_ff]
23         curr_rr = element[indx_rr]
24         curr_fr = element[indx_fr]
25         curr_list = [curr_name, curr_fwd, curr_rev, curr_ff, curr_rr, curr_fr]
26         if any(key == seg for seg in segments):
27             segments[key].append(curr_list)
28         else:
29             segments[key] = [curr_list]
30     data.close()
31     return(segments)
```

Function 11. Technical description: The function ReadAdjacents extracts and returns a dictionary of adjacent codon combinations (**segments**, dictionary containing adjacent codon combination lists keyed by their appended adjacent segment names, Lines 26 through 29) belonging to multiple segments from a .csv file (inputFile, detailing the file name and location passed to this function as an argument). This function utilizes keywords (name, fwd, rev, T_FF, T_RR, and T_FR for adjacent codon combination identifier, forward DNA sequence, reverse compliment DNA sequence, melting temperature for forward sequence homodimerization, melting temperature for reverse complement sequence homodimerization, and melting temperature for heterodimerization of the forward and reverse complement sequences, respectively) identical to the column headers in the first line of inputFile to index (Lines 7 through 13) and extract the corresponding information (Lines 15 through 25).

Function 12. ReadPairs

```
def ReadPairs(inputFile, iName='iSegment', jName='jSegment', BP='BP(n/N)',\
              delim='('):
    data = open(inputFile, 'r')
    content = data.read()
    content = content.split('\n')
    header = content[0].split(',')
    indx_iName = header.index(iName)
    indx_jName = header.index(jName)
    indx_bp = header.index(BP)
    pairs = dict()
    for element in content[1:]:
        element = element.split(',')
        curr_iName = element[indx_iName]
```

```
14         curr_jName = element[indx_jName]
15         curr_bp = element[indx_bp]
16         curr_seg = curr_iName[0:curr_iName.find(delim)]
17         curr_list = [curr_iName, curr_jName, curr_bp]
18         if any(key == curr_seg for key in pairs):
19             pairs[curr_seg].append(curr_list)
20         else:
21             pairs[curr_seg] = [curr_list]
22     data.close()
23     return(pairs)
```

Function 12. Technical description: The function ReadPairs extracts and returns a dictionary of paired codon combinations (**pairs**, dictionary containing paired codon combination lists keyed by their segment name, Lines 18 through 21) belonging to multiple segments from a .csv file (inputFile, detailing the file name and location passed to this function as an argument). This function utilizes keywords (iName, jName, and BP for the codon combination identifier of the first segment and second segment in addition to the percent sequence identity, respectively) identical to the column headers in the first line of inputFile to index (Lines 7 through 9) and extract the corresponding information (Lines 11 through 17).

Function 13. ReadPaths

```
1  def ReadPaths(inputFile, cassettes, delimLine='\n', delimEntry=','):
2      data = open(inputFile, 'r')
3      content = data.read()
4      content = content.split(delimLine)
5      header = content[0].split(delimEntry)[1:]
6      unique, casslist = [], []
7      for it in header:
8          unique.append(it) if it not in unique else None
9      for cas in cassettes:
10         for uni in unique:
11             casslist.append(cas)
12     paths = dict()
13     for element in content[1:]:
14         element = element.split(delimEntry)
15         entryDict = {key: {} for key in header}
16         for key, cas, val, in zip(header, casslist, element[1:]):
17             entryDict[key][cas] = val
18         paths[element[0]] = entryDict
19     data.close()
20     return(paths)
```

Function 13. Technical description: The function ReadPaths extracts and returns a dictionary containing segment sequences that reconstitute segment paths that assemble the DNA template for the TR gene (**paths**, *triple*-nested set of dictionary objects keyed by path, segment, and cassette identifiers, Lines 17 and 18) from an input .csv file (inputFile, detailing the file name and location passed to this function as an argument). This function employs a list of strings defining the names of TR cassettes (cassettes, passed to the function as an argument) whose elements are duplicated for every TR segment (*casslist*, Lines 7 through 11). This function utilizes keywords the delimLine and delimEntry to provide instruction on how to interpret file contents defining characters representing new lines (*e.g.*, '\n') and characters delimiting column entries (*e.g.*, ','). Path index (*element*, Line 13) in addition to segments (*key*), cassettes (*cas*), and their sequences (*val*) are iteratively extracted (Line 16) and reorganized for compilation into the output dictionary **paths** (Line 18).

Function 14. ReadSegments

```
def ReadSegments(inputFile, delim='(', name='Segment(Codons)', fwd='FWD',\
                 rev='REV', FRQ='FRQ', T_FF='T(FF)', T_RR='T(RR)', T_FR='T(FR)',\
                 T_WT='T(WT)'):
    data = open(inputFile, 'r')
```

```
5      content = data.read()
6      content = content.split('\n')
7      header = content[0].split(',')
8      indx_name = header.index(name)
9      indx_fwd = header.index(fwd)
10     indx_rev = header.index(rev)
11     indx_fq = header.index(FRQ)
12     indx_ff = header.index(T_FF)
13     indx_rr = header.index(T_RR)
14     indx_fr = header.index(T_FR)
15     indx_wt = header.index(T_WT)
16     segments = dict()
17     for element in content[1:]:
18         element = element.split(',')
19         curr_name = element[indx_name]
20         curr_seg = curr_name[0:curr_name.find(delim)]
21         curr_fwd = element[indx_fwd]
22         curr_rev = element[indx_rev]
23         curr_fq = element[indx_fq]
24         curr_ff = element[indx_ff]
25         curr_rr = element[indx_rr]
26         curr_fr = element[indx_fr]
27         curr_wt = element[indx_wt]
28         curr_list = [curr_name, curr_fwd, curr_rev, curr_fq, curr_ff, curr_rr,\
29                      curr_fr, curr_wt]
30         if any(key == curr_seg for key in segments):
31             segments[curr_seg].append(curr_list)
32         else:
33             segments[curr_seg] = [curr_list]
34     data.close()
35     return(segments)
```

Function 14. Technical description: The function ReadSegments extracts and returns a dictionary of codon combinations (**segments**, dictionary containing codon combination lists keyed by their segment name, Lines 30 through 33) belonging to multiple segments from a .csv file (inputFile, detailing the file name and location passed to this function as an argument). This function utilizes keywords (name, FRQ, fwd, rev, T_FF, T_RR, T_FR, and T_WT for codon combination identifier, average codon frequency, forward DNA sequence, reverse compliment DNA sequence, melting temperature for forward sequence homodimerization, melting temperature for reverse complement sequence homodimerization, melting temperature for heterodimerization of the forward and reverse complement sequences, and melting temperature of the codon combination with the reverse complement sequence belonging to the wild-type sequence, respectively) identical to the column headers in the first line of inputFile to index (Lines7 through 15) and extract the corresponding information (Lines 17 through 29).

Function 15. RecursiveCombination

```
def RecursiveCombination(elements, vector=[[]], iteration=0):
    if iteration >= len(elements):
        return(vector)
    else:
        output = list()
        for vec in vector:
            for element in range(int(elements[iteration])):
                entry = list(vec) + list(str(element + 1))
                output.append(entry)
        iteration = iteration + 1
        return(RecursiveCombination(elements, vector=output, iteration=iteration))
```

Function 15. Technical description: The function RecursiveCombination exhaustively generates and returns a nested list (**vector**, passed to this function as a keyword) of all combinations for a set of integer values (elements, a list of integers passed to this function as an argument) indicating the maximum number of choices belonging to each element in **vector**. The function operates by successively appending (Lines 4 through 11) all choices for the current element indexed using the keyword iteration to the list of all **vectors**. The function returns the completed list of combinations when the current iteration count is equal to the length of each combination vector (Lines 2 and 3).

Function 16. WriteSegmentCodons

```
1  def WriteSegmentCodons(outputFile, outputData, outputHeader):
2      outputFile = open(outputFile, 'w')
3      outputFile.write(outputHeader)
4      for output in outputData:
5          outputFile.write('\n' + ''.join(output))
6      outputFile.close()
11     return(None)
```

Function 16. Technical description: The function WriteSegmentCodons creates a file (outputFile, a character string specifying the location and name of an output file passed to this function as an argument, Line 2) and writes (Line 3) the content in outputData (a character string passed to this function as an argument) to the first line in outputFile. The function then writes (Lines 4 and 5) the character strings belonging to outputData (a list passed to this function as an argument) with each element of the list written to a new line in the outputFile.

Function 17. WritePaths

```
1  def WritePaths(outputFile, paths, delimPair='+', delimGroup='['):
2      outputFile = open(outputFile, 'w')
3      header = 'Index'
4      for element in paths[0].split(delimPair):
5          header = header + ',Segment.' + element[:element.index(delimGroup)]
6      outputFile.write(header)
7      it = 0
8      for path in paths:
9          it = it + 1
10         line = '\n' + str(it) + ',' + path.replace(delimPair, ',')
11         outputFile.write(line)
12     outputFile.close()
13     return(None)
```

Function 17. Technical description: The function WritePaths creates a file (outputFile, a character string specifying the location and name of an output file passed to this function as an argument, Line 2) and writes the set of paths (a list of character strings passed to this function as an argument, Lines 8 through 11) to the file. The file has a specific comma delimited organization, with the index of each path proceeded by the segment names, group identifiers (delimGroup, a keyword indicating the character string used to identify the group designation), and codon combinations for each segment in the path (delimPair, a keyword indicating the character string used to distinguish successive segments belonging to the same path), as specified by the header written in the first line of the file (Lines 3 through 6).

Function 18. WriteSequences

```
def WriteSequences(outputFile, outputData):
    outputFile = open(outputFile, 'w')
    header = 'Index'
    for key in outputData[list(outputData.keys())[0]]:
        header = header + ',' + key
    outputFile.write(header)
    for key in outputData:
        outputFile.write('\n' + key)
        for frg in outputData[key]:
```

```
10              outputFile.write(',' + outputData[key][frg])
11      outputFile.close()
12      return(None)
```

Function 18. Technical description: The function WriteSequences creates a file (outputFile, a character string specifying the location and name of an output file passed to this function as an argument, Line 2) and iteratively (Lines 7 through 10) writes the DNA sequence content from a nested dictionary (outputData, passed to this function as an argument and keyed by path and fragment names) to the file.

Section 2. TReSR Results, Thermodynamic Parameters for PCR Primers, and Synthesis of scDBD Constructs

Organization of the Supplementary Information for TReSR Implementation. The main text discussion of TReSR algorithm implementation (*i.e.,* Implementation of the TReSR Protocol) is supported with supplementary results included in this section. Specifically, a summary of the TReSR output file data is provided in supplementary tables and figures, detailing:

1. the values for thermodynamic parameter thresholds employed to prune sequences from each segment and the remaining number of filtered sequences (S1 Table),
2. the number of segment group sets identified by grouping analysis with the group identity and sequence count for selected groups used in the remainder of the calculation (S1 Table),
3. the values for thermodynamic parameter thresholds employed to prune sequences from paired adjacent segments and the remaining number of filtered sequences (S2 Table),
4. the thermodynamic parameters for solution sequences designed by TReSR for each segment (S3 Table), and
5. the melting temperatures for hybridization between primers designed by TReSR (S1 Fig).

Organization of the Supplementary Information for PCR Synthesis. The main text discussion describing the PCR synthesis of tandem repeat repressor DNA sequences (*i.e.,* Synthesis of the Tandem Repeat Repressor) is supported with supplementary results included in this section. Specifically, a summary of the PCR strategy is provided in supplementary figures, detailing:

1. the map of primers and flowchart describing their use in the PCR strategy for assembling tandem repeat repressor DNA sequences from constituent fragments (S2 Fig),
2. the agarose gel electrophoresis results documenting the PCR synthesis of tandem repeat repressor DNA sequences (S3 Fig), and
3. the annotated sequences for tandem repeat repressors confirmed by Sanger sequencing (S4 Fig).

S1 Table. Thermodynamic parameter thresholds and grouping analysis for segment codon combinations

scDBD Segments			Parameter Thresholds				Pruned Codon Combinations			Grouping Analysis[D]		
Label	Residues	Sequence	T_{FF}[A] (°C)	T_{RR}[A] (°C)	T_{FR}[B] (°C)	T_{WT}[A] (°C)	Before Filter (N)	After Filter (n)	Fraction Discarded[C]	Total Groups	Selected Groups	Codon Combinations
A	1−6	MKPVTL	1.2	11.1	67.2	44.0	768	225	0.707	8	1 3 4 6	132
B	7−11	YDVAE	13.7	9.2	64.5	45.5	128	31	0.758	6	1 2 3 6	20
C	12−16	YAGVS	26.3	10.6	67.2	43.7	768	171	0.777	43	6 13 16 27	10
D	17−22	DFTVST	4.3	10.9	63.4	17.6	1,536	217	0.859	20	1 5 15 18	51
E	23−29	VVNQASH	19.8	26.9	71.6	49.8	3,072	651	0.788	9	2 4 5 7	417
F	60−64	QSLLI	−7.4	1.2	54.7	9.9	1,296	243	0.812	83	12 23 69 76	80
G	65−69	GVATS	26.1	23.7	66.5	43.8	1,536	287	0.813	106	1 22 46 76	14
H	70−74	SLALH	4.9	10.2	63.5	32.7	1,728	344	0.801	129	8 45 72 85	18
I	75−79	APSQI	−7.5	−2.6	64.9	24.3	576	138	0.760	43	3 16 33 40	22
J	80−84	VAAIK	24.8	22.3	61.4	33.1	384	78	0.797	16	4 5 6 15	24
K	85−89	SRADQ	26.2	23.9	67.1	45.4	576	152	0.736	58	6 15 26 45	28

[A]T_{FF}, T_{RR}, and T_{WT} parameter thresholds set by evaluating the 50th percentile across all codon combinations generated for the segment.
[B]T_{FR} parameter thresholds set by evaluating the 10th percentile across all codon combinations for each segment.
[C]Fraction of codon combinations removed in the filter step, calculated using 1 – (n ÷ N).
[D]Group assignments made by comparison of percent identity between pairs of codon combinations for each segment, with codon combination pairs sharing ≥ 80% sequence identity and cosine similarity values ≥ 0.9975 designated as belonging to the same group.

S2 Table. Thermodynamic parameter thresholds for adjacent segment codon combinations

Adjacent scDBD Segments			Parameter Thresholds (°C)			Pruned Codon Combinations		
Label	Residues	Sequence	T_{FF}[A]	T_{RR}[A]	T_{FR}[B]	Before Filter (N)	After Filter (n)	Fraction Discarded
A + B	1 − 11	MKPVTLYDVAE	38.4	34.3	80.0	2,640	1,981	0.250
B + C	7 − 16	YDVAEYAGVS	35.3	32.3	80.0	200	146	0.270
C + D	12 − 22	YAGVSDFTCST	40.7	34.5	80.0	510	404	0.208
D + E	17 − 29	DFTVSTVVNQASH	43.1	45.6	80.0	21,267	16,163	0.240
F + G	60 − 69	QSLLIGVATS	37.1	34.7	80.0	1,120	678	0.395
G + H	65 − 74	GVATSSLALH	35.5	36.0	80.0	252	191	0.242
H + I	70 − 79	SLALHAPSQI	35.8	40.2	80.0	396	344	0.131
I + J	75 − 84	APSQIVAAIK	28.4	33.9	80.0	528	388	0.265
J + K	80 − 89	VAAIKSRADQ	36.5	34.7	80.0	672	521	0.225
K + A	85 − 6	SRADQMKPVTL	36.7	37.1	80.0	3,696	2,882	0.220

[A]T_{FF} and T_{RR} parameter thresholds set by evaluating the 20th percentile across codon combinations
[B]T_{FR} parameter threshold set to a fixed value of 80.0 °C

S3 Table. Calculated thermodynamic parameters for TReSR designed DNA segments

scDBD Segment			DNA Sequence (5′ → 3′)	T_m Values (°C)			
Designation[A]	Residues	Sequence		T_{FF}	T_{RR}	T_{FR}	T_{WT}
A[6](1.2.1.1.2.6)	1 − 6	MKPVTL	ATG AAG CCT GTT ACC CTG	−24.2	−15.2	71.7	29.7
B[6](1.2.4.3.2)	7 − 11	YDVAE	TAT GAC GTG GCA GAG	13.7	−26.8	69.0	37.3
C[16](1.3.3.4.6)	12 − 16	YAGVS	TAT GCA GGA GTG AGC	10.2	−1.2	69.1	33.8
D[1](1.1.4.1.6.4)	17 − 22	DFTVST	GAT TTT ACG GTT AGC ACG	−4.5	−7.4	68.1	−0.8
E[7](3.3.2.1.2.2.1)	23 − 29	VVNQASH	GTA GTA AAC CAA GCC TCC CAT	−24.2	−3.1	74.4	39.5
F[23](1.2.6.4.1)	60 − 64	QSLLI	CAA TCC CTG CTC ATT	−7.4	−4.5	65.1	−15.4
G[46](3.2.1.3.4)	65 − 69	GVATS	GGA GTC GCT ACA TCG	10.6	19.6	67.9	18.1
H[45](2.3.2.1.2)	70 − 74	SLALH	TCC CTT GCC TTA CAC	−18.9	−4.3	68.3	14.7
I[16](2.2.1.1.2)	75 − 79	APSQI	GCC CCC TCT CAA ATC	−58.9	−43.7	68.6	11.7
J[4](3.1.2.3.2)	80 − 84	VAAIK	GTA GCT GCC ATA AAG	18.8	−1.6	62.5	3.2
K[6](3.6.1.2.1)	85 − 89	SRADQ	TCA AGG GCT GAC CAA	−9.3	−24.2	70.9	4.3
A[4](1.1.2.2.1.2)	1 − 6	MKPVTL	ATG AAA CCC GTC ACT TTG	−14.5	−2.6	70.2	36.2
B[1](1.1.3.2.1)	7 − 11	YDVAE	TAT GAT GTA GCC GAA	−12.4	−10.9	64.5	33.8
C[13](1.3.2.3.5)	12 − 16	YAGVS	TAT GCA GGC GTA AGT	18.7	−1.2	68.7	29.6
D[15](2.2.2.2.1.1)	17 − 22	DFTVST	GAC TTC ACC GTC TCT ACT	−9.0	−5.4	69.9	12.6
E[5](3.2.1.2.4.5.1)	23 − 29	VVNQASH	GTA GTC AAT CAG GCG AGT CAT	−6.4	−35.1	74.1	43.7
F[76](2.6.3.2.3)	60 − 64	QSLLI	CAG AGC CTT TTG ATA	−24.2	−21.9	62.9	2.2
G[76](4.3.3.4.5)	65 − 69	GVATS	GGG GTA GCA ACG AGT	1.2	12.7	70.5	17.3
H[8](3.2.3.4.1)	70 − 74	SLALH	TCA TTG GCA CTC CAT	−7.4	−6.1	68.1	17.1
I[3](3.1.2.2.3)	75 − 79	APSQI	GCA CCT TCC CAG ATA	−27.4	−36.5	68.8	−7.0
J[15](4.3.3.2.1)	80 − 84	VAAIK	GTG GCA GCA ATC AAA	15.0	17.8	66.9	10.9
K[15](4.5.4.2.2)	85 − 89	SRADQ	TCG AGA GCG GAC CAG	10.7	−12.7	73.8	7.8

[A]Tandem repeat segment designations organized by: segment name, [grouping number], and (codon combination index numbers)

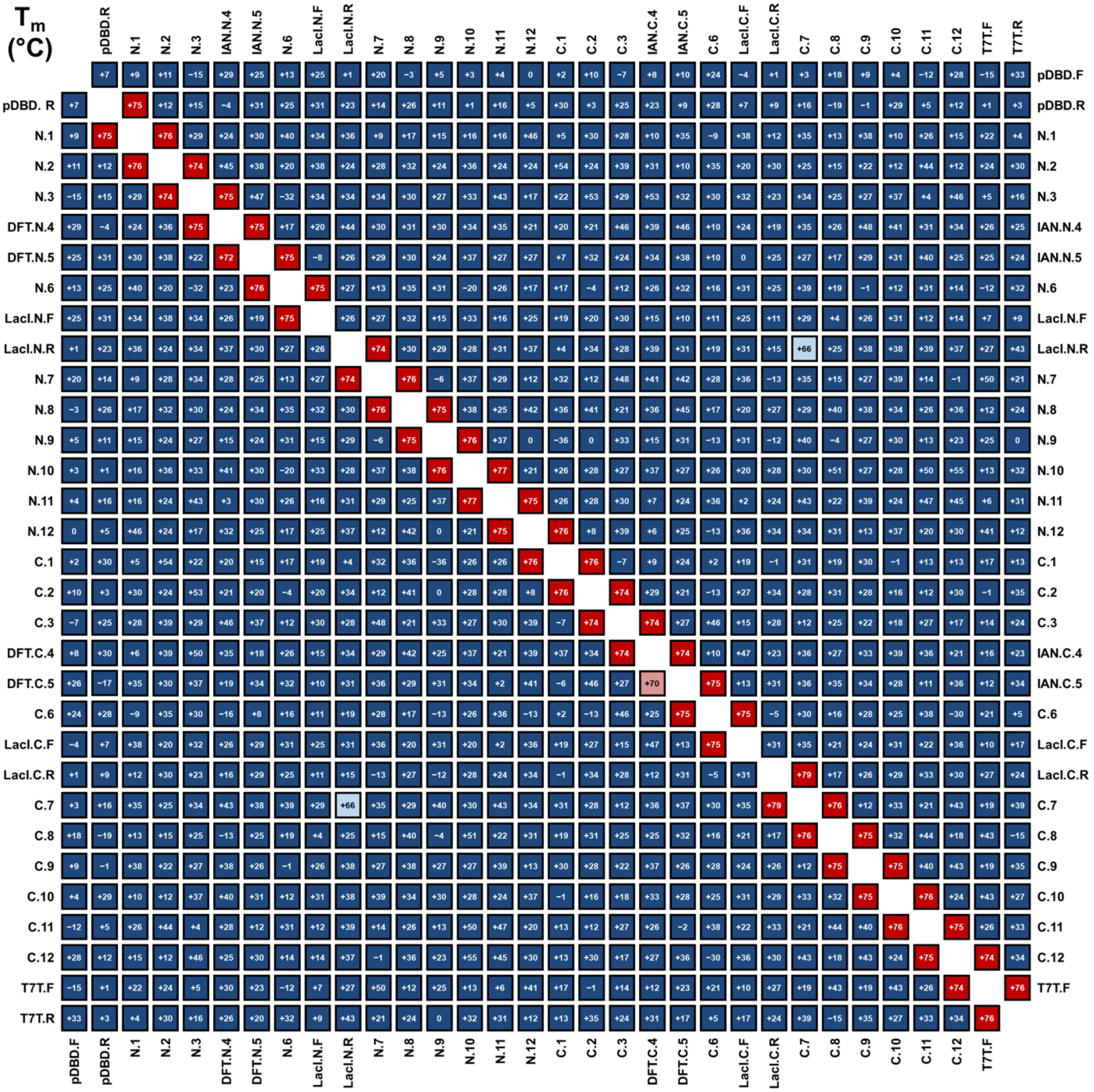

S1 Fig. Calculated hybridization melting temperatures for aPCR primers. The predicted melting temperature (Tm) for hybridization between pairs of primers used in assembly PCR synthesis of N- and C-terminal DNA binding domains (primers labelled N and C, respectively) and their splicing by overlap extension to form the single-chain tandem repeat repressor construct. Predicted Tm values were calculated for primer sequences reported in Table 1 constructing DNA binding domains having the DFT (D17/F18/T22, bottom-left triangle) or IAN (I17/A18/N22, top-right triangle) triple-mutations, using the UNAFold webserver Two State Melting Hybridization application (http://www.unafold.org/Dinamelt/applications/two-state-melting-hybridization.php). Predicted Tm values are coloured according to their stability, with primer pairs expected to hybridize under PCR conditions indicated in red (Tm ≥ 68 °C, the PCR annealing temperate), and primer pairs predicted unable to associate under PCR conditions in blue (Tm < 68 °C). Assembly PCR primers are numbered according to their sequence orientation in the gene, with odd numbered primers encoding template forward sequences, and even numbered primers encoding template reverse complement sequences.

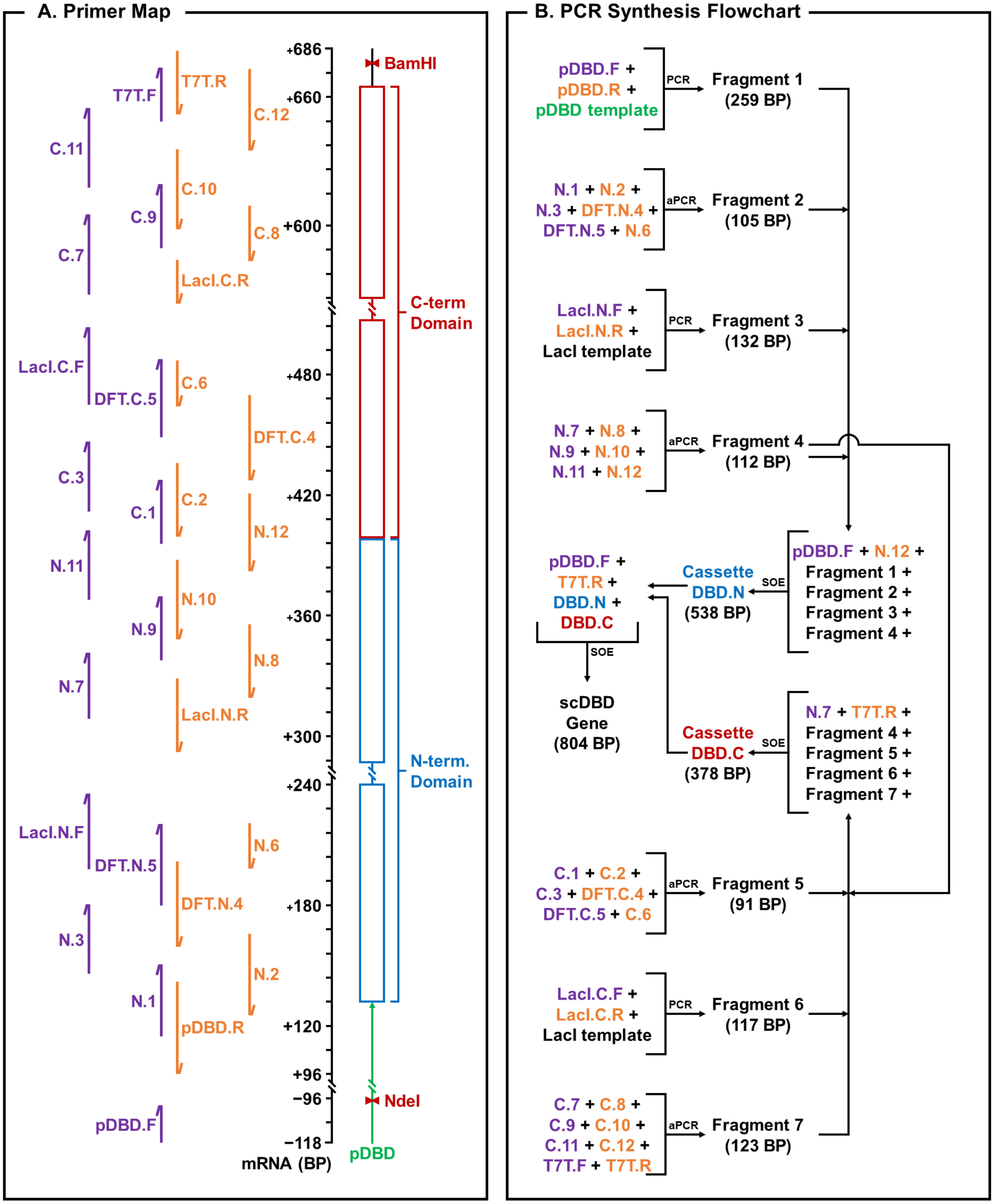

S2 Fig. Assembly PCR and SOE synthesis workflow. Map (A) indicating position (relative to initiation site of mRNA transcript) and orientation (forward and reverse complement directions in purple and orange, respectively) of assembly PCR primers used to construct the single-chain tandem repeat repressor. Flowchart (B) for single-chain tandem repeat repressor synthesis from DNA fragments (1 *through* 7) constructed using assembly PCR primers and their subsequent incorporation into N-terminal (blue) and C-terminal (red) DNA binding domain cassettes by SOE. Length of DNA constructs indicated in parentheses.

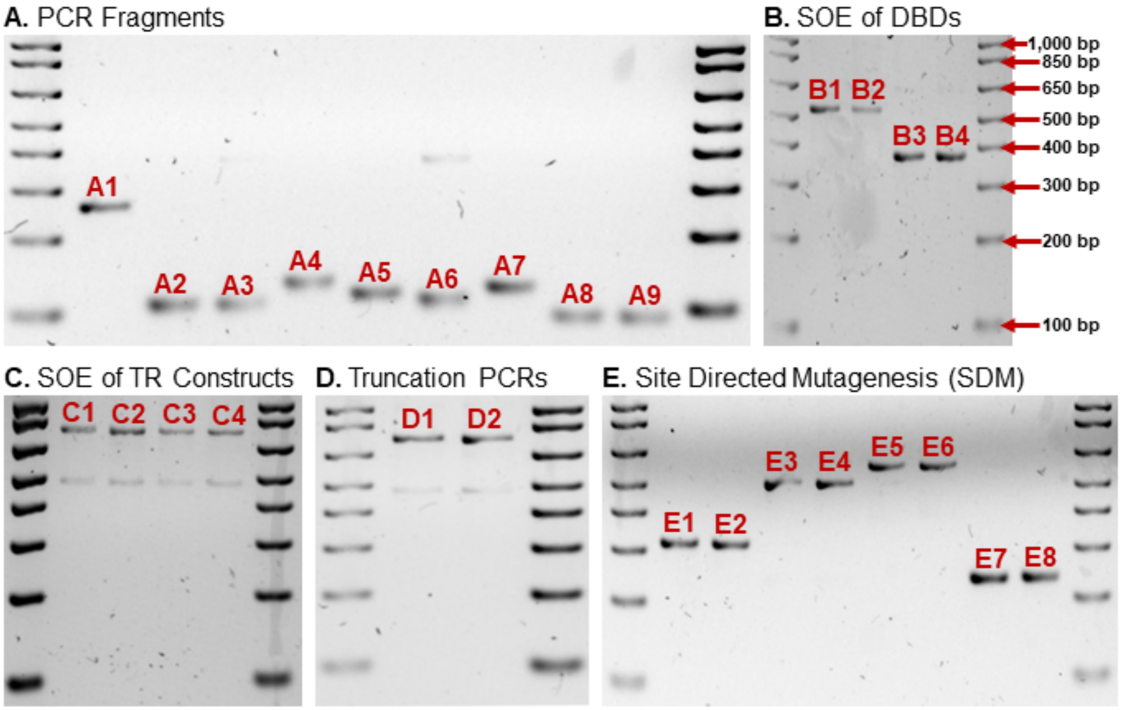

Band	Amplicon	Exp (bp)	Obs (bp)
A1	Fragment 1	259	257 ± 10
A2	Fragment 2 (IAN)	105	108 ± 4
A3	Fragment 2 (DFT)	105	107 ± 4
A4	Fragment 3	132	135 ± 5
A5	Fragment 6	117	119 ± 4
A6	Fragment 4	112	115 ± 4
A7	Fragment 7	123	126 ± 4
A8	Fragment 5 (IAN)	91	94 ± 3
A9	Fragment 5 (DFT)	91	93 ± 3
B1	Cassette DBD.N (IAN)	538	544 ± 28
B2	Cassette DBD.N (DFT)	538	546 ± 28
B3	Cassette DBD.C (IAN)	378	378 ± 19
B4	Cassette DBD.C (DFT)	378	379 ± 19

Band	Amplicon	Exp (bp)	Obs (bp)
C1	scDBD$_{IAN/IAN}$	804	801 ± 39
C2	scDBD$_{DFT/DFT}$	804	796 ± 38
C3	scDBD$_{IAN/DFT}$	804	799 ± 38
C4	scDBD$_{DFT/IAN}$	804	800 ± 38
D1	scDBD$_{IAN/IAN/\Delta CT}$	744	754 ± 29
D2	scDBD$_{DFT/DFT/\Delta CT}$	744	752 ± 29
E1	SDM: pDBD.F + IAN.N.4	319	315 ± 11
E2	SDM: pDBD.F + DFT.N.4	319	314 ± 11
E3	SDM: IAN.N.5 + T7T.R	508	515 ± 18
E4	SDM: DFT.N.5 + T7T.R	507	514 ± 18
E5	SDM: pDBD.F + IAN.C.4	586	592 ± 20
E6	SDM: pDBD.F + DFT.C.4	586	591 ± 20
E7	SDM: IAN.C.5 + T7T.R	238	235 ± 8
E8	SDM: DFT.C.5 + T7T.R	238	236 ± 8

S3 Fig. Agarose gel electrophoresis of PCR products used in synthesis of scDBD constructs. PCR products listed by expected (Exp) and observed (Obs) length examined by 2% ($^{w}/_{v}$) agarose gel electrophoresis against Invitrogen 1 Kb Plus DNA ladder. The synthesis of single-chain tandem repeat repressor constructs was conducted in three steps, starting first with synthesis and gel purification of gene fragments (A) encoding the promoter (A1) and template sequence unaltered fragments (A4 and A5) by PCR amplification using the plasmid pDBD(eGFP)·pGFP(dGFP)·pLacI(LacI$_{W220F}$). Synthesis of these fragments was accompanied by the assembly PCR and gel purification of gene fragments using primers designed by the TReSR protocol producing Fragments 2 (A2 and A3) and 5 (A8 and A9) encoding LacI residues 1 through 30, delivering the DFT (D17/F18/T22) and IAN (I17/A18/N22) triple-mutations, respectively, and Fragments 3 (A4) and 7 (A7) encoding LacI residues 60 through 89. Cassettes (B) encoding N-terminal (B1 and B2) and C-terminal (B3 and B4) duplicated DNA binding domains were constructed in a second step by SOE PCR using the corresponding gene fragments from the first step. The third step concludes the synthesis with SOE of DNA binding domain cassettes producing full-length single-chain tandem repeat repressor constructs (C) outfitted with combinations of N- and C-terminal domain *triple*-mutations, including: IAN/IAN (C1), DFT/DFT (C2), IAN/DFT (C3), and DFT/IAN (C4). PCR synthesis includes C-terminal truncations (ΔCT) of full-length tandem repeat constructs (D1 and D2) and site-directed mutagenesis (SDM) of N-terminal and C-terminal DNA binding domain cassettes (E1 through E8) demonstrates and confirms selective PCR manipulation is achieved using TReSR DNA sequence templates.

A. scDBD$_{\text{DFT/DFT}}$

-118									CCAGTAGT	AGGTTGAGGC
-100	CATATGCTAA	AGGTTGGTCT	AGGCACATAC	CATTCTTGTT	CTGTTGCGAA	CGAACCTTGG	CTGTC**TTGAC**	**AATTGTGAGC**	**GCTCACAAGA**	**TACTGTGTGG**
1	**AATTGTGAGC**	**GCTCACAATT**	TCCTATGGTG	CAGAGTCGAG	CTGTCACCGG	ATGTGCTTTC	CGGTCTGATG	AGTCCGTGAG	GACGAAACAG	CCTCTACAAA
101	TAATTTTGTT	TAAACTAGAA	GGAGGAAAAA	AATGAAGCCT	GTTACCCTGT	ATGACGTGGC	AGAGTATGCA	GGAGTGAGCG	ATTTTACGGT	TAGCACGGTA
201	GTAAACCAAG	CCTCCCATGT	TTCTGCGAAA	ACGCGGGAAA	AAGTGGAAGC	GGCGATGGCG	GAGCTGAATT	ACATTCCCAA	CCGCGTGGCA	CAACAACTGG
301	CGGGCAAACA	ATCCCTGCTC	ATTGGAGTCG	CTACATCGTC	CCTTGCCTTA	CACGCCCCCT	CTCAAATCGT	AGCTGCCATA	AAGTCAAGGG	CTGACCAAAT
401	GAAACCCGTC	ACTTTGTATG	ATGTAGCCGA	ATATGCAGGC	GTAAGTGACT	TCACCGTCTC	TACTGTAGTC	AATCAGGCGA	GTCATGTTTC	TGCGAAAACG
501	CGGGAAAAAG	TGGAAGCGGC	GATGGCGGAG	CTGAATTACA	TTCCCAACCG	CGTGGCACAA	CAACTGGCGG	GCAAACAGAG	CCTTTTGATA	GGGGTAGCAA
601	CGAGTTCATT	GGCACTCCAT	GCACCTTCCC	AGATAGTGGC	AGCAATCAAA	TCGAGAGCGG	ACCAGTAATG	ATAGGGATCC	GGCTGC	

B. scDBD$_{\text{IAN/IAN}}$

-118									CCAGTAGT	AGGTTGAGGC
-100	CATATGCTAA	AGGTTGGTCT	AGGCACATAC	CATTCTTGTT	CTGTTGCGAA	CGAACCTTGG	CTGTC**TTGAC**	**AATTGTGAGC**	**GCTCACAAGA**	**TACTGTGTGG**
1	**AATTGTGAGC**	**GCTCACAATT**	TCCTATGGTG	CAGAGTCGAG	CTGTCACCGG	ATGTGCTTTC	CGGTCTGATG	AGTCCGTGAG	GACGAAACAG	CCTCTACAAA
101	TAATTTTGTT	TAAACTAGAA	GGAGGAAAAA	AATGAAGCCT	GTTACCCTGT	ATGACGTGGC	AGAGTATGCA	GGAGTGAGCA	TTGCCACGGT	CTCTAATGTA
201	GTAAACCAAG	CCTCCCATGT	TTCTGCGAAA	ACGCGGGAAA	AAGTGGAAGC	GGCGATGGCG	GAGCTGAATT	ACATTCCCAA	CCGCGTGGCA	CAACAACTGG
301	CGGGCAAACA	ATCCCTGCTC	ATTGGAGTCG	CTACATCGTC	CCTTGCCTTA	CACGCCCCCT	CTCAAATCGT	AGCTGCCATA	AAGTCAAGGG	CTGACCAAAT
401	GAAACCCGTC	ACTTTGTATG	ATGTAGCCGA	ATATGCAGGC	GTAAGTATCG	CTACAGTGAG	CAATGTAGTC	AATCAGGCGA	GTCATGTTTC	TGCGAAAACG
501	CGGGAAAAAG	TGGAAGCGGC	GATGGCGGAG	CTGAATTACA	TTCCCAACCG	CGTGGCACAA	CAACTGGCGG	GCAAACAGAG	CCTTTTGATA	GGGGTAGCAA
601	CGAGTTCATT	GGCACTCCAT	GCACCTTCCC	AGATAGTGGC	AGCAATCAAA	TCGAGAGCGG	ACCAGTAATG	ATAGGGATCC	GGCTGC	

C. scDBD$_{\text{DFT/DFT/}\Delta\text{CT}}$

-118									CCAGTAGT	AGGTTGAGGC
-100	CATATGCTAA	AGGTTGGTCT	AGGCACATAC	CATTCTTGTT	CTGTTGCGAA	CGAACCTTGG	CTGTC**TTGAC**	**AATTGTGAGC**	**GCTCACAAGA**	**TACTGTGTGG**
1	**AATTGTGAGC**	**GCTCACAATT**	TCCTATGGTG	CAGAGTCGAG	CTGTCACCGG	ATGTGCTTTC	CGGTCTGATG	AGTCCGTGAG	GACGAAACAG	CCTCTACAAA
101	TAATTTTGTT	TAAACTAGAA	GGAGGAAAAA	AATGAAGCCT	GTTACCCTGT	ATGACGTGGC	AGAGTATGCA	GGAGTGAGCG	ATTTTACGGT	TAGCACGGTA
201	GTAAACCAAG	CCTCCCATGT	TTCTGCGAAA	ACGCGGGAAA	AAGTGGAAGC	GGCGATGGCG	GAGCTGAATT	ACATTCCCAA	CCGCGTGGCA	CAACAACTGG
301	CGGGCAAACA	ATCCCTGCTC	ATTGGAGTCG	CTACATCGTC	CCTTGCCTTA	CACGCCCCCT	CTCAAATCGT	AGCTGCCATA	AAGTCAAGGG	CTGACCAAAT
401	GAAACCCGTC	ACTTTGTATG	ATGTAGCCGA	ATATGCAGGC	GTAAGTGACT	TCACCGTCTC	TACTGTAGTC	AATCAGGCGA	GTCATGTTTC	TGCGAAAACG
501	CGGGAAAAAG	TGGAAGCGGC	GATGGCGGAG	CTGAATTACA	TTCCCAACCG	CGTGGCACAA	CAACTGGCGG	GCAAACAGAG	CCTTTTGATA	GGGGTAGCAA
601	CGAGTTAATG	ATAGGGATCC	GGCTGC							

D. scDBD$_{\text{IAN/IAN/}\Delta\text{CT}}$

-118									CCAGTAGT	AGGTTGAGGC
-100	CATATGCTAA	AGGTTGGTCT	AGGCACATAC	CATTCTTGTT	CTGTTGCGAA	CGAACCTTGG	CTGTC**TTGAC**	**AATTGTGAGC**	**GCTCACAAGA**	**TACTGTGTGG**
1	**AATTGTGAGC**	**GCTCACAATT**	TCCTATGGTG	CAGAGTCGAG	CTGTCACCGG	ATGTGCTTTC	CGGTCTGATG	AGTCCGTGAG	GACGAAACAG	CCTCTACAAA
101	TAATTTTGTT	TAAACTAGAA	GGAGGAAAAA	AATGAAGCCT	GTTACCCTGT	ATGACGTGGC	AGAGTATGCA	GGAGTGAGCA	TTGCCACGGT	CTCTAATGTA
201	GTAAACCAAG	CCTCCCATGT	TTCTGCGAAA	ACGCGGGAAA	AAGTGGAAGC	GGCGATGGCG	GAGCTGAATT	ACATTCCCAA	CCGCGTGGCA	CAACAACTGG
301	CGGGCAAACA	ATCCCTGCTC	ATTGGAGTCG	CTACATCGTC	CCTTGCCTTA	CACGCCCCCT	CTCAAATCGT	AGCTGCCATA	AAGTCAAGGG	CTGACCAAAT
401	GAAACCCGTC	ACTTTGTATG	ATGTAGCCGA	ATATGCAGGC	GTAAGTATCG	CTACAGTGAG	CAATGTAGTC	AATCAGGCGA	GTCATGTTTC	TGCGAAAACG
501	CGGGAAAAAG	TGGAAGCGGC	GATGGCGGAG	CTGAATTACA	TTCCCAACCG	CGTGGCACAA	CAACTGGCGG	GCAAACAGAG	CCTTTTGATA	GGGGTAGCAA
601	CGAGTTAATG	ATAGGGATCC	GGCTGC							

S4 Fig. Annotated sequences encoding single-chain tandem repeat repressor constructs. Genes for full-length scDBD$_{\text{DFT/DFT}}$ (A) and scDBD$_{\text{IAN/IAN}}$ (B) and C-terminal truncated scDBD$_{\text{DFT/DFT/}\Delta\text{CT}}$ (C) and scDBD$_{\text{IAN/IAN/}\Delta\text{CT}}$ (D) single-chain tandem repeat repressor constructs confirmed by Sanger sequencing. All genes are indexed by base-pair relative to the pDBD promoter cassette (green) mRNA transcription initiation site (first base-pair) contained within the mRNA polymerase recruitment and lacO^{sym} operator sites (bold green). Sequences belonging to tandem repeat duplicated domains are coloured according to their N-terminal (blue) or C-terminal (purple) position in the gene. Restriction sites unique to cloning Site I, NdeI (−100 to −95 bp) and BamHI (615 to 620 bp), are indicated in red.

Section 3. Engineering and Optimization of the *three*-Component Genetic Circuit

Architectures and Performance Requirements for Genetic Circuit Promoters. For a genetic circuit to reliably measure the ability of an engineered protein to repress transcription, expression of this repressor protein must be tightly regulated such that differences between maximum and minimum output signals are reproducible, significant, and directly dependent on the controllable expression and function of the experimental repressor. This requires that the promoter controlling expression of the engineered repressor (pDBD) and the regulatory component that binds this promoter (LacI) be tuned to minimize expression from pDBD in the absence of inducer (*i.e.,* leaky expression) while simultaneously maximizing output expression upon induction, thereby increasing the dynamic range between off (*i.e.,* absence of inducer) and on (*i.e.,* presence of inducer) states. Maximum pDBD expression levels for the experimental repressor (scDBD) should ideally saturate the promoter (pGFP) responsible for expression of the reporting gene (eGFP) [1]. Accordingly, a *three*-component genetic circuit was designed (Fig 3A) with promoters driving scDBD (Cloning Site I: pDBD) and eGFP (Cloning Site II: pGFP) expression incorporating two copies of the $lacO^{sym}$ and $lacO^{tta}$ operator sequence, respectively [2]. These $lacO^{sym}$ and $lacO^{tta}$ operators were inserted at core and proximal positions relative to the RNA polymerase recruitment site (Fig 3B) to maximize repressor occupancy and reduce leaky expression [3, 4].

Reporting Strategy for Quantifying Genetic Circuit Outputs. To evaluate the minimum and maximum expression output from Cloning Site I we constructed a genetic circuit outfitting pDBD with a functional copy of eGFP while simultaneously pairing the pGFP promoter of Cloning Site II with a decoy reporting protein (called dGFP) encoded by a copy of eGFP with the point-mutation R96A [5]. The dGFP gene product exhibits delayed maturation of the chromophore ($t_{½} \approx$ 3 months) resulting in the production of a gene which is expected to comparably burden the host organism without producing a quantifiable green fluorescence signal [6]. The use of dGFP in this genetic circuit setup, annotated in S5 Fig, allowed us to employ a reporting strategy quantifying output signal from Cloning Site I while accounting for metabolic burden imposed by protein expression from Cloning Site II. The output signal (*F*) for any given genetic circuit is quantified as the culture fluorescence (*GFP*, λ_{ex} = 485 nm, λ_{em} = 510 nm, gain = medium, 30 flashes per read), normalized by culture density, as measured by optical density (*OD*, λ_{OD} = 600 nm), (Equation S1).

$$F = \frac{GFP}{OD} \qquad \text{Equation S1}$$

Overview of Experiments Used to Tune Expression Performance of pDBD by Evaluating Cloning Site III Regulatory Elements. In order to tune expression of the engineered repressor such that leaky expression is minimized and induced expression is maximized, LacI under the control of its weak constitutive promoter pLacI was incorporated into Cloning Site III of the pDBD(eGFP)·pGFP(dGFP) genetic circuit. In addition to wild-type LacI, we also tested the W220F single-mutant variant ($LacI_{W220F}$) since it had been demonstrated to improve dynamic range of expression [7]. Either wild-type or W220F mutant LacI was paired with the weak constitutive pLacI promoter or its variant $pLacI^{Q}$ (C/T mutation to −35 promoter box) that had previously been shown to increase constitutive expression of LacI by *ten*-fold [8]. Fluorescence output and culture densities were monitored over a 6- to 9-hour time-course grown in the presence of various inducer concentrations (1 μM to 25 mM IPTG). The experiment was performed across the combinatorial set of *four* LacI promoter-repressor pairs inserted into Cloning Site III, giving pDBD expression results for genetic circuits reported in:

S4 Table & S6 Fig: pDBD(eGFP)·pGFP(dGFP)·pLacI(LacI),
S5 Table & S7 Fig: pDBD(eGFP)·pGFP(dGFP)·$pLacI^{Q}$(LacI),
S6 Table & S8 Fig: pDBD(eGFP)·pGFP(dGFP)·pLacI($LacI_{W220F}$), and
S7 Table & S9 Fig: pDBD(eGFP)·pGFP(dGFP)·$pLacI^{Q}$($LacI_{W220F}$).

The following sections detail how the results from these experiments were used to optimize dynamic range of expression from Cloning Site I, LacI expression levels from Cloning Site III, IPTG inducer concentrations required for maximal expression from Cloning Site I and to identify steady-state growth conditions needed to ensure the collection of reproducible results.

Tuning Dynamic Range of Expression from pDBD. To identify the LacI repressor variant that gives the best dynamic range of expression from Cloning Site I, density-normalized fluorescence was measured for cultures transformed with the pDBD(eGFP)·pGFP(dGFP) circuit containing either LacI or $LacI_{W220F}$ repressors, each constitutively expressed with the same pLacI promoter. As shown in S6D–S6F Figs and S8D–S8F Figs (data in S4 and S6 Tables, respectively) at non-inducing concentrations of IPTG (*i.e.,* < 10 μM), density-normalized fluorescence was very low across all time-points tested (*i.e.,* 7, 8 and 9-hours).

There was no statistically significant difference between normalized fluorescence values between the circuits containing wild-type and W220F mutant LacI repressors. For example, at 1 μM IPTG normalized fluorescence was measured at 363 ± 5 and 368 ± 3, for wild type and W220F LacI circuits at the 8-hour time-point, illustrating that there is no significant difference (*p*-value = 0.343, two-tailed homoscedastic *t*-test) in baseline expression output between the two constructs. In contrast, a significant difference in normalized fluorescence output (*i.e., p*-values < 0.001, two-tailed homoscedastic *t*-test) was observed when inducing concentrations of IPTG were used with the W220F mutant giving rise to a larger dynamic range. For example, at the 8-hour time-point, cultures grown in the presence of 10 mM IPTG yielded 5,827 ± 99 (wild-type LacI) and 7,681 ± 138 (W220F LacI) normalized fluorescence units, resulting in a 16.1 ± 0.4 and 20.9 ± 0.4 fold gain over their baseline expression output (1 μM IPTG), respectively.

Dynamic Range Conclusion: These results demonstrate that while no discernable difference can be observed for basal expression output between pLacI(LacI) and pLacI($LacI_{W220F}$) regulatory elements, that incorporation of pLacI($LacI_{W220F}$) into Cloning Site III of the genetic circuit does improve the dynamic range of expression from the pDBD promoter.

Tuning Repressor Saturation of pDBD. The pDBD promoter architecture incorporates two copies of the $lacO^{sym}$ operator, which raised the possibility that the levels of LacI expression under its native constitutive promoter may not be sufficient to saturate the pDBD promoter. Therefore, we evaluated the utility of incorporating the stronger constitutive promoter $pLacI^Q$ into Cloning Site III as the approximately 10-fold greater cellular concentrations of LacI may help to further reduce baseline expression. As shown in panels D through F in S6−S9 Figs, baseline expression in the presence of non-inducing concentrations of IPTG (<10 μM IPTG) was the same for all pLacI promoter and LacI repressor combinations. For example, at the 8-hour time-point output from the pDBD promoter under repression by $pLacI^Q$(LacI) and $pLacI^Q$($LacI_{W220F}$) was 375 ± 6 and 380 ± 6 density-normalized fluorescence units, respectively. These values were not significantly different relative to baseline expression output achieved using regulatory repressor expressed with pLacI (*p*-values = 0.022 and 0.012 for two-tailed homoscedastic *t*-tests, respectively). However, growth in the presence of inducing concentrations of IPTG (*e.g.,* >1 mM) showed significantly reduced normalized fluorescence for both $pLacI^Q$ constructs compared to their counterparts with pLacI. For example, induction of $pLacI^Q$(LacI) and $pLacI^Q$($LacI_{W220F}$) genetic circuits with 10 mM IPTG produced 506 ± 7 and 613 ± 6 density-normalized fluorescence units, having only 1.35 ± 0.03 and 1.66 ± 0.03 fold gain over baseline expression output (1 μM IPTG), respectively. This reduction in pDBD output expression upon induction may be attributed to the increased cellular concentration of LacI that may exceed accumulated intracellular concentrations of IPTG.

Repressor Saturation Conclusion: Together, these results suggest that the incorporation of pLacI($LacI_{W220F}$) into the genetic circuit at Cloning Site III achieves cellular repressor concentrations sufficient to saturate the pDBD promoter without curtailing pDBD expression output upon induction.

Tuning Inducer Concentration for pDBD Repression and Induction. Because the host organism for these experiments includes endogenous and interacting LacI-type regulatory and regulated elements, it is critical to ensure that experiments are conducted with inducer concentrations that will allow reproducible measurement of reporter gene expression output. The expression output of pDBD from the four genetic circuits outfitted with the combinatorial set of LacI and $LacI_{W220F}$ repressors and pLacI and $pLacI^Q$ promoters was recorded under an inducer series spanning 1 μM to 25 mM concentrations of IPTG. Evaluation of the inducer series at 6-, 7-, 8-, and 9-hour time-points required that the density-normalized fluorescence output (Equation S1) from the genetic circuit (*y*-axis: *F*) be plotted against the IPTG concentration transformed into a unitless negative base-10 logarithm (*x*-axis: x) as described in Equation S2.

$$x = -\log_{10}[\text{IPTG}] \qquad \text{Equation S2}$$

Inducer expression curves were fit to a generalized logistic function representing a sigmoid function ($curve$) adapted with a linear function ($line$) manipulating the maximum asymptote (Equation S3).

$$\mathrm{F}_x = \min_{x \to \infty} \mathrm{F}_x + \frac{\max_{x \to 0} \mathrm{F}_x - \min_{x \to \infty} \mathrm{F}_x}{curve} + line \qquad \text{Equation S3}$$

The sigmoid function ($curve$) has the form represented in Equation S4 adjusting the growth rate (k) of the curve toward the maximum asymptote (sym). Because this function is asymmetric, the midpoint of the curve (mid) must be calculated adjusting the midpoint (k_D) of an unmodified symmetric sigmoid function, as shown in Equation S5.

$$curve = \left[1 + \ 10^{-k(mid-x)}\right]^{sym}$$ Equation S4

$$mid = \ k_{\mathrm{D}} + \left[\frac{1}{-k} \times \log_{10}\left(2^{1/-sym} - 1\right)\right]$$ Equation S5

The linear adjustment of the maximum asymptote of the sigmoid curve is achieved using the function in Equation S6 (omitted for genetic circuits incorporating the pLacI^Q promoter). This function applies the linear correction to the maximum asymptote based on a reference point (ref, Equation S7) suitably distinguished from the midpoint as calculated in Equation S5.

$$line = \frac{slope \times (ref - x)}{1 + 10^{-(ref - x)}}$$ Equation S6

$$ref = \ k_{\mathrm{D}} + \left[\frac{1}{-k} \times \log_{10}\left(10^{6/-sym} - 1\right)\right]$$ Equation S7

Sigmoid curves were fit to experimental data by non-linear regression with error on parameters estimated by bootstrap analysis (S8 Table). Bootstrapping was conducted by randomly applying observed error on experimentally collected data to generate 300 independent resampled data sets. Non-linear regression across the resampled data sets provided a set of parameter estimates from which average and standard deviations were computed. Non-linear regression analysis for expression output as a function of the inducer binding series is demonstrated to have strong agreement with the sigmoidal function (Equation S4) for all time-points and genetic circuits ($0.9581 \leq R^2 \leq 0.9998$) with the exception of the 6-hour time-point for the pDBD(eGFP)·pGFP(dGFP)·pLacI^Q(LacI) genetic circuit (R^2 = 0.868) as indicated by their coefficients of determination (R^2). The midpoint for the sigmoid function fitted to the 8-hour time-point inducer series was identified at 53.7 ± 0.8 and 263 ± 3 μM IPTG for pLacI(LacI) and pLacI(LacI$_{W220F}$) genetic circuits, respectively. Importantly, growth with inducer concentrations in excess of 25 mM IPTG exert considerable metabolic burden on the host supressing culture densities consistent with previously published results [6].

> **Inducer Concentration Conclusion:** These results indicate that the pDBD promoter in genetic circuits incorporating pLacI(LacI) and pLacI(LacI$_{W220F}$) regulatory cassettes at Cloning Site III is reliably repressed (*i.e.,* off-state) in growth media with IPTG concentrations below 10 μM and expressed (*i.e.,* on-state) with IPTG concentrations greater than 1 mM.

Demonstrating Steady-state Behaviour for Genetic Circuit Outputs. Because genetic circuit output is measured as a function of the density-normalized fluorescence (Equation S1) it is imperative that growth conditions and sampling be restricted to steady-state regimes to obtain reproducible measurements. For this reason, the culture density, approximated by optical density (*OD*), and fluorescence output (GFP) were plotted as a function of time using samples taken at 6-, 7-, 8-, and 9-hour time-points for each genetic circuit (S6-S9 Figs, panels B and C). All of the pDBD(eGFP)·pGFP(dGFP) genetic circuits exhibited linear relationships in these plots, with R^2 correlating optical density as a function of time calculated between 0.952 and 1.000, and R^2 correlating fluorescence as a function of time between 0.805 and 0.999. These results confirm that all cultures were in the steady-state phase of growth between the 6 to 9-hour time-points, and would provide quantitatively consistent expression outputs as is required for reproducible measurement of scDBD function.

Summary of Results for Optimization of Cloning Site III cassette: These results demonstrate that the pLacI(LacI$_{W220F}$) regulatory cassette incorporated into Cloning Site III was the best option for tuning pDBD expression, because the genetic circuit pDBD(eGFP)·pGFP(dGFP)·pLacI(LacI$_{W220F}$) had:

1. negligible baseline (1 μM IPTG) expression output (<400 density-normalized culture fluorescence units),
2. the greatest dynamic range (20.9 ± 0.4 fold gain in induced expression output with 10 mM IPTG),
3. saturation of repressor recruitment to pDBD (since basal output did not decrease with use of the pLacI^Q promoter),
4. established minimum (IPTG ≤ 25 μM) and maximum (IPTG ≥ 5 mM) pDBD promoter activity as a function of inducer concentration, and
5. steady-state behaviour between 6- and 9-hour time-points.

The annotated sequence map for this genetic circuit pDBD(eGFP)·pGFP(dGFP)·pLacI(LacIW220F) is shown in S5 Fig.

Annotated plasmid sequence for the control genetic circuit: pDBD(eGFP)·pGFP(dGFP)·pLacI($LacI_{W220F}$)

1	ACATTTCCCC	GAAAAGTGCC	ACCTGACGGT	AGTTTATCAC	AGTTAAATTG	CTAACGCAGT	CAGGCACCGT	GTATGAAATC	TAACAATGCG	CCTGGATGCT
101	GTAGGCATAG	GCTTGGTTAT	GCCGGTACTG	CCGGGCCTCT	TGCGGGATAT	CCGGATATAG	TTCCTCCTTT	CAGCAAAAAA	CCCCTCAAGA	CCCGTTTAGA
201	GGCCCCAAGG	GGTTATGCTA	GTTATTGCTC	AGCGGTGGCA	GCAGCCAACT	CAGCTTCCTT	TCGGGCTTTG	TTAGCAGCCG	GATCCCTATC	ATTATTTGTA
301	GAGTTCATCC	ATACCAAGGG	TAATACCAGC	AGCAGTAACA	AATTCTAACA	AGACCATGTG	GTCTCTCTTT	TCGTTTGGAT	CTTTGGATAA	GGCAGATTGA
401	GTGGATAAGT	AATGGTTGTC	TGGTAACAAG	ACTGGACCAT	CACCAATTGG	AGTATTTTGT	TGATAATGGT	CAGCTAATTG	AACAGAACCA	TCTTCAATGT
501	TGTGTCTAAT	TTTGAAGTTA	ACTTTGATAC	CATTCTTTTG	TTTGTCAGCC	ATGATGTAAA	CATTGTGAGA	GTTATAGTTG	TATTCCAATT	TGTGACCTAA
601	AATGTTACCA	TCTTCTTTAA	AATCAATACC	TTTTAATTCG	ATTCTATTAA	CTAAGGTATC	ACCTTCAAAC	TTGACTTCAG	CTCTGGTCTT	GTAGTTACCG
701	TCATCTTTGA	AAAAAATAGT	TCTTTCTTGA	ACATAACCTT	CTGGCATGGC	AGACTTGAAA	AAGTCATGTT	GTTTCATGTG	ATCTGGGTAT	CTAGAAAAAC
801	ATTGAACACC	ATAAGTCAAA	GTAGTGACTA	AGGTTGGCCA	TGGAACTGGC	AATTTACCAG	TAGTACAAAT	AAATTTTAAG	GTCAATTTAC	CGTACGTAGC
901	ATCACCTTCA	CCTTCACCGG	AGACAGAAAA	TTTGTGACCA	TTAACATCAC	CATCTAATTC	AACCAAAATT	GGGACAACAC	CAGTGAATAA	TTCTTCACCT
1,001	TTAGACATTT	TTTTCCTCCT	TCTAGTTTAA	ACAAAATTAT	TTGTAGAGGC	TGTTTCGTCC	TCACGGACTC	ATCAGACCGG	AAAGCACATC	CGGTGACAGC
1,101	TCGACTCTGC	ACCATAGGA**A**	**ATTGTGAGCG**	**CTCACAATTC**	**CACACAGTAT**	**CTTGTGAGCG**	**CTCACAATTG**	**TCAA**GACAGC	CAAGGTTCGT	TCGCAACAGA
1,201	ACAAGAATGA	TATGTGCCTA	GACCAACCTT	AGCATATGGC	CTCAACCTAC	TACTGGGCTG	CTTCCTAATG	CAGGAGTCGC	ATAAGGGAGA	GCGTCGAGAT
1,301	CCCG**GACACC**	**ATCGAATGGC**	**GCAAAACCTT**	**TCGCGGTATG**	**GCATGATAGC**	**GCCCGGAAGA**	**GAGTCAATTC**	**AGGGTGGTGA**	**AT**GTGAAACC	AGTAACGTTA
1,401	TACGATGTCG	CAGAGTATGC	CGGTGTCTCT	TATCAGACCG	TTTCCCGCGT	GGTGAACCAG	GCCAGCCACG	TTTCTGCGAA	AACGCGGGAA	AAAGTGGAAG
1,501	CGGCGATGGC	GGAGCTGAAT	TACATTCCCA	ACCGCGTGGC	ACAACAACTG	GCGGGCAAAC	AGTCGTTGCT	GATTGGCGTT	GCCACCTCCA	GTCTGGCCCT
1,601	GCACGCGCCG	TCGCAAATTG	TCGCGGCGAT	TAAATCTCGC	GCCGATCAAC	TGGGTGCCAG	CGTGGTGGTG	TCGATGGTAG	AACGAAGCGG	CGTCGAAGCC
1,701	TGTAAAGCGG	CGGTGCACAA	TCTTCTCGCG	CAACGCGTCA	GTGGGCTGAT	CATTAACTAT	CCGCTGGATG	ACCAGGATGC	CATTGCTGTG	GAAGCTGCCT
1,801	GCACTAATGT	TCCGGCGTTA	TTTCTTGATG	TCTCTGACCA	GACACCCATC	AACAGTATTA	TTTTCTCCCA	TGAAGACGGT	ACGCGACTGG	GCGTGGAGCA
1,901	TCTGGTCGCA	TTGGGTCACC	AGCAAATCGC	GCTGTTAGCG	GGCCCATTAA	GTTCTGTCTC	GGCGCGTCTG	CGTCTGGCTG	GCTGGCATAA	ATATCTCACT
2,001	CGCAATCAAA	TTCAGCCGAT	AGCGGAACGG	GAAGGCGACT	TCAGTGCCAT	GTCCGGTTTT	CAACAAACCA	TGCAAATGCT	GAATGAGGGC	ATCGTTCCCA
2,101	CTGCGATGCT	GGTTGCCAAC	GATCAGATGG	CGCTGGGCGC	AATGCGCGCC	ATTACCGAGT	CCGGGCTGCG	CGTTGGTGCG	GATATCTCGG	TAGTGGGATA
2,201	CGACGATACC	GAAGACAGCT	CATGTTATAT	CCCGCCGTTA	ACCACCATCA	AACAGGATTT	TCGCCTGCTG	GGGCAAACCA	GCGTGGACCG	CTTGCTGCAA
2,301	CTCTCTCAGG	GCCAGGCGGT	GAAGGGCAAT	CAGCTGTTGC	CCGTCTCACT	GGTGAAAAGA	AAAACCACCC	TGGCGCCCAA	TACGCAAACC	GCCTCTCCCC
2,401	GCGCGTTGGC	CGATTCATTA	ATGCAGCTGG	CACGACAGGT	TTCCCGACTG	GAAAGCGGGC	AGTGAGCGCA	ACGCAATTAA	TGTAAGTTAG	CTCACTCATT
2,501	AGGCACCGGG	ATCTCGACCG	ATGCCCTTGA	GAGCCTTCAA	CCCAGTCAGC	TCCTTCCGGT	GGGCGCGGGG	CATGACTATC	GTCGCCGCAC	TTATGACTGT
2,601	CTTCTTTATC	ATGCAACTCG	TAGGACAGGT	GCCGGCAGCG	CTCTGGGTCA	TTTTCGGCGA	GGACCGCTTT	CGCTGGAGCG	CGACGATGAT	CGGCCTGTCG
2,701	CTTGCGGTAT	TCGGAATCTT	GCACGCCCTC	GCTCAAGCCT	TCGTCACTGG	TCCCGCCACC	AAACGTTTCG	GCGAGAAGCA	GGCCATTATC	GCCGGCATGG
2,801	CGGCCGACGC	GCTGGGCTAC	GTCTTGCTGG	CGTTCGCGAC	GCGAGGCTGG	ATGGCCTTCC	CCATTATGAT	TCTTCTCGCT	TCCGGCGGCA	TCGGGATGCC
2,901	CGCGTTGCAG	GCCATGCTGT	CCAGGCAGGT	AGATGACGAC	CATCAGGGAC	AGCTTCAAGG	ATCGCTCGCG	GCTCTTACCA	GCCTAACTTC	GATCACTGGA
3,001	CCGCTGATCG	TCACGGCGAT	TTATGCCGCC	TCGGCGAGCA	CATGGAACGG	GTTGGCATGG	ATTGTAGGCG	CCGCCCTATA	CCTTGTCTGC	CTCCCCGCGT
3,101	TGCGTCGCGG	TGCATGGAGC	CGGGCCACCT	CGACCTGAAT	GGAAGCCGGC	GGCACCTCGC	TAACGGATTC	ACCACTCCAA	GAATTGGAGC	CAATCAATTC
3,201	TTGCGGAGAA	CTGTGAATGC	GCAAACCAAC	CCTTGGCAGA	ACATATCCGA	ATTCATAGGC	TTGGTTATGC	CGGTACTGCC	GGGCCTCTTG	CGGGATATCC
3,301	GGATATAGTT	CCTCCTTTCA	GCAAAAAACC	CCTCAAGACC	CGTTTAGAGG	CCCCAAGGGG	TTATGCTAGT	TATTGCTCAG	CGGTGGCAGC	AGCCAACTCA
3,401	GCTTCCTTTC	GGGCTTTGTT	AGCAGCCGCT	AGCCTATCAT	TATTTGTAGA	GTTCATCCAT	ACCAAGGGTA	ATACCAGCAG	CAGTAACAAA	TTCTAACAAG
3,501	ACCATGTGGT	CTCTCTTTTC	GTTTGGATCT	TTGGATAAGG	CAGATTGAGT	GGATAAGTAA	TGGTTGTCTG	GTAACAAGAC	TGGACCATCA	CCAATTGGAG
3,601	TATTTTGTTG	ATAATGGTCA	GCTAATTGAA	CAGAACCATC	TTCAATGTTG	TGTCTAATTT	TGAAGTTAAC	TTTGATACCA	TTCTTTTGTT	TGTCAGCCAT
3,701	GATGTAAACA	TTGTGAGAGT	TATAGTTGTA	TTCCAATTTG	TGACCTAAAA	TGTTACCATC	TTCTTTAAAA	TCAATACCTT	TTAATTCGAT	TCTATTAACT
3,801	AAGGTATCAC	CTTCAAACTT	GACTTCAGCT	CTGGTCTTGT	AGTTACCGTC	ATCTTTGAAA	AAAATAGTTG	CTTCTTGAAC	ATAACCTTCT	GGCATGGCAG
3,901	ACTTGAAAAA	GTCATGTTGT	TTCATGTGAT	CTGGGTATCT	AGAAAAACAT	TGAACACCAT	AAGTCAAAGT	AGTGACTAAG	GTTGGCCATG	GAACTGGCAA
4,001	TTTACCAGTA	GTACAAATAA	ATTTTAAGGT	CAATTTACCG	TACGTAGCAT	CACCTTCACC	TTCACCGGAG	ACAGAAAATT	TGTGACCATT	AACATCACCA
4,101	TCTAATTCAA	CCAAAATTGG	GACAACACCA	GTGAATAATT	CTTCACCTTT	AGACATTTTT	TTCCTCCTTC	TAGTTTAAAC	AAAATTATTT	GTAGAGGCTG
4,201	TTTCGTCCTC	ACGGACTCAT	CAGACCGGAA	AGCACATCCG	GTGACAGCTC	GACTCTGCAC	CATAGGA**AAT**	**TTTAAGCGCT**	**TAAAATTCCA**	**CACAGTTTCT**
4,301	**TTTAAGCGCT**	**TAAAATTGTC**	**AA**GACAGCCA	AGGTTCGTTC	GCAACAGAAC	AAGAATGGTA	TGTGCCTAGA	CCAACCTTTG	CTCGAGTTTC	GTAAAGTCTG
4,401	GAAACGCGGA	AGTCAGCGCC	CTGCACCATT	ATGTTCCGGA	TCTGCATCGC	AGGATGCTGC	TGGCTACCCT	GTGGAACACC	TACATCTGTA	TTAACGAAGC
4,501	GCTGGCATTG	ACCCTGAGTG	ATTTTTCTCT	GGTCCCGCCG	CATCCATACC	GCCAGTTGTT	TACCCTCACA	ACGTTCCAGT	AACCGGGCAT	GTTCATCATC
4,601	AGTAACCCGT	ATCGTGAGCA	TCCTCTCTCG	TTTCATCGGT	ATCATTACCC	CCATGAACAG	AAATCCCCCT	TACACGGAGG	CATCAGTGAC	CAAACAGGAA
4,701	AAAACCGCCC	TTAACATGGC	CCGCTTTATC	AGAAGCCAGA	CATTAACGCT	TCTGGAGAAA	CTCAACGAGC	TGGACGCGGA	TGAACAGGCA	GACATCTGTG
4,801	AATCGCTTCA	CGACCACGCT	GATGAGCTTT	ACCGCAGCTG	CCTCGCGCGT	TTCGGTGATG	ACGGTGAAAA	CCTCTGACAC	ATGCAGCTCC	CGGAGACGGT
4,901	CACAGCTTGT	CTGTAAGCGG	ATGCCGGGAG	CAGACAAGCC	CGTCAGGGCG	CGTCAGCGGG	TGTTGGCGGG	TGTCGGGGCG	CAGCCATGAC	CCAGTCACGT
5,001	AGCGATAGCG	GAGTGTATAC	TGGCTTAACT	ATGCGGCATC	AGAGCAGATT	GTACTGAGAG	TGCACCATAT	ATGCGGTGTG	AAATACCGCA	CAGATGCGTA
5,101	AGGAGAAAAT	ACCGCATCAG	GCGCTCTTCC	GCTTCCTCGC	TCACTGACTC	GCTGCGCTCG	GTCGTTCGGC	TGCGGCGAGC	GGTATCAGCT	CACTCAAAGG
5,201	CGGTAATACG	GTTATCCACA	GAATCAGGGG	ATAACGCAGG	AAAGAACATG	TGAGCAAAAG	GCCAGCAAAA	GGCCAGGAAC	CGTAAAAAGG	CCGCGTTGCT
5,301	GGCGTTTTTC	CATAGGCTCC	GCCCCCCTGA	CGAGCATCAC	AAAAATCGAC	GCTCAAGTCA	GAGGTGGCGA	AACCCGACAG	GACTATAAAG	ATACCAGGCG
5,401	TTTCCCCCTG	GAAGCTCCCT	CGTGCGCTCT	CCTGTTCCGA	CCCTGCCGCT	TACCGGATAC	CTGTCCGCCT	TTCTCCCTTC	GGGAAGCGTG	GCGCTTTCTC
5,501	ATAGCTCACG	CTGTAGGTAT	CTCAGTTCGG	TGTAGGTCGT	TCGCTCCAAG	CTGGGCTGTG	TGCACGAACC	CCCCGTTCAG	CCCGACCGCT	GCGCCTTATC
5,601	CGGTAACTAT	CGTCTTGAGT	CCAACCCGGT	AAGACACGAC	TTATCGCCAC	TGGCAGCAGC	CACTGGTAAC	AGGATTAGCA	GAGCGAGGTA	TGTAGGCGGT
5,701	GCTACAGAGT	TCTTGAAGTG	GTGGCCTAAC	TACGGCTACA	CTAGAAGGAC	AGTATTTGGT	ATCTGCGCTC	TGCTGAAGCC	AGTTACCTTC	GGAAAAAGAG
5,801	TTGGTAGCTC	TTGATCCGGC	AAACAAACCA	CCGCTGGTAG	CGGTGGTTTT	TTTGTTTGCA	AGCAGCAGAT	TACGCGCAGA	AAAAAAGGAT	CTCAAGAAGA
5,901	TCCTTTGATC	TTTTCTACGG	GGTCTGACGC	TCAGTGGAAC	GAAAACTCAC	GTTAAGGGAT	TTTGGTCATG	AGATTATCAA	AAAGGATCTT	CACCTAGATC
6,001	CTTTTAAATT	AAAAATGAAG	TTTTAAATCA	ATCTAAAGTA	TATATGAGTA	AACTTGGTCT	GACAGTTACC	AATGCTTAAT	CAGTGAGGCA	CCTATCTCAG
6,101	CGATCTGTCT	ATTTCGTTCA	TCCATAGTTG	CCTGACTCCC	CGTCGTGTAG	ATAACTACGA	TACGGGAGGG	CTTACCATCT	GGCCCCAGTG	CTGCAATGAT
6,201	ACCGCGAGAC	CCACGCTCAC	CGGCTCCAGA	TTTATCAGCA	ATAAACCAGC	CAGCCGGAAG	GGCCGAGCGC	AGAAGTGGTC	CTGCAACTTT	ATCCGCCTCC
6,301	ATCCAGTCTA	TTAATTGTTG	CCGGGAAGCT	AGAGTAAGTA	GTTCGCCAGT	TAATAGTTTG	CGCAACGTTG	TTGCCATTGC	TGCAGGCATC	GTGGTGTCAC
6,401	GCTCGTCGTT	TGGTATGGCT	TCATTCAGCT	CCGGTTCCCA	ACGATCAAGG	CGAGTTACAT	GATCCCCCAT	GTTGTGCAAA	AAAGCGGTTA	GCTCCTTCGG
6,501	TCCTCCGATC	GTTGTCAGAA	GTAAGTTGGC	CGCAGTGTTA	TCACTCATGG	TTATGGCAGC	ACTGCATAAT	TCTCTTACTG	TCATGCCATC	CGTAAGATGC
6,601	TTTTCTGTGA	CTGGTGAGTA	CTCAACCAAG	TCATTCTGAG	AATAGTGTAT	GCGGCGACCG	AGTTGCTCTT	GCCCGGCGTC	AACACGGGAT	AATACCGCGC
6,701	CACATAGCAG	AACTTTAAAA	GTGCTCATCA	TTGGAAAACG	TTCTTCGGGG	CGAAAACTCT	CAAGGATCTT	ACCGCTGTTG	AGATCCAGTT	CGATGTAACC
6,801	CACTCGTGCA	CCCAACTGAT	CTTCAGCATC	TTTTACTTTC	ACCAGCGTTT	CTGGGTGAGC	AAAAACAGGA	AGGCAAAATG	CCGCAAAAAA	GGGAATAAGG
6,901	GCGACACGGA	AATGTTGAAT	ACTCAT**ACTC**	**TTCCTTTTTC**	**AATATTATTG**	**AAGCATTTAT**	**CAGGGTTATT**	**GTCTCATGAG**	**CGGATACATA**	**TTTGAATGTA**
7,001	**TTTAGAAAAA**	**TAAACAAATA**	**GGGGTTCCGC**	GC						

S5 Fig. Annotated plasmid sequence encoding the three-component genetic circuit. Plasmid sequence for the control genetic circuit pDBD(eGFP)·pGFP(dGFP)·pLacI($LacI_{W220F}$). Annotation indicates the genetic components encoding cloning Site I (249 – 1,232 bp, purple), cloning Site II (3,297 – 4,380 bp, blue), and cloning Site III (1,305 – 2,465 bp, orange), whose sequence identities have all been confirmed by Sanger sequencing. Promoter sequences for these cloning sites are indicated in bold while the DNA sequences encoding the genes eGFP (Site I), dGFP (Site II), and $LacI_{W220F}$ (Site III) are underlined. The restriction sites unique to cloning Site I, BamHI (280 – 285 bp) and NdeI (1,233 – 1,238 bp), and Site II, NheI (3,428 – 3433 bp) and XhoI (4,381 – 4,386 bp), are indicated in red. Annotated plasmid sequence includes the origin of replication (5,307 – 5,895 bp, gold) and selection marker (6,066 – 7,031 bp, green with gene and promoter sequence indicated by underline and bold fonts, respectively).

S4 Table. Data and regression analysis for the genetic circuit pDBD(eGFP)·pGFP(dGFP)·pLacI(LacI)

IPTG (M)	Time-Point Data[a]				Linear Regression Analysis		
	6 Hours	7 Hours	8 Hours	9 Hours	R^2	Slope	Intercept
**Optical Density** $\times 10^{-1}$ (λ_{OD} = 600 nm)[b]							
1.0×10^{-6}	3.33 ± 0.06	4.89 ± 0.08	6.19 ± 0.08	7.10 ± 0.13	0.986	1.26 ± 0.10	−4.09 ± 0.79
1.0×10^{-5}	3.30 ± 0.11	4.94 ± 0.19	6.30 ± 0.08	7.15 ± 0.14	0.982	1.29 ± 0.13	−4.26 ± 0.95
2.5×10^{-5}	3.27 ± 0.16	4.92 ± 0.23	6.36 ± 0.30	7.19 ± 0.28	0.981	1.32 ± 0.13	−4.47 ± 0.99
5.0×10^{-5}	3.21 ± 0.10	4.86 ± 0.17	6.26 ± 0.11	7.08 ± 0.06	0.979	1.30 ± 0.14	−4.41 ± 1.03
1.0×10^{-4}	3.12 ± 0.12	4.77 ± 0.20	6.19 ± 0.13	7.02 ± 0.14	0.980	1.31 ± 0.13	−4.56 ± 1.00
2.5×10^{-4}	3.03 ± 0.12	4.62 ± 0.21	6.04 ± 0.14	6.96 ± 0.08	0.987	1.32 ± 0.11	−4.76 ± 0.82
5.0×10^{-4}	3.02 ± 0.15	4.59 ± 0.24	6.06 ± 0.15	6.94 ± 0.10	0.985	1.32 ± 0.11	−4.78 ± 0.87
1.0×10^{-3}	3.02 ± 0.17	4.60 ± 0.29	6.03 ± 0.18	6.93 ± 0.13	0.986	1.31 ± 0.11	−4.72 ± 0.84
2.5×10^{-3}	3.00 ± 0.19	4.52 ± 0.28	5.88 ± 0.23	6.63 ± 0.12	0.980	1.22 ± 0.12	−4.17 ± 0.94
5.0×10^{-3}	3.05 ± 0.19	4.60 ± 0.32	5.87 ± 0.20	6.54 ± 0.14	0.972	1.18 ± 0.14	−3.80 ± 1.07
1.0×10^{-2}	3.04 ± 0.13	4.68 ± 0.30	5.83 ± 0.21	6.52 ± 0.22	0.968	1.16 ± 0.15	−3.67 ± 1.13
2.5×10^{-2}	3.17 ± 0.10	4.74 ± 0.20	5.67 ± 0.19	6.49 ± 0.07	0.975	1.09 ± 0.12	−3.16 ± 0.94
**Fluorescence** $\times 10^{3}$ (λ_{ex} = 485 nm, λ_{em} = 510 nm)[c]							
1.0×10^{-6}	0.194 ± 0.002	0.211 ± 0.002	0.225 ± 0.004	0.236 ± 0.004	0.990	0.014 ± 0.001	0.112 ± 0.007
1.0×10^{-5}	0.203 ± 0.004	0.222 ± 0.006	0.250 ± 0.003	0.302 ± 0.006	0.951	0.033 ± 0.005	0.000 ± 0.040
2.5×10^{-5}	0.209 ± 0.011	0.240 ± 0.010	0.403 ± 0.015	0.749 ± 0.027	0.864	0.178 ± 0.050	−0.938 ± 0.380
5.0×10^{-5}	0.279 ± 0.010	0.658 ± 0.025	1.591 ± 0.032	2.483 ± 0.021	0.971	0.754 ± 0.092	−4.405 ± 0.694
1.0×10^{-4}	0.682 ± 0.059	1.523 ± 0.102	2.722 ± 0.110	3.660 ± 0.124	0.996	1.013 ± 0.046	−5.453 ± 0.352
2.5×10^{-4}	1.035 ± 0.055	1.913 ± 0.078	3.125 ± 0.068	4.125 ± 0.041	0.997	1.048 ± 0.043	−5.311 ± 0.327
5.0×10^{-4}	1.110 ± 0.089	1.984 ± 0.121	3.206 ± 0.124	4.192 ± 0.085	0.996	1.047 ± 0.045	−5.229 ± 0.340
1.0×10^{-3}	1.184 ± 0.100	2.090 ± 0.151	3.323 ± 0.143	4.351 ± 0.102	0.997	1.073 ± 0.042	−5.312 ± 0.321
2.5×10^{-3}	1.209 ± 0.118	2.075 ± 0.173	3.266 ± 0.204	4.171 ± 0.147	0.996	1.008 ± 0.044	−4.878 ± 0.332
5.0×10^{-3}	1.288 ± 0.112	2.205 ± 0.179	3.408 ± 0.141	4.251 ± 0.101	0.996	1.009 ± 0.047	−4.781 ± 0.357
1.0×10^{-2}	1.310 ± 0.074	2.262 ± 0.168	3.395 ± 0.159	4.196 ± 0.130	0.996	0.979 ± 0.043	−4.554 ± 0.329
2.5×10^{-2}	1.459 ± 0.035	2.402 ± 0.112	3.458 ± 0.106	4.353 ± 0.047	0.999	0.974 ± 0.021	−4.385 ± 0.158
**Normalized Fluorescence** $\times 10^{3}$ (λ_{ex} = 485 nm, λ_{em} = 510 nm, λ_{OD} = 600 nm)[d]							
1.0×10^{-6}	0.584 ± 0.006	0.432 ± 0.006	0.363 ± 0.005	0.332 ± 0.001	0.999	0.110 ± 0.002	0.158 ± 0.001
1.0×10^{-5}	0.614 ± 0.011	0.450 ± 0.010	0.397 ± 0.004	0.422 ± 0.002	0.876	0.240 ± 0.064	0.114 ± 0.036
2.5×10^{-5}	0.638 ± 0.009	0.488 ± 0.006	0.634 ± 0.036	1.043 ± 0.065	0.756	1.252 ± 0.502	−0.280 ± 0.283
5.0×10^{-5}	0.872 ± 0.016	1.353 ± 0.043	2.541 ± 0.056	3.509 ± 0.059	0.913	5.560 ± 1.214	−1.723 ± 0.674
1.0×10^{-4}	2.182 ± 0.108	3.190 ± 0.092	4.398 ± 0.090	5.212 ± 0.078	0.974	7.564 ± 0.880	−1.843 ± 0.482
2.5×10^{-4}	3.414 ± 0.049	4.145 ± 0.031	5.170 ± 0.031	5.924 ± 0.058	0.980	7.807 ± 0.781	−1.482 ± 0.420
5.0×10^{-4}	3.674 ± 0.111	4.325 ± 0.042	5.292 ± 0.084	6.043 ± 0.050	0.980	7.790 ± 0.780	−1.388 ± 0.418
1.0×10^{-3}	3.912 ± 0.117	4.542 ± 0.055	5.508 ± 0.085	6.278 ± 0.050	0.980	8.036 ± 0.809	−1.399 ± 0.433
2.5×10^{-3}	4.019 ± 0.142	4.587 ± 0.102	5.554 ± 0.135	6.289 ± 0.109	0.978	8.075 ± 0.854	−1.363 ± 0.443
5.0×10^{-3}	4.223 ± 0.104	4.788 ± 0.063	5.806 ± 0.062	6.496 ± 0.044	0.976	8.382 ± 0.923	−1.416 ± 0.479
1.0×10^{-2}	4.305 ± 0.075	4.833 ± 0.053	5.827 ± 0.099	6.440 ± 0.078	0.976	8.238 ± 0.918	−1.341 ± 0.476
2.5×10^{-2}	4.606 ± 0.068	5.064 ± 0.076	6.105 ± 0.089	6.703 ± 0.085	0.974	8.711 ± 1.001	−1.453 ± 0.517

[a]data reported as the mean (μ) ± standard deviation (σ) from four (n = 4) measurements at each time-point
[b]linear regression analysis performed for optical density (*y*-axis) as a function of time (*x*-axis) plotted in S6B Fig
[c]linear regression analysis performed for fluorescence (*y*-axis) as a function of time (*x*-axis) plotted in S6C Fig
[d]linear regression analysis performed for fluorescence (*y*-axis) as a function of optical density (*x*-axis)

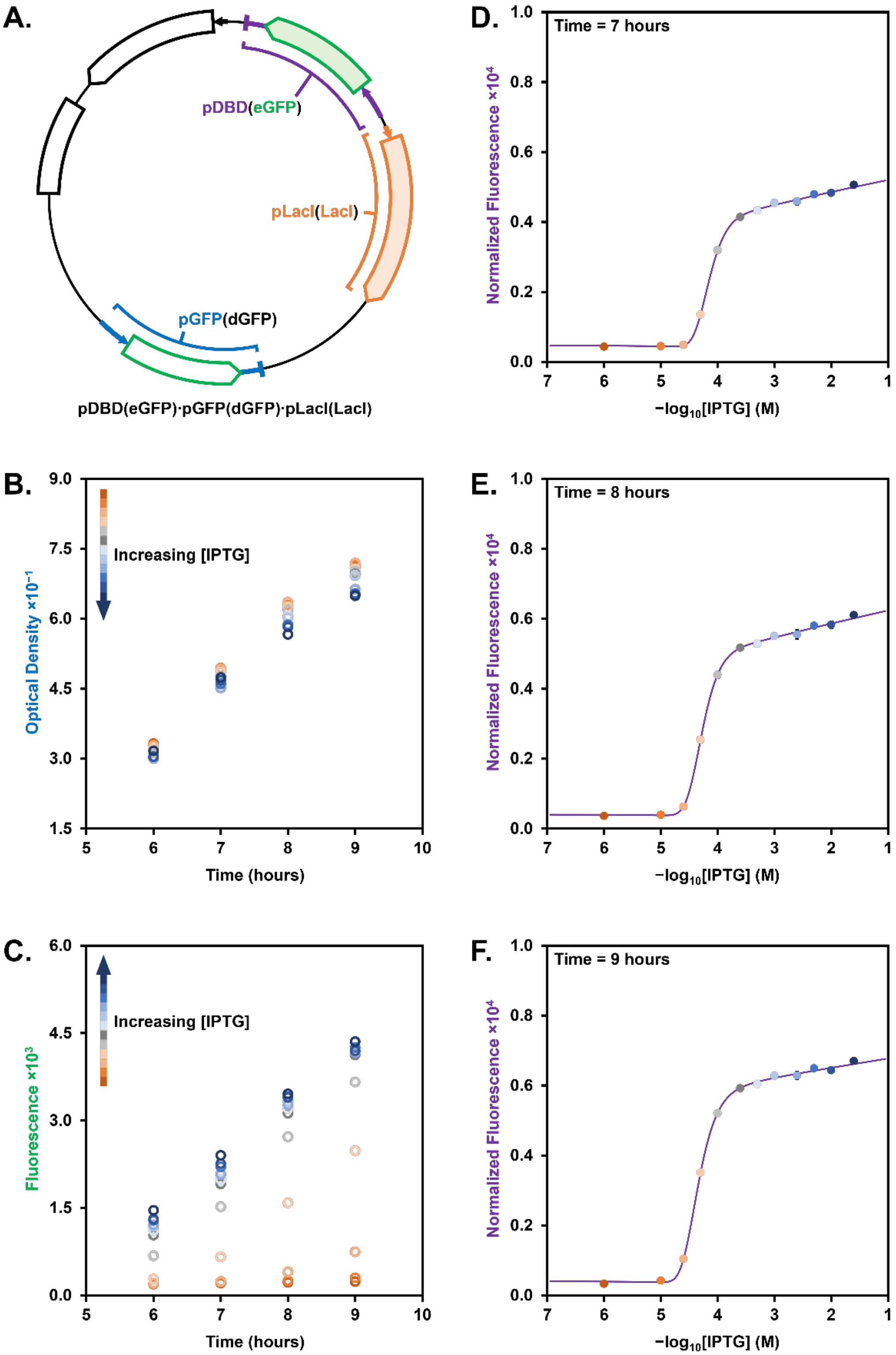

S6 Fig. Inducer series plots for the genetic circuit pDBD(eGFP)·pGFP(dGFP)·pLacI(LacI). Data includes the plasmid map for the genetic circuit (A), plot of optical density at 600 nm (B), and culture fluorescence at 510 nm (λ_{ex} = 485 nm) (C), as a function of growth time over the range of IPTG concentrations indicated by the colour scheme, and plots of normalized fluorescence as a function of inducer concentration for 7- (D), 8- (E), and 9- (F) hour time-points.

S5 Table. Data and regression analysis for the genetic circuit pDBD(eGFP)·pGFP(dGFP)·pLacI^{Q}(LacI)

IPTG (M)	Time-Point Data[a]				Linear Regression Analysis		
	6 Hours	7 Hours	8 Hours	9 Hours	R^2	Slope	Intercept
**Optical Density ×10^{-1} (λ_{OD} = 600 nm)[b]**							
1.0×10^{-6}	2.96 ± 0.11	4.59 ± 0.19	5.87 ± 0.19	6.95 ± 0.08	0.992	1.33 ± 0.09	−4.85 ± 0.65
1.0×10^{-5}	2.89 ± 0.16	4.46 ± 0.23	5.85 ± 0.24	6.92 ± 0.13	0.993	1.35 ± 0.08	−5.08 ± 0.59
2.5×10^{-5}	2.83 ± 0.16	4.38 ± 0.22	5.82 ± 0.27	6.90 ± 0.11	0.994	1.36 ± 0.08	−5.24 ± 0.58
5.0×10^{-5}	2.76 ± 0.16	4.28 ± 0.20	5.79 ± 0.26	6.89 ± 0.19	0.994	1.39 ± 0.07	−5.48 ± 0.55
1.0×10^{-4}	2.68 ± 0.12	4.21 ± 0.13	5.75 ± 0.20	6.90 ± 0.07	0.996	1.42 ± 0.07	−5.77 ± 0.50
2.5×10^{-4}	2.64 ± 0.10	4.21 ± 0.21	5.73 ± 0.20	6.74 ± 0.12	0.991	1.38 ± 0.09	−5.54 ± 0.72
5.0×10^{-4}	2.70 ± 0.13	4.20 ± 0.23	5.67 ± 0.24	6.75 ± 0.13	0.995	1.36 ± 0.07	−5.38 ± 0.54
1.0×10^{-3}	2.72 ± 0.12	4.23 ± 0.19	5.75 ± 0.22	6.84 ± 0.13	0.995	1.39 ± 0.07	−5.55 ± 0.55
2.5×10^{-3}	2.57 ± 0.18	4.12 ± 0.16	5.62 ± 0.16	6.77 ± 0.06	0.996	1.41 ± 0.07	−5.81 ± 0.51
5.0×10^{-3}	2.84 ± 0.14	4.20 ± 0.10	5.52 ± 0.12	6.76 ± 0.07	1.000	1.31 ± 0.02	−5.00 ± 0.15
1.0×10^{-2}	2.82 ± 0.07	4.25 ± 0.11	5.45 ± 0.17	6.33 ± 0.10	0.989	1.17 ± 0.09	−4.10 ± 0.66
2.5×10^{-2}	2.81 ± 0.08	4.05 ± 0.07	5.58 ± 0.16	6.43 ± 0.10	0.989	1.24 ± 0.09	−4.58 ± 0.70
**Fluorescence ×10^{2} (λ_{ex} = 485 nm, λ_{em} = 510 nm)[c]**							
1.0×10^{-6}	1.92 ± 0.06	2.03 ± 0.06	2.20 ± 0.05	2.30 ± 0.01	0.987	0.13 ± 0.01	1.13 ± 0.08
1.0×10^{-5}	1.98 ± 0.06	2.07 ± 0.03	2.33 ± 0.06	2.44 ± 0.02	0.961	0.16 ± 0.02	0.99 ± 0.17
2.5×10^{-5}	1.98 ± 0.08	2.10 ± 0.06	2.37 ± 0.06	2.53 ± 0.02	0.977	0.19 ± 0.02	0.80 ± 0.16
5.0×10^{-5}	2.01 ± 0.06	2.14 ± 0.04	2.47 ± 0.07	2.70 ± 0.01	0.979	0.24 ± 0.03	0.52 ± 0.19
1.0×10^{-4}	1.98 ± 0.06	2.15 ± 0.07	2.56 ± 0.09	2.88 ± 0.02	0.979	0.31 ± 0.03	0.07 ± 0.24
2.5×10^{-4}	2.02 ± 0.03	2.29 ± 0.06	2.81 ± 0.08	3.18 ± 0.04	0.986	0.40 ± 0.03	−0.41 ± 0.25
5.0×10^{-4}	2.06 ± 0.07	2.29 ± 0.09	2.80 ± 0.11	3.21 ± 0.05	0.982	0.40 ± 0.04	−0.39 ± 0.29
1.0×10^{-3}	2.09 ± 0.05	2.37 ± 0.05	2.93 ± 0.07	3.34 ± 0.03	0.986	0.43 ± 0.04	−0.54 ± 0.27
2.5×10^{-3}	1.93 ± 0.13	2.27 ± 0.06	2.86 ± 0.08	3.32 ± 0.04	0.990	0.47 ± 0.03	−0.96 ± 0.25
5.0×10^{-3}	2.14 ± 0.04	2.35 ± 0.02	2.86 ± 0.02	3.37 ± 0.03	0.972	0.42 ± 0.05	−0.48 ± 0.38
1.0×10^{-2}	2.04 ± 0.03	2.31 ± 0.04	2.76 ± 0.06	3.14 ± 0.05	0.991	0.37 ± 0.02	−0.24 ± 0.19
2.5×10^{-2}	2.09 ± 0.09	2.27 ± 0.06	2.85 ± 0.04	3.21 ± 0.06	0.965	0.39 ± 0.05	−0.36 ± 0.40
**Normalized Fluorescence ×10^{2} (λ_{ex} = 485 nm, λ_{em} = 510 nm, λ_{OD} = 600 nm)[d]**							
1.0×10^{-6}	6.49 ± 0.08	4.42 ± 0.08	3.75 ± 0.06	3.31 ± 0.04	0.978	0.98 ± 0.10	1.61 ± 0.05
1.0×10^{-5}	6.86 ± 0.20	4.64 ± 0.19	3.98 ± 0.10	3.52 ± 0.09	0.953	1.19 ± 0.19	1.61 ± 0.10
2.5×10^{-5}	7.01 ± 0.18	4.79 ± 0.11	4.08 ± 0.08	3.67 ± 0.05	0.967	1.40 ± 0.18	1.55 ± 0.10
5.0×10^{-5}	7.27 ± 0.27	5.01 ± 0.17	4.27 ± 0.09	3.93 ± 0.11	0.968	1.73 ± 0.22	1.48 ± 0.11
1.0×10^{-4}	7.39 ± 0.14	5.12 ± 0.04	4.45 ± 0.01	4.17 ± 0.01	0.969	2.16 ± 0.28	1.34 ± 0.14
2.5×10^{-4}	7.66 ± 0.19	5.43 ± 0.17	4.90 ± 0.09	4.71 ± 0.07	0.973	2.85 ± 0.34	1.20 ± 0.17
5.0×10^{-4}	7.61 ± 0.13	5.45 ± 0.10	4.94 ± 0.03	4.75 ± 0.03	0.969	2.89 ± 0.37	1.19 ± 0.19
1.0×10^{-3}	7.70 ± 0.25	5.61 ± 0.14	5.09 ± 0.10	4.87 ± 0.06	0.978	3.06 ± 0.32	1.18 ± 0.17
2.5×10^{-3}	7.53 ± 0.07	5.51 ± 0.13	5.08 ± 0.03	4.90 ± 0.03	0.982	3.34 ± 0.32	1.00 ± 0.16
5.0×10^{-3}	7.54 ± 0.27	5.61 ± 0.15	5.18 ± 0.14	4.99 ± 0.09	0.966	3.21 ± 0.43	1.13 ± 0.22
1.0×10^{-2}	7.25 ± 0.08	5.44 ± 0.08	5.06 ± 0.07	4.96 ± 0.03	0.968	3.13 ± 0.40	1.09 ± 0.20
2.5×10^{-2}	7.44 ± 0.23	5.59 ± 0.15	5.10 ± 0.14	5.00 ± 0.08	0.963	3.17 ± 0.44	1.11 ± 0.22

[a]data reported as the mean (μ) ± standard deviation (σ) from four (n = 4) measurements at each time-point
[b]linear regression analysis performed for optical density (*y*-axis) as a function of time (*x*-axis) plotted in S7B Fig
[c]linear regression analysis performed for fluorescence (*y*-axis) as a function of time (*x*-axis) plotted in S7C Fig
[d]linear regression analysis performed for fluorescence (*y*-axis) as a function of optical density (*x*-axis)

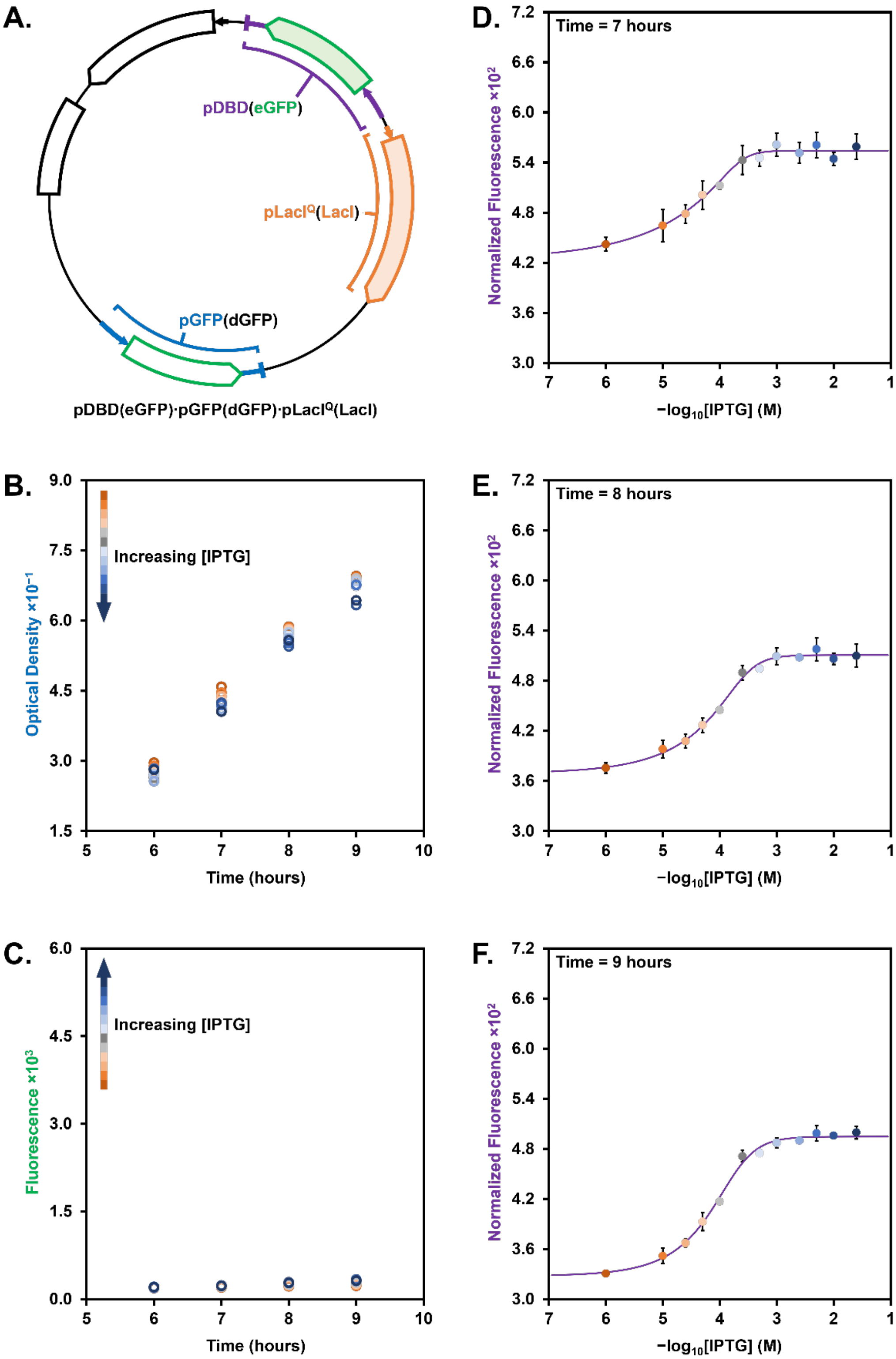

S7 Fig. Inducer series plots for the genetic circuit pDBD(eGFP)·pGFP(dGFP)·pLacI^Q(LacI). Data includes the plasmid map for the genetic circuit (A), plot of optical density at 600 nm (B), and culture fluorescence at 510 nm (λ_{ex} = 485 nm) (C), as a function of growth time over the range of IPTG concentrations indicated by the colour scheme, and plots of normalized fluorescence as a function of inducer concentration for 7- (D), 8- (E), and 9- (F) hour time-points.

S6 Table. Data and regression analysis for the genetic circuit pDBD(eGFP)·pGFP(dGFP)·pLacI(LacI$_{W220F}$)

IPTG (M)	Time-Point Data[a]				Linear Regression Analysis		
	6 Hours	7 Hours	8 Hours	9 Hours	R^2	Slope	Intercept
**Optical Density ×10^{-1} (λ_{OD} = 600 nm)[b]**							
1.0 ×10^{-6}	3.25 ± 0.03	4.79 ± 0.13	5.99 ± 0.18	6.99 ± 0.10	0.991	1.24 ± 0.09	−4.06 ± 0.65
1.0 ×10^{-5}	3.15 ± 0.08	4.68 ± 0.09	6.06 ± 0.09	6.83 ± 0.11	0.981	1.24 ± 0.12	−4.14 ± 0.94
2.5 ×10^{-5}	3.09 ± 0.08	4.68 ± 0.13	6.02 ± 0.16	6.82 ± 0.16	0.980	1.25 ± 0.13	−4.25 ± 0.96
5.0 ×10^{-5}	2.93 ± 0.14	4.46 ± 0.16	5.91 ± 0.17	6.72 ± 0.17	0.983	1.28 ± 0.12	−4.61 ± 0.92
1.0 ×10^{-4}	2.58 ± 0.10	4.04 ± 0.19	5.67 ± 0.14	6.58 ± 0.21	0.988	1.37 ± 0.11	−5.52 ± 0.82
2.5 ×10^{-4}	2.59 ± 0.10	4.01 ± 0.07	5.48 ± 0.18	6.53 ± 0.21	0.995	1.33 ± 0.07	−5.32 ± 0.52
5.0 ×10^{-4}	2.68 ± 0.08	4.03 ± 0.07	5.50 ± 0.13	6.44 ± 0.18	0.992	1.28 ± 0.08	−4.90 ± 0.61
1.0 ×10^{-3}	2.75 ± 0.07	4.08 ± 0.10	5.58 ± 0.12	6.49 ± 0.21	0.991	1.27 ± 0.08	−4.80 ± 0.64
2.5 ×10^{-3}	2.67 ± 0.05	3.91 ± 0.08	4.34 ± 0.80	6.06 ± 0.13	0.952	1.06 ± 0.17	−3.70 ± 1.28
5.0 ×10^{-3}	2.66 ± 0.06	3.89 ± 0.02	5.07 ± 0.12	6.11 ± 0.11	0.998	1.15 ± 0.03	−4.21 ± 0.25
1.0 ×10^{-2}	2.69 ± 0.06	3.96 ± 0.05	4.94 ± 0.08	5.73 ± 0.16	0.989	1.01 ± 0.08	−3.24 ± 0.57
2.5 ×10^{-2}	2.58 ± 0.05	3.76 ± 0.05	5.02 ± 0.07	5.51 ± 0.22	0.970	1.01 ± 0.12	−3.32 ± 0.94
**Fluorescence ×10^{3} (λ_{ex} = 485 nm, λ_{em} = 510 nm)[c]**							
1.0 ×10^{-6}	0.196 ± 0.007	0.204 ± 0.003	0.220 ± 0.008	0.234 ± 0.004	0.985	0.013 ± 0.001	0.118 ± 0.009
1.0 ×10^{-5}	0.199 ± 0.006	0.209 ± 0.005	0.231 ± 0.006	0.241 ± 0.005	0.970	0.015 ± 0.002	0.109 ± 0.014
2.5 ×10^{-5}	0.200 ± 0.004	0.211 ± 0.003	0.235 ± 0.004	0.254 ± 0.007	0.982	0.018 ± 0.002	0.088 ± 0.013
5.0 ×10^{-5}	0.202 ± 0.005	0.211 ± 0.006	0.245 ± 0.007	0.287 ± 0.009	0.936	0.029 ± 0.005	0.018 ± 0.041
1.0 ×10^{-4}	0.191 ± 0.007	0.212 ± 0.004	0.288 ± 0.014	0.564 ± 0.047	0.805	0.120 ± 0.042	−0.583 ± 0.316
2.5 ×10^{-4}	0.232 ± 0.006	0.633 ± 0.020	1.926 ± 0.051	3.540 ± 0.079	0.942	1.122 ± 0.196	−6.830 ± 1.487
5.0 ×10^{-4}	0.618 ± 0.023	1.671 ± 0.054	3.324 ± 0.135	4.867 ± 0.194	0.992	1.440 ± 0.092	−8.179 ± 0.700
1.0 ×10^{-3}	1.040 ± 0.046	2.224 ± 0.082	3.890 ± 0.156	5.375 ± 0.252	0.996	1.467 ± 0.067	−7.871 ± 0.508
2.5 ×10^{-3}	1.204 ± 0.013	2.323 ± 0.054	3.144 ± 0.581	5.156 ± 0.127	0.963	1.268 ± 0.176	−6.552 ± 1.336
5.0 ×10^{-3}	1.301 ± 0.064	2.428 ± 0.123	3.838 ± 0.165	5.336 ± 0.160	0.996	1.351 ± 0.060	−6.910 ± 0.458
1.0 ×10^{-2}	1.431 ± 0.025	2.560 ± 0.053	3.793 ± 0.092	5.038 ± 0.163	0.999	1.205 ± 0.019	−5.834 ± 0.147
2.5 ×10^{-2}	1.436 ± 0.007	2.581 ± 0.036	4.070 ± 0.124	5.075 ± 0.297	0.995	1.241 ± 0.063	−6.014 ± 0.475
**Normalized Fluorescence ×10^{3} (λ_{ex} = 485 nm, λ_{em} = 510 nm, λ_{OD} = 600 nm)[d]**							
1.0 ×10^{-6}	0.604 ± 0.018	0.427 ± 0.010	0.368 ± 0.003	0.334 ± 0.001	0.957	0.101 ± 0.015	0.161 ± 0.008
1.0 ×10^{-5}	0.633 ± 0.021	0.446 ± 0.014	0.382 ± 0.009	0.353 ± 0.002	0.960	0.118 ± 0.017	0.159 ± 0.009
2.5 ×10^{-5}	0.649 ± 0.003	0.452 ± 0.008	0.390 ± 0.005	0.372 ± 0.003	0.941	0.141 ± 0.025	0.152 ± 0.013
5.0 ×10^{-5}	0.689 ± 0.023	0.473 ± 0.015	0.414 ± 0.007	0.427 ± 0.003	0.865	0.216 ± 0.060	0.128 ± 0.031
1.0 ×10^{-4}	0.742 ± 0.020	0.525 ± 0.019	0.508 ± 0.016	0.856 ± 0.050	0.716	0.821 ± 0.366	−0.073 ± 0.182
2.5 ×10^{-4}	0.898 ± 0.026	1.580 ± 0.077	3.515 ± 0.098	5.423 ± 0.102	0.913	8.277 ± 1.811	−2.268 ± 0.885
5.0 ×10^{-4}	2.310 ± 0.096	4.146 ± 0.105	6.041 ± 0.124	7.557 ± 0.116	0.978	11.17 ± 1.181	−2.587 ± 0.576
1.0 ×10^{-3}	3.778 ± 0.118	5.452 ± 0.086	6.973 ± 0.147	8.282 ± 0.138	0.986	11.44 ± 0.970	−2.271 ± 0.478
2.5 ×10^{-3}	4.508 ± 0.054	5.941 ± 0.102	7.253 ± 0.071	8.506 ± 0.035	0.991	11.84 ± 0.810	−2.070 ± 0.358
5.0 ×10^{-3}	4.893 ± 0.137	6.233 ± 0.280	7.566 ± 0.248	8.739 ± 0.137	0.990	11.69 ± 0.837	−1.954 ± 0.386
1.0 ×10^{-2}	5.315 ± 0.136	6.461 ± 0.131	7.681 ± 0.138	8.786 ± 0.145	0.984	11.78 ± 1.067	−1.897 ± 0.478
2.5 ×10^{-2}	5.563 ± 0.087	6.856 ± 0.143	8.104 ± 0.134	9.200 ± 0.180	0.984	12.09 ± 1.100	−1.812 ± 0.481

[a]data reported as the mean (μ) ± standard deviation (σ) from four (n = 4) measurements at each time-point
[b]linear regression analysis performed for optical density (*y*-axis) as a function of time (*x*-axis) plotted in S8B Fig
[c]linear regression analysis performed for fluorescence (*y*-axis) as a function of time (*x*-axis) plotted in S8C Fig
[d]linear regression analysis performed for fluorescence (*y*-axis) as a function of optical density (*x*-axis)

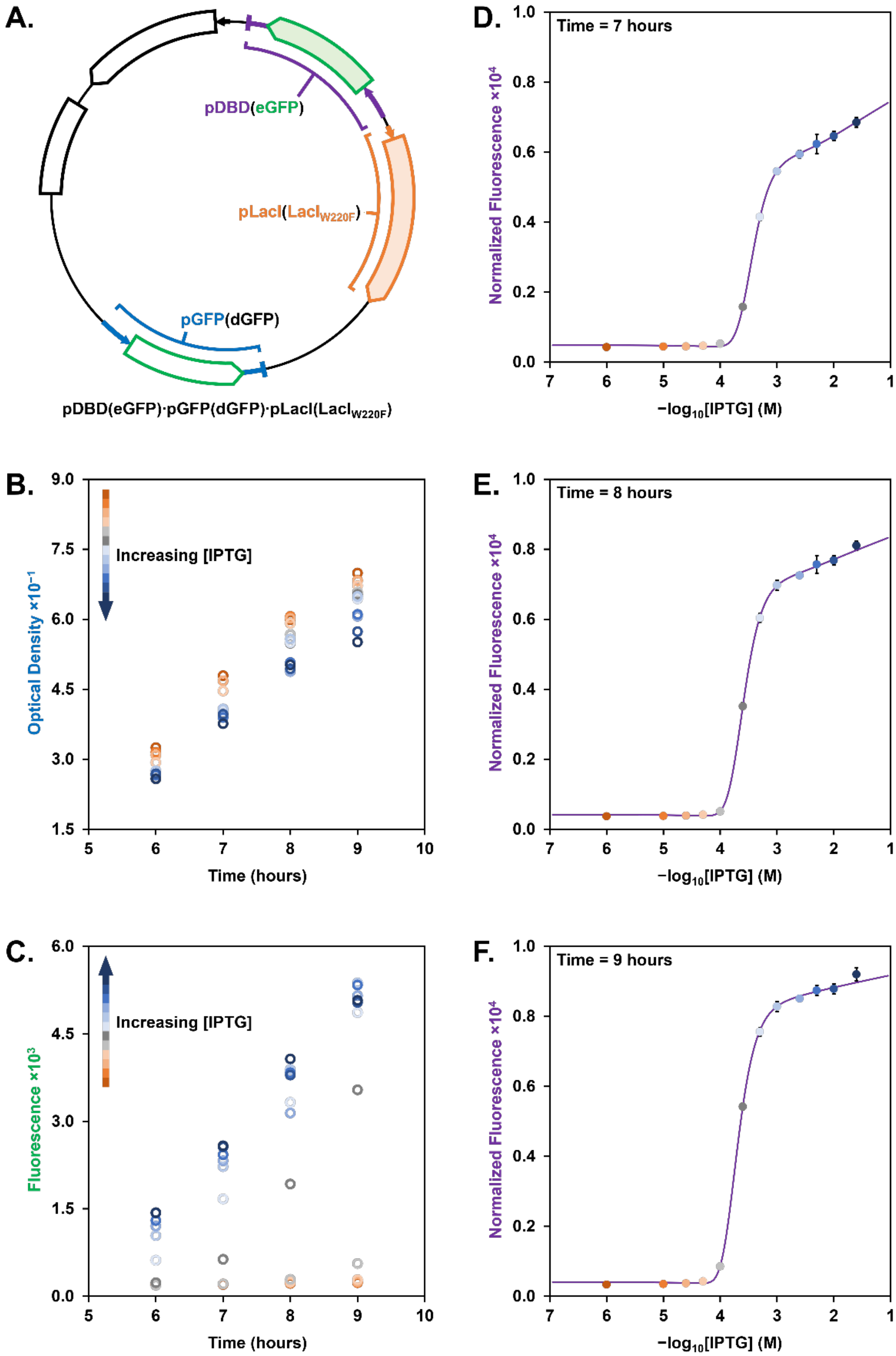

S8 Fig. Inducer series plots for the genetic circuit pDBD(eGFP)·pGFP(dGFP)·pLacI(LacI$_{W220F}$). Data includes the plasmid map for the genetic circuit (A), plot of optical density at 600 nm (B), and culture fluorescence at 510 nm (λ_{ex} = 485 nm) (C), as a function of growth time over the range of IPTG concentrations indicated by the colour scheme, and plots of normalized fluorescence as a function of inducer concentration for 7- (D), 8- (E), and 9- (F) hour time-points.

S7 Table. Data and regression analysis for the genetic circuit pDBD(eGFP)·pGFP(dGFP)·pLacI^Q(LacI$_{W220F}$)

IPTG (M)	Time-point Data[a]				Linear Regression Analysis		
	6 Hours	7 Hours	8 Hours	9 Hours	R^2	Slope	Intercept
**Optica Density ×10^{-1} (λ_{OD} = 600 nm)[b]**							
1.0 ×10^{-6}	2.81 ± 0.08	4.13 ± 0.07	5.68 ± 0.04	6.48 ± 0.11	0.986	1.26 ± 0.11	−4.66 ± 0.81
1.0 ×10^{-5}	2.74 ± 0.03	4.11 ± 0.07	5.65 ± 0.08	6.35 ± 0.08	0.979	1.24 ± 0.13	−4.59 ± 0.97
2.5 ×10^{-5}	2.67 ± 0.02	4.07 ± 0.06	5.61 ± 0.06	6.31 ± 0.07	0.978	1.24 ± 0.13	−4.67 ± 0.99
5.0 ×10^{-5}	2.60 ± 0.06	3.92 ± 0.06	5.50 ± 0.07	6.26 ± 0.06	0.983	1.26 ± 0.12	−4.85 ± 0.88
1.0 ×10^{-4}	2.50 ± 0.04	3.74 ± 0.09	5.37 ± 0.06	6.18 ± 0.07	0.985	1.26 ± 0.11	−5.03 ± 0.83
2.5 ×10^{-4}	2.44 ± 0.03	3.68 ± 0.05	5.30 ± 0.07	6.16 ± 0.06	0.988	1.28 ± 0.10	−5.20 ± 0.76
5.0 ×10^{-4}	2.46 ± 0.03	3.63 ± 0.18	5.31 ± 0.08	6.22 ± 0.03	0.988	1.29 ± 0.10	−5.31 ± 0.75
1.0 ×10^{-3}	2.55 ± 0.02	3.87 ± 0.13	5.54 ± 0.05	6.39 ± 0.08	0.986	1.32 ± 0.11	−5.31 ± 0.83
2.5 ×10^{-3}	2.15 ± 0.40	3.48 ± 0.46	5.18 ± 0.21	6.09 ± 0.09	0.988	1.35 ± 0.11	−5.93 ± 0.80
5.0 ×10^{-3}	2.64 ± 0.11	3.86 ± 0.15	5.44 ± 0.10	6.45 ± 0.08	0.993	1.30 ± 0.07	−5.15 ± 0.57
1.0 ×10^{-2}	2.81 ± 0.03	4.24 ± 0.03	5.48 ± 0.07	6.14 ± 0.08	0.975	1.12 ± 0.13	−3.74 ± 0.96
2.5 ×10^{-2}	2.74 ± 0.06	3.87 ± 0.09	5.18 ± 0.14	6.18 ± 0.04	0.997	1.16 ± 0.04	−4.23 ± 0.32
**Fluorescence ×10^2 (λ_{ex} = 485 nm, λ_{em} = 510 nm)[c]**							
1.0 ×10^{-6}	1.87 ± 0.07	1.94 ± 0.02	2.16 ± 0.03	2.18 ± 0.05	0.910	0.11 ± 0.03	1.18 ± 0.19
1.0 ×10^{-5}	1.90 ± 0.06	2.00 ± 0.03	2.25 ± 0.03	2.26 ± 0.08	0.905	0.13 ± 0.03	1.11 ± 0.23
2.5 ×10^{-5}	1.92 ± 0.02	2.03 ± 0.04	2.28 ± 0.01	2.29 ± 0.04	0.908	0.14 ± 0.03	1.10 ± 0.24
5.0 ×10^{-5}	1.94 ± 0.06	2.05 ± 0.03	2.32 ± 0.04	2.35 ± 0.07	0.922	0.15 ± 0.03	1.03 ± 0.24
1.0 ×10^{-4}	1.94 ± 0.04	2.03 ± 0.03	2.30 ± 0.02	2.39 ± 0.05	0.954	0.16 ± 0.02	0.97 ± 0.19
2.5 ×10^{-4}	1.98 ± 0.03	2.06 ± 0.03	2.42 ± 0.02	2.58 ± 0.02	0.944	0.21 ± 0.04	0.65 ± 0.28
5.0 ×10^{-4}	1.97 ± 0.04	2.03 ± 0.11	2.56 ± 0.08	2.93 ± 0.02	0.929	0.34 ± 0.07	−0.20 ± 0.51
1.0 ×10^{-3}	2.05 ± 0.08	2.26 ± 0.09	3.02 ± 0.04	3.56 ± 0.07	0.962	0.53 ± 0.07	−1.24 ± 0.56
2.5 ×10^{-3}	1.75 ± 0.33	2.17 ± 0.25	3.13 ± 0.16	3.73 ± 0.08	0.980	0.69 ± 0.07	−2.47 ± 0.52
5.0 ×10^{-3}	2.17 ± 0.10	2.48 ± 0.08	3.44 ± 0.13	4.07 ± 0.12	0.969	0.67 ± 0.08	−1.95 ± 0.64
1.0 ×10^{-2}	2.30 ± 0.07	2.66 ± 0.03	3.46 ± 0.04	3.93 ± 0.07	0.981	0.57 ± 0.06	−1.18 ± 0.42
2.5 ×10^{-2}	2.31 ± 0.10	2.53 ± 0.04	3.46 ± 0.11	4.14 ± 0.03	0.953	0.64 ± 0.10	−1.70 ± 0.76
**Normalized Fluorescence ×10^2 (λ_{ex} = 485 nm, λ_{em} = 510 nm, λ_{OD} = 600 nm)[d]**							
1.0 ×10^{-6}	6.66 ± 0.32	4.71 ± 0.12	3.80 ± 0.06	3.36 ± 0.13	0.956	0.92 ± 0.14	1.60 ± 0.07
1.0 ×10^{-5}	6.96 ± 0.25	4.88 ± 0.10	3.98 ± 0.07	3.56 ± 0.09	0.961	1.09 ± 0.16	1.59 ± 0.08
2.5 ×10^{-5}	7.19 ± 0.11	5.00 ± 0.16	4.07 ± 0.03	3.64 ± 0.04	0.965	1.13 ± 0.15	1.60 ± 0.07
5.0 ×10^{-5}	7.45 ± 0.24	5.21 ± 0.12	4.22 ± 0.06	3.75 ± 0.08	0.969	1.22 ± 0.16	1.60 ± 0.07
1.0 ×10^{-4}	7.76 ± 0.08	5.43 ± 0.12	4.28 ± 0.03	3.86 ± 0.04	0.979	1.26 ± 0.13	1.60 ± 0.06
2.5 ×10^{-4}	8.13 ± 0.15	5.60 ± 0.11	4.56 ± 0.03	4.19 ± 0.01	0.959	1.68 ± 0.24	1.52 ± 0.11
5.0 ×10^{-4}	7.99 ± 0.11	5.59 ± 0.06	4.83 ± 0.08	4.72 ± 0.01	0.936	2.64 ± 0.49	1.21 ± 0.23
1.0 ×10^{-3}	8.04 ± 0.29	5.85 ± 0.07	5.46 ± 0.05	5.57 ± 0.08	0.952	3.96 ± 0.63	0.91 ± 0.30
2.5 ×10^{-3}	8.13 ± 0.18	6.26 ± 0.12	6.05 ± 0.08	6.11 ± 0.08	0.983	5.07 ± 0.47	0.55 ± 0.21
5.0 ×10^{-3}	8.22 ± 0.17	6.44 ± 0.06	6.32 ± 0.16	6.32 ± 0.12	0.975	5.12 ± 0.58	0.69 ± 0.28
1.0 ×10^{-2}	8.17 ± 0.14	6.28 ± 0.10	6.31 ± 0.06	6.41 ± 0.10	0.954	4.95 ± 0.77	0.78 ± 0.37
2.5 ×10^{-2}	8.44 ± 0.20	6.55 ± 0.12	6.68 ± 0.03	6.71 ± 0.01	0.956	5.52 ± 0.84	0.63 ± 0.39

[a]data reported as the mean (μ) ± standard deviation (σ) from four (n = 4) measurements at each time-point
[b]linear regression analysis performed for optical density (*y*-axis) as a function of time (*x*-axis) plotted in S9B Fig
[c]linear regression analysis performed for fluorescence (*y*-axis) as a function of time (*x*-axis) plotted in S9C Fig
[d]linear regression analysis performed for fluorescence (*y*-axis) as a function of optical density (*x*-axis)

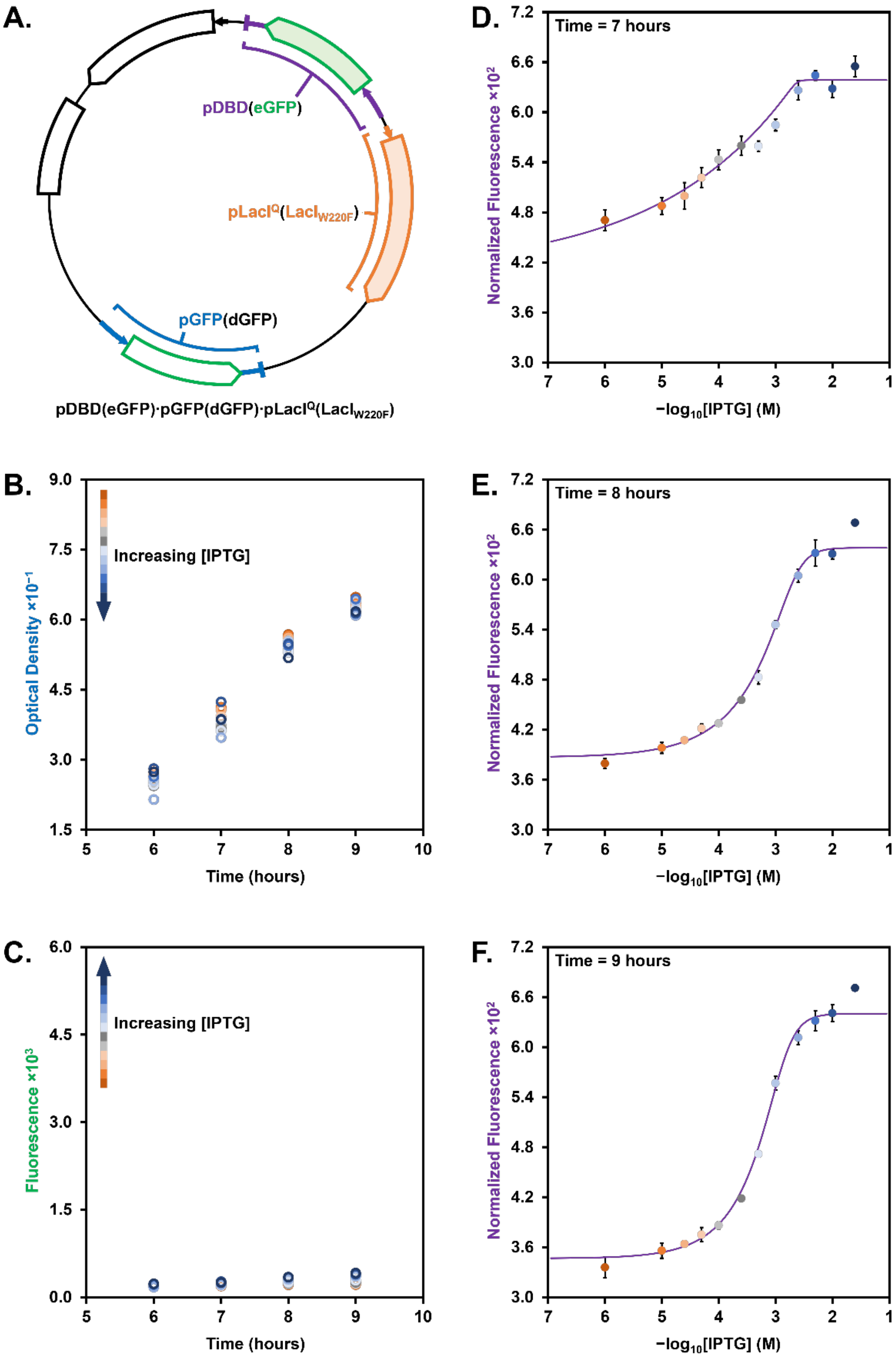

S9 Fig. Inducer series plots for the genetic circuit pDBD(eGFP)·pGFP(dGFP)·pLacI^Q(LacI$_{W220F}$). Data includes the plasmid map for the genetic circuit (A), plot of optical density at 600 nm (B), and culture fluorescence at 510 nm (λ_{ex} = 485 nm) (C), as a function of growth time over the range of IPTG concentrations indicated by the colour scheme, and plots of normalized fluorescence as a function of inducer concentration for 7- (D), 8- (E), and 9- (F) hour time-points.

S8 Table. Inducer series non-linear regression analysis for genetic circuit engineering

Regression Parameters[a]	Time-Course			
	**6 Hours**	**7 Hours**	**8 Hours**	**9 Hours**
**pDBD(eGFP)·pGFP(dGFP)·pLacI(LacI)**				
R^2	0.9995	0.9995	0.9995	0.9994
min[a]	0.0623 ± 0.0020	0.0466 ± 0.0021	0.0400 ± 0.0032	0.0402 ± 0.0038
max[a]	0.3784 ± 0.0035	0.4315 ± 0.0035	0.5276 ± 0.0042	0.5980 ± 0.0051
mid[b]	4.0049 ± 0.0087	4.1493 ± 0.0070	4.2703 ± 0.0065	4.3429 ± 0.0071
slope[c]	2.1331 ± 0.1092	2.2307 ± 0.0848	2.2153 ± 0.0991	2.0164 ± 0.0703
line[c]	0.0483 ± 0.0022	0.0335 ± 0.0024	0.0363 ± 0.0027	0.0263 ± 0.0031
sym[d]	3.7859 ± 0.0285	5.3456 ± 0.0461	4.3470 ± 0.0578	8.0708 ± 0.1666
**pDBD(eGFP)·pGFP(dGFP)·pLacI^Q(LacI)**				
R^2	0.8680	0.9812	0.9921	0.9945
min	0.0536 ± 0.0171	0.0424 ± 0.0011	0.0369 ± 0.0006	0.0328 ± 0.0005
max	0.0754 ± 0.0004	0.0554 ± 0.0002	0.0511 ± 0.0002	0.0495 ± 0.0002
mid	6.0704 ± 2.3673	4.4463 ± 0.1354	4.1109 ± 0.0584	4.1024 ± 0.0408
slope	11.7003 ± 14.9288	2.5509 ± 0.5360	2.0017 ± 0.2173	1.6097 ± 0.1341
sym	0.0260 ± 0.0001	0.1447 ± 0.0008	0.2611 ± 0.0020	0.4322 ± 0.0076
**pDBD(eGFP)·pGFP(dGFP)·pLacI(LacI$_{W220F}$)**				
R^2	0.9989	0.9998	0.9997	0.9995
min	0.0688 ± 0.0026	0.0481 ± 0.0014	0.0415 ± 0.0023	0.0406 ± 0.0037
max	0.4677 ± 0.0108	0.6019 ± 0.0034	0.7254 ± 0.0056	0.8402 ± 0.0087
mid	3.2564 ± 0.0171	3.4223 ± 0.0046	3.5802 ± 0.0056	3.6899 ± 0.0077
slope	1.8769 ± 0.1326	2.2466 ± 0.0527	2.2234 ± 0.1049	2.1441 ± 0.0886
line	0.0872 ± 0.0091	0.0911 ± 0.0041	0.0598 ± 0.0051	0.0335 ± 0.0072
sym	5.9632 ± 0.0373	3.3020 ± 0.0131	3.7844 ± 0.0446	6.9882 ± 0.2435
**pDBD(eGFP)·pGFP(dGFP)·pLacI^Q(LacI$_{W220F}$)**				
R^2	0.9581	0.9764	0.9891	0.9917
min	0.0634 ± 0.0046	0.0409 ± 0.0051	0.0387 ± 0.0009	0.0347 ± 0.0007
max	0.0816 ± 0.0004	0.0639 ± 0.0006	0.0638 ± 0.0007	0.0640 ± 0.0007
mid	4.5725 ± 0.4716	4.2508 ± 0.6743	3.1634 ± 0.0600	3.2411 ± 0.0435
slope	16.7257 ± 6.9184	9.5186 ± 2.9403	2.5101 ± 0.3626	2.4213 ± 0.3050
sym	0.0256 ± 0.0001	0.0195 ± 0.0001	0.2316 ± 0.0038	0.3090 ± 0.0056

[a]regression parameters for minimum expression (*min*) and maximum expression (*max*) asymptotes, sigmoid curve midpoint (*mid*), growth of the sigmoid curve at inflection (*slope*), linear scaling of the maximum asymptote (*line*), and symmetry factor altering the shape of the sigmoid curve (*sym*), reported for plots of Fluorescence ÷ Optical Density × 10^4 versus $-\log_{10}$[IPTG] (M)

Section 4. Expression Controls for the three-Component Genetic Circuit

Overview of Experiments to Define Genetic Circuit Expression Controls. To demarcate the minimum and maximum output signals produced by the genetic circuit in both the absence and presence of inducer (10 mM IPTG), density-normalized culture fluorescence was monitored for a 6 to 9-hour time-course for genetic circuits outfitted with combinations of the reporter protein (eGFP) and its decoy counterpart (dGFP) inserted into Cloning Sites I and II under the control of promoters pDBD and pGFP, respectively. The identity and purpose of these control plasmids include:

S10 Fig: the genetic circuit pDBD(dGFP)·pGFP(dGFP)·pLacI(LacI$_{W220F}$) establishing output of basal culture fluorescence originating from non-reporter sources,

S11 Fig: the genetic circuit pDBD(eGFP)·pGFP(dGFP)·pLacI(LacI$_{W220F}$) establishing expression output from the pDBD promoter of Cloning Site I responsible for expressing the experimental single-chain tandem repeat repressor,

S12 Fig: the genetic circuit pDBD(dGFP)·pGFP(eGFP)·pLacI(LacI$_{W220F}$) establishing expression output from the pGFP promoter of Cloning Site II responsible for expressing the reporting protein for the genetic circuit, and

S13 Fig: the genetic circuit pDBD(eGFP)·pGFP(eGFP)·pLacI(LacI$_{W220F}$) establishing expression output from both the pDBD and pGFP promoters of Cloning Sites I and II thereby establishing output signal fidelity and stability of the genetic circuit.

Establishing Baseline Fluorescence for the Genetic Circuit. To define the baseline fluorescence output that occurs in the absence of eGFP expression, the dGFP gene was inserted into both Cloning Sites I and II producing the pDBD(dGFP)·pGFP(dGFP)pLacI(LacI$_{W220F}$) genetic circuit (S10A Fig). Density-normalized culture fluorescence (F) was measured as a function of time in the absence (F_{-IPTG}) and presence (F_{+IPTG}) of 10 mM IPTG inducer (S10 Fig, panels C and D). For all time-course measurements, no significant difference in fluorescence between induced and repressed growth conditions was observed, as expected. For example, density-normalized culture fluorescence recorded at the 8-hour time-point collected in the absence of IPTG (F_{-IPTG} = 360 ± 10) was similar (p-value = 0.005, two-tailed homoscedastic t-test) to the output signal obtained for cultures grown in the presence of 10 mM IPTG (F_{+IPTG} = 390 ± 10).

> **Baseline Fluorescence Conclusion.** These measurements establish the range in baseline output signal for background fluorescence that defines the minimum values that can be obtained using this genetic circuit setup.

Characterization of Expression Output for the pDBD Promoter. To evaluate pDBD promoter activity, the dGFP gene at Cloning Site I was replaced with a fluorescently active copy of the eGFP to create the pDBD(eGFP)·pGFP(dGFP)·pLacI(LacI$_{W220F}$) genetic circuit (S11 Fig). Density-normalized culture fluorescence measured at the 8-hour time-point demonstrated a significant (p-value < 0.001, two-tailed homoscedastic t-test) 19.7 ± 0.8 fold increase in output signal upon induction with 10 mM IPTG (F_{+IPTG} = 7,300 ± 200 and F_{-IPTG} = 370 ± 10). Importantly, the low F_{-IPTG} value was indistinguishable from baseline values (p-value = 0.207, two-tailed homoscedastic t-test) confirming the absence of leaky-expression originating from the pDBD promoter as also demonstrated in the previous section (Tuning Repressor Saturation of pDBD).

> **Cloning Site I Promoter Output Conclusion.** These observations establish the utility of pDBD, having negligible leaky-expression and significant dynamic range for the expression of the gene that is placed under the control of this promoter, validating its utility for the evaluation of experimental single-chain tandem repeat repressors created in this study.

Characterization of Expression Output for the pGFP Promoter. The genetic circuit was designed to function through the concerted activity of Cloning Sites I and II to control pDBD-driven expression of an experimental repressor (scDBD in this study) and report on its ability to repress expression of the fluorescent reporter from pGFP. Because the pDBD-pGFP promoter pair functions interdependently there is the potential that induction of expression from pDBD may impose a metabolic burden that would reduce expression from pGFP by competing for limited endogenous transcription and translation factor resources which would complicate interpretation of the genetic circuit output data [6]. To investigate this possibility, pGFP-driven expression activity was evaluated in the presence of constitutive pDBD expression. This involved a control genetic circuit constructed with copies of dGFP and eGFP genes inserted into cloning Sites I and II, respectively, to produce pDBD(dGFP)·pGFP(eGFP)·pLacI(LacI$_{W220F}$) (S12 Fig). Between 6

and 9-hour time-points, similar linear relationships were observed for optical denisty (S12 Fig, panels C and D) and fluorescence (S12 Fig, panels E and F) both in the presence and absence of 10 mM IPTG. No significant difference (p-value = 0.011, two-tailed homoscedastic t-test) in density-normalized culture fluorescence was observed in the presence and absence of IPTG (F_{-IPTG} = 19,900 ± 400 and F_{+IPTG} = 18,700 ± 600) at the 8-hour time-point (S12B Fig) while measurement for the 9-hour time-point indicates IPTG-dependent attenuation (p-value < 0.001, two-tailed homoscedastic t-test) of pGFP activity (F_{-IPTG} = 26,800 ± 300 and F_{+IPTG} = 24,500 ± 400). This IPTG-dependent reduction in pGFP activity recorded at the 9-hour time-point decreased expression output by approximately 8.6 ± 1.9 % despite the fact that the pGFP promoter is approximately 2.85 ± 0.06 fold stronger than pDBD (calculated from F_{+IPTG} for pDBD(dGFP)·pGFP(eGFP)·pLacI($LacI_{W220F}$) divided by pDBD(eGFP)·pGFP(dGFP)·pLacI($LacI_{W220F}$) at the 8-hour time-point).

Cloning Site II Promoter Output Conclusion. These results demonstrate a mild degree of IPTG-imposed metabolic burden on pGFP for time-course data exceeding the 8-hour time-point and establish the range in maximum output values that can be obtained using this genetic circuit setup. Importantly, all measurements taken within the 8-hour time-point demonstrate that induction of Site I expression does not significantly impact expression levels from Site II, indicating that sufficient metabolic resources exist for the expression of targets from both promoters simultaneously.

Evaluation of Expression from both Cloning Sites I and II. To examine the effect of expression from both Cloning Sites I and II simultaneously, a copy of the eGFP gene was inserted into both Cloning Sites I and II, producing the pDBD(eGFP)·pGFP(eGFP)·pLacI($LacI_{W220F}$) genetic circuit (S13 Fig). Density-normalized culture fluorescence showed no IPTG-dependent change in output signal at the 6-, 7- and 8-hour time-points (*e.g.,* 8-hour time-point comparison between F_{-IPTG} = 22,400 ± 700 and F_{+IPTG} = 21,700 ± 100, p-value = 0.100, two-tailed homoscedastic t-test). However, a quantifiable difference (p-value = 0.004, two-tailed homoscedastic t-test) for the 9-hour time-point (F_{-IPTG} = 27,600 ± 500 and F_{+IPTG} = 26,200 ± 400) suggests that induction with 10 mM might introduce a mild metabolic burden on the host.

Genetic Circuit Output Stability Conclusion. These results demonstrate that the genetic circuit operates to produce a quantifiably stable level of output expression that is not significantly affected by induction of expression from pDBD.

Demonstrating Steady-state Behaviour for Genetic Circuit Outputs. Because genetic circuit output is measured as a function of the density-normalized culture fluorescence, it is important that the linearity of control genetic circuits is evaluated across a time-course to establish a regiment of steady-state behaviour. Linear regression analysis was performed for the set of optical density and fluorescence outputs from each of the four control genetic circuits, demonstrating linear (*i.e.,* steady-state) correlations as a function of time (S9 Table). Specifically, linear regression reveals strong time-dependent correlations for both optical density (R^2 = 0.983 − 0.995) and fluorescence (R^2 = 0.845 − 0.996), irrespective of the presence of inducer. These results confirm that results collected between the 6- to 9-hour time-point should be qualitatively comparable and quantitatively consistent.

Summary of Genetic Circuit Control Results. Four genetic circuit controls incorporating combinations of eGFP and dGFP genes into Cloning Sites I and II were evaluated at the 8-hour time-point (S14 Fig). Measurements of the pDBD(dGFP)·pGFP(dGFP)·pLacI($LacI_{W220F}$) control establish a baseline for minimum output fluorescence between 360 ± 10 and 390 ± 10 (F_{-IPTG} and F_{+IPTG}, respectively). Induction of the pDBD promoter was shown to minimally interfere with expression activity of pGFP as evaluated with the pDBD(dGFP)·pGFP(eGFP)·pLacI($LacI_{W220F}$) control in the absence (F_{-IPTG} = 19,900 ± 400) and presence (F_{+IPTG} = 18,700 ± 600) of inducer having no significant difference (p-value = 0.011). Furthermore, no leaky expression from pDBD was observed when comparing the pDBD(eGFP)·pGFP(dGFP)·pLacI($LacI_{W220F}$) control (F_{-IPTG} = 370 ± 10) against the established baseline (F_{-IPTG} = 360 ± 10). These experiments demonstrate that the single-plasmid three-component genetic circuit, whose annotated sequence is reported in S5 Fig, will enable the quantitative and reproducible evaluation of functions for designed scDBD repressor constructs. Moreover, this single-plasmid three-component genetic circuit has the potential to be adapted for the evaluation of genetic circuits beyond the experimental repressors that were evaluated in this work, including: the constitutive co-expression of a fluorescent protein control for high-throughput protein engineering using fluorescence activated cell sorting [9], the evaluation of alternate genetic regulatory constructs [10], the design of multicomponent genetic logic gates [11], and the construction of genetic regulatory networks involving a series of gene expression events [12].

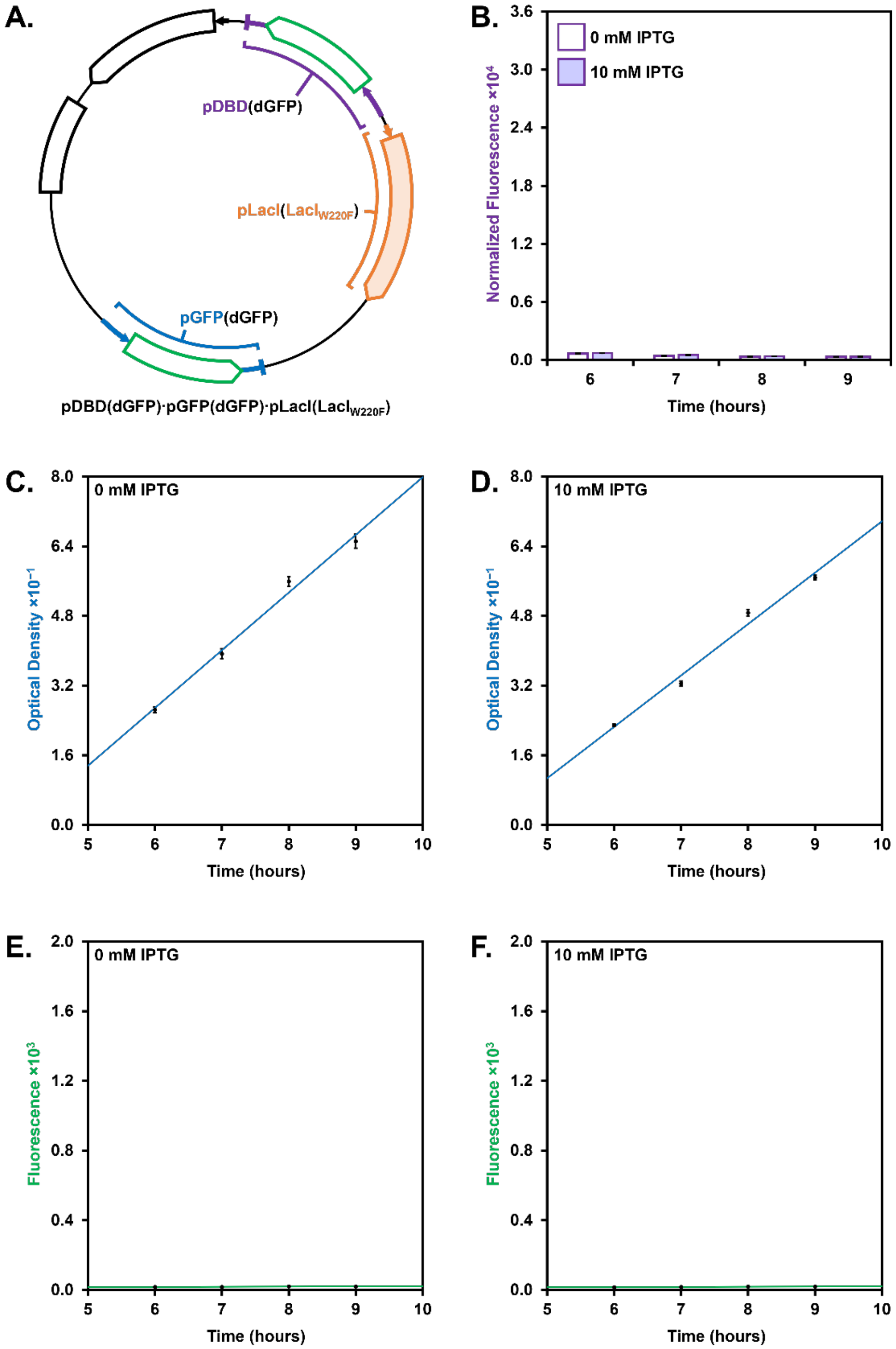

S10 Fig. Expression controls for the pDBD(dGFP)·pGFP(dGFP)·pLacI(LacI$_{W220F}$) genetic circuit. Data includes the plasmid map for the genetic circuit (A) and time-course summary for the normalized fluorescence from the genetic circuit (B) with *p*-values indicated for statistically significant (p-value < 0.001) changes in output signal. Plots include culture density (*y*-axis) as a function of growth time (*x*-axis) in the absence (C) and presence (D) of 10 mM IPTG, and culture fluorescence (*y*-axis) as a function of growth time (*x*-axis) in the absence (E) and presence (F) of 10 mM IPTG.

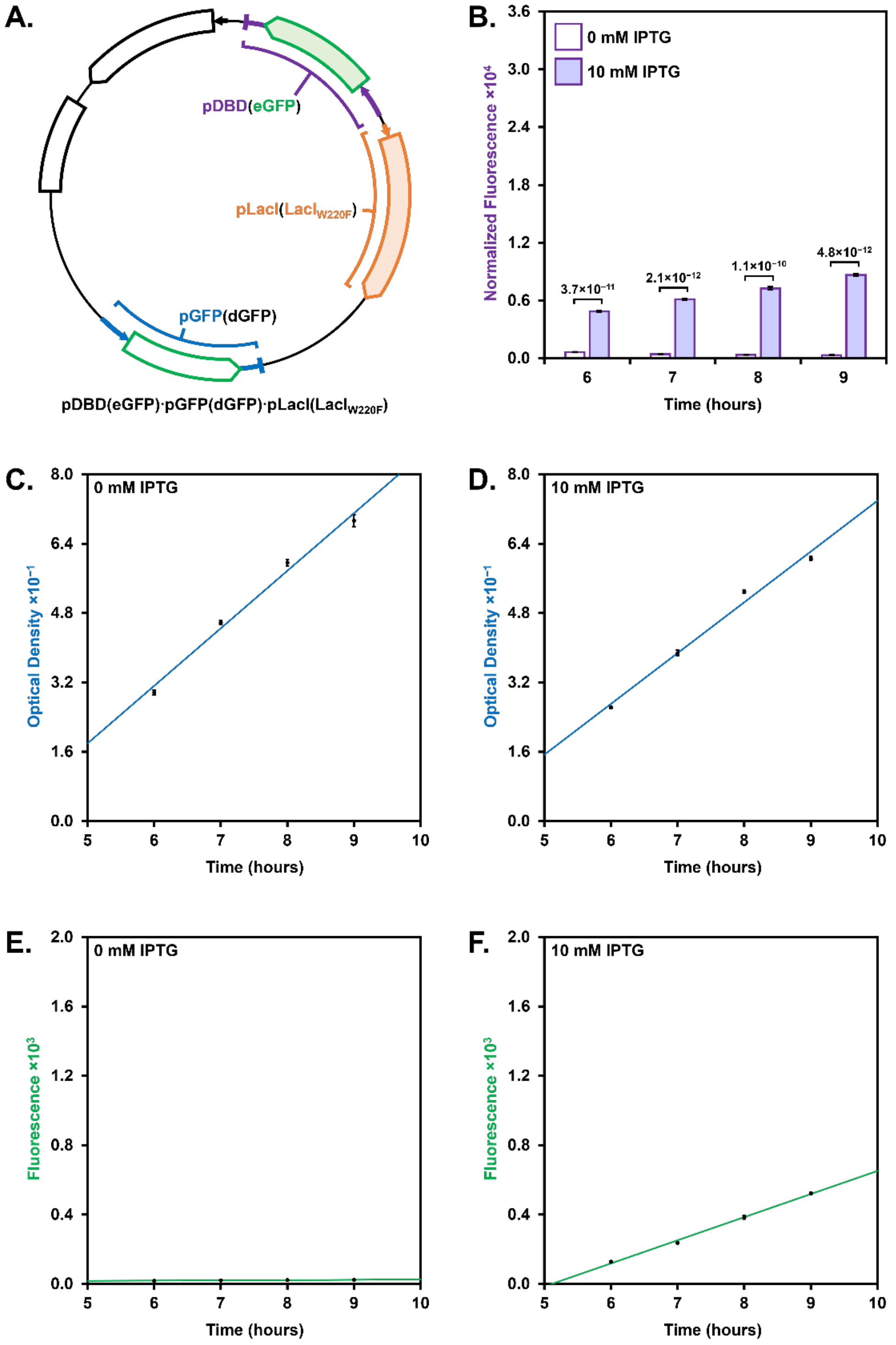

S11 Fig. Expression controls for the pDBD(eGFP)·pGFP(dGFP)·pLacI(LacI$_{W220F}$) genetic circuit. Data includes the plasmid map for the genetic circuit (A) and time-course summary for the normalized fluorescence from the genetic circuit (B) with *p*-values indicated for statistically significant (p-value < 0.001) changes in output signal. Plots include culture density (*y*-axis) as a function of growth time (*x*-axis) in the absence (C) and presence (D) of 10 mM IPTG, and culture fluorescence (*y*-axis) as a function of growth time (*x*-axis) in the absence (E) and presence (F) of 10 mM IPTG.

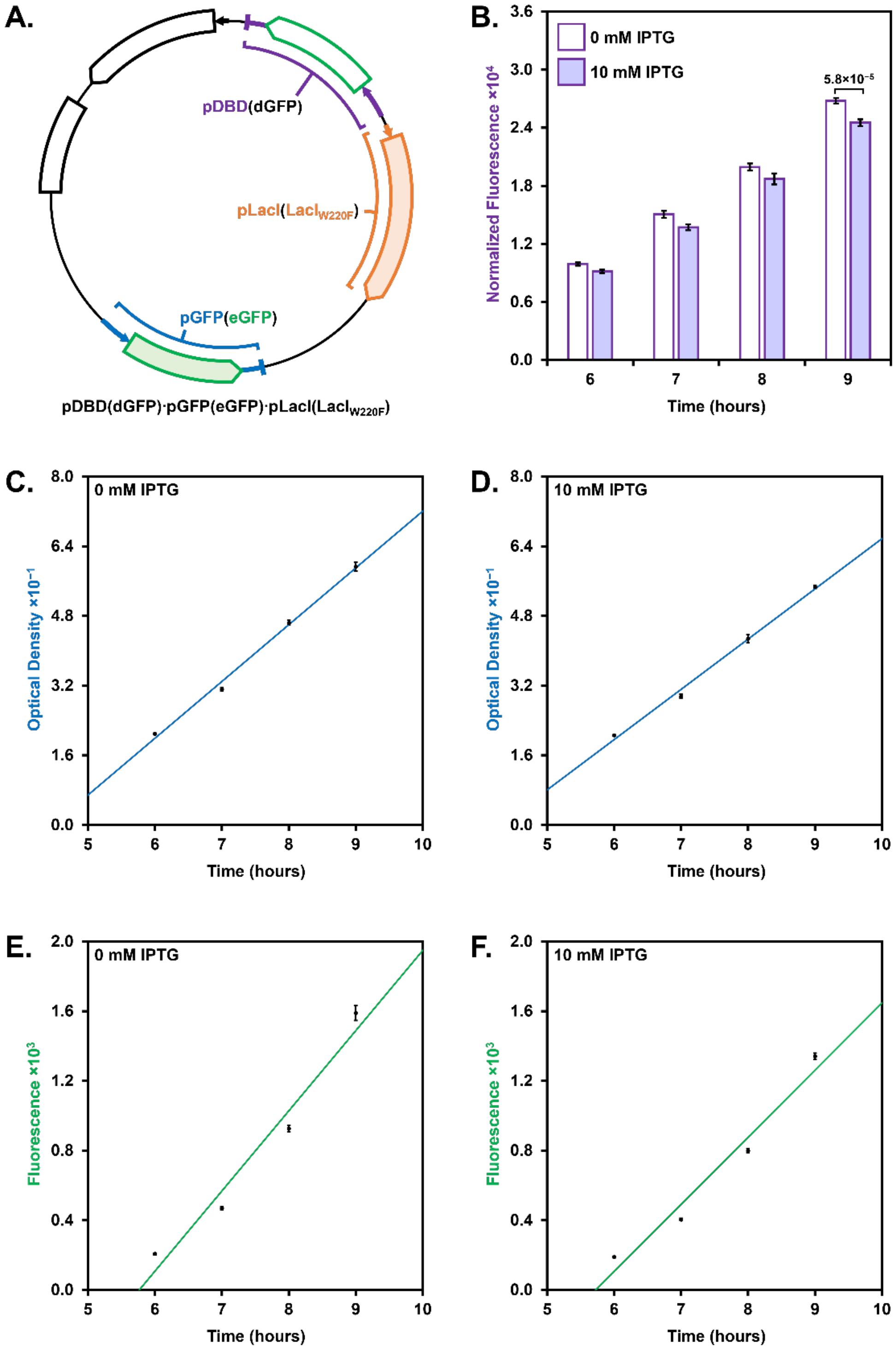

S12 Fig. Expression controls for the pDBD(dGFP)·pGFP(eGFP)·pLacI(LacI$_{W220F}$) genetic circuit. Data includes the plasmid map for the genetic circuit (A) and time-course summary for the normalized fluorescence from the genetic circuit (B) with *p*-values indicated for statistically significant (p-value < 0.001) changes in output signal. Plots include culture density (*y*-axis) as a function of growth time (*x*-axis) in the absence (C) and presence (D) of 10 mM IPTG, and culture fluorescence (*y*-axis) as a function of growth time (*x*-axis) in the absence (E) and presence (F) of 10 mM IPTG.

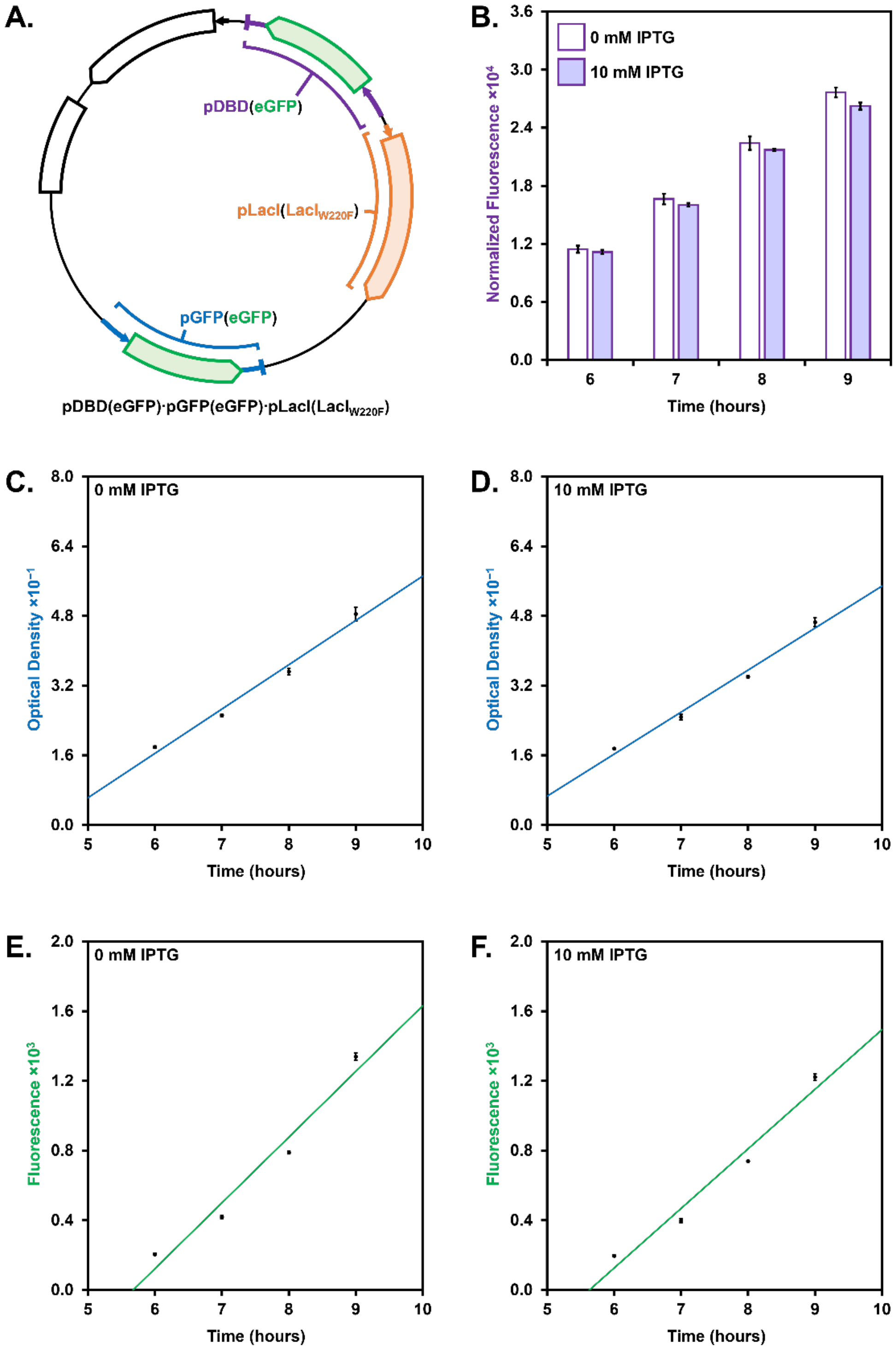

S13 Fig. Expression controls for the pDBD(eGFP)·pGFP(eGFP)·pLacI(LacI$_{W220F}$) genetic circuit. Data includes the plasmid map for the genetic circuit (A) and time-course summary for the normalized fluorescence from the genetic circuit (B) with *p*-values indicated for statistically significant (*p*-value < 0.001) changes in output signal. Plots include culture density (*y*-axis) as a function of growth time (*x*-axis) in the absence (C) and presence (D) of 10 mM IPTG, and culture fluorescence (*y*-axis) as a function of growth time (*x*-axis) in the absence (E) and presence (F) of 10 mM IPTG.

S9 Table. Regression analysis of expression controls for the pDBD·pGFP·pLacI($LacI_{W220F}$) genetic circuit

Cloning Site		0 mM IPTG Culture			10 mM IPTG Culture		
I[a]	II[b]	R^2	slope	intercept	R^2	slope	intercept
**Optical Density vs Time[c,d] (hours)**							
eGFP	eGFP	0.983	1.02 ± 0.09 ×10^{-1}	−4.47 ± 0.71 ×10^{-1}	0.985	0.96 ± 0.08 ×10^{-1}	−4.16 ± 0.64 ×10^{-1}
eGFP	dGFP	0.988	1.33 ± 0.10 ×10^{-1}	−4.85 ± 0.78 ×10^{-1}	0.987	1.17 ± 0.10 ×10^{-1}	−4.33 ± 0.73 ×10^{-1}
dGFP	eGFP	0.995	1.30 ± 0.07 ×10^{-1}	−5.83 ± 0.51 ×10^{-1}	0.994	1.15 ± 0.06 ×10^{-1}	−4.97 ± 0.47 ×10^{-1}
dGFP	dGFP	0.989	1.33 ± 0.10 ×10^{-1}	−5.27 ± 0.74 ×10^{-1}	0.984	1.18 ± 0.11 ×10^{-1}	−4.83 ± 0.81 ×10^{-1}
**Fluorescence vs Time[e] (hours)**							
eGFP	eGFP	0.962	0.38 ± 0.05 ×10^{4}	−2.14 ± 0.40 ×10^{4}	0.967	0.34 ± 0.05 ×10^{4}	−1.93 ± 0.34 ×10^{4}
eGFP	dGFP	0.991	0.14 ± 0.01 ×10^{2}	1.03 ± 0.07 ×10^{2}	0.996	1.34 ± 0.06 ×10^{3}	−6.83 ± 0.43 ×10^{3}
dGFP	eGFP	0.963	4.60 ± 0.64 ×10^{3}	−2.65 ± 0.48 ×10^{4}	0.965	3.85 ± 0.52 ×10^{3}	−2.21 ± 0.39 ×10^{4}
dGFP	dGFP	0.845	0.11 ± 0.03 ×10^{0}	1.02 ± 0.26 ×10^{2}	0.961	0.12 ± 0.02 ×10^{2}	0.93 ± 0.13 ×10^{2}
**Fluorescence vs Optical Density**							
eGFP	eGFP	0.996	3.74 ± 0.18 ×10^{4}	−0.50 ± 0.06 ×10^{4}	0.996	3.57 ± 0.15 ×10^{4}	−0.46 ± 0.05 ×10^{4}
eGFP	dGFP	0.995	1.08 ± 0.05 ×10^{2}	1.55 ± 0.03 ×10^{2}	0.979	1.12 ± 0.12 ×10^{4}	−0.18 ± 0.05 ×10^{4}
dGFP	eGFP	0.975	3.54 ± 0.40 ×10^{4}	−0.60 ± 0.17 ×10^{4}	0.982	3.36 ± 0.32 ×10^{4}	−0.55 ± 0.13 ×10^{4}
dGFP	dGFP	0.902	0.89 ± 0.21 ×10^{2}	1.46 ± 0.10 ×10^{2}	0.994	1.00 ± 0.05 ×10^{2}	1.41 ± 0.02 ×10^{2}

[a]inducible expression from cloning Site I under the control of the pDBD promoter
[b]constitutive expression from cloning Site II under the control of the pGFP promoter
[c]data collected at 6-, 7-, 8-, and 9-hour time-points in quadruplicate (n = 4)
[d]plots shown in panels C and D of S10–S13 Figs
[e]plots shown in panels E and F of S10–S13 Figs

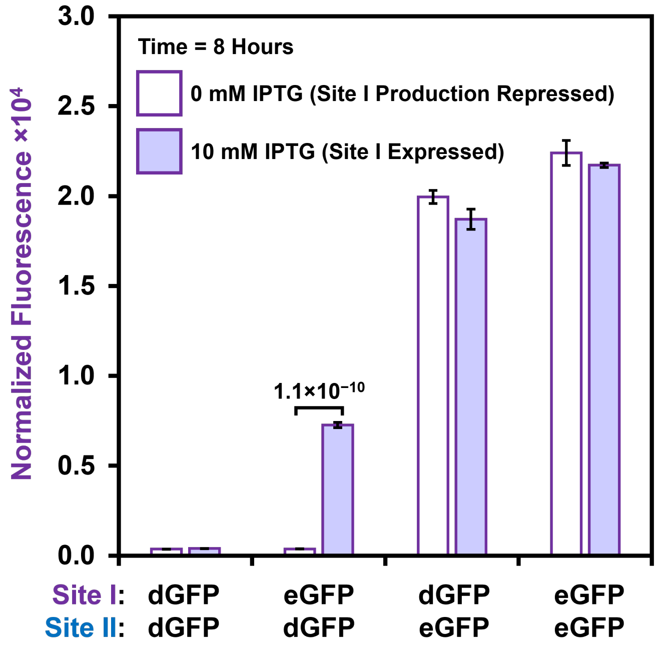

S14 Fig. Summary of expression controls for the three-component genetic circuit. A comparison of expression output from Cloning Sites I and II of the genetic circuit was compiled by monitoring normalized output fluorescence for arrayed combinations of eGFP and dGFP. A two-tailed homoscedastic *t*-test was performed comparing genetic circuit output in the absence and presence of inducer (10 mM IPTG) with *p*-values reported for populations exhibiting statistically significant difference (*p*-value < 0.001, two-tailed homoscedastic *t*-test, replicates = 4).

Section 5. Characterization of scDBD Repressor Function

The evaluation of scDBD repressor function as described in the Results section is supported with the supplementary material provided here. The pDBD(scDBD)·pGFP(eGFP)·pLacI($LacI_{W220F}$) genetic circuit was employed to characterize the function of scDBD repressor candidates by inserting a copy of a scDBD-encoding gene into Cloning Site I and assaying culture expression over a time-course, collecting culture density and fluorescence readings at 6-, 7-, 8-, and 9-hour time-points. Repressor variants were designed joining pairs of functional (IAN: **I**17/**A**18/**N**22) and non-function (DFT: **D**17/**F**18/**T**22) N- and C-terminal DBDs (DBD.N and DBD.C, respectively). In total, the activities of eight scDBD repressor constructs were evaluated, including the four full-length TR constructs:

S15 Fig: $scDBD_{IAN/IAN}$,
S16 Fig: $scDBD_{DFT/IAN}$,
S17 Fig: $scDBD_{IAN/DFT}$, and
S18 Fig: $scDBD_{DFT/DFT}$.

Repressor variants also include the four C-terminal truncated (deletion of the 29-last residues) variants:

S19 Fig: $scDBD_{IAN/IAN/\Delta CT}$,
S20 Fig: $scDBD_{DFT/IAN/\Delta CT}$,
S21 Fig: $scDBD_{IAN/DFT/\Delta CT}$, and
S22 Fig: $scDBD_{DFT/DFT/\Delta CT}$.

Regression analysis of the expression time-courses (S10 Table) demonstrates that repressor activities are all measured at steady-state. Examination of the data indicates that the assembly of functional scDBD repressors requires incorporation of two functional DBDs (IAN).

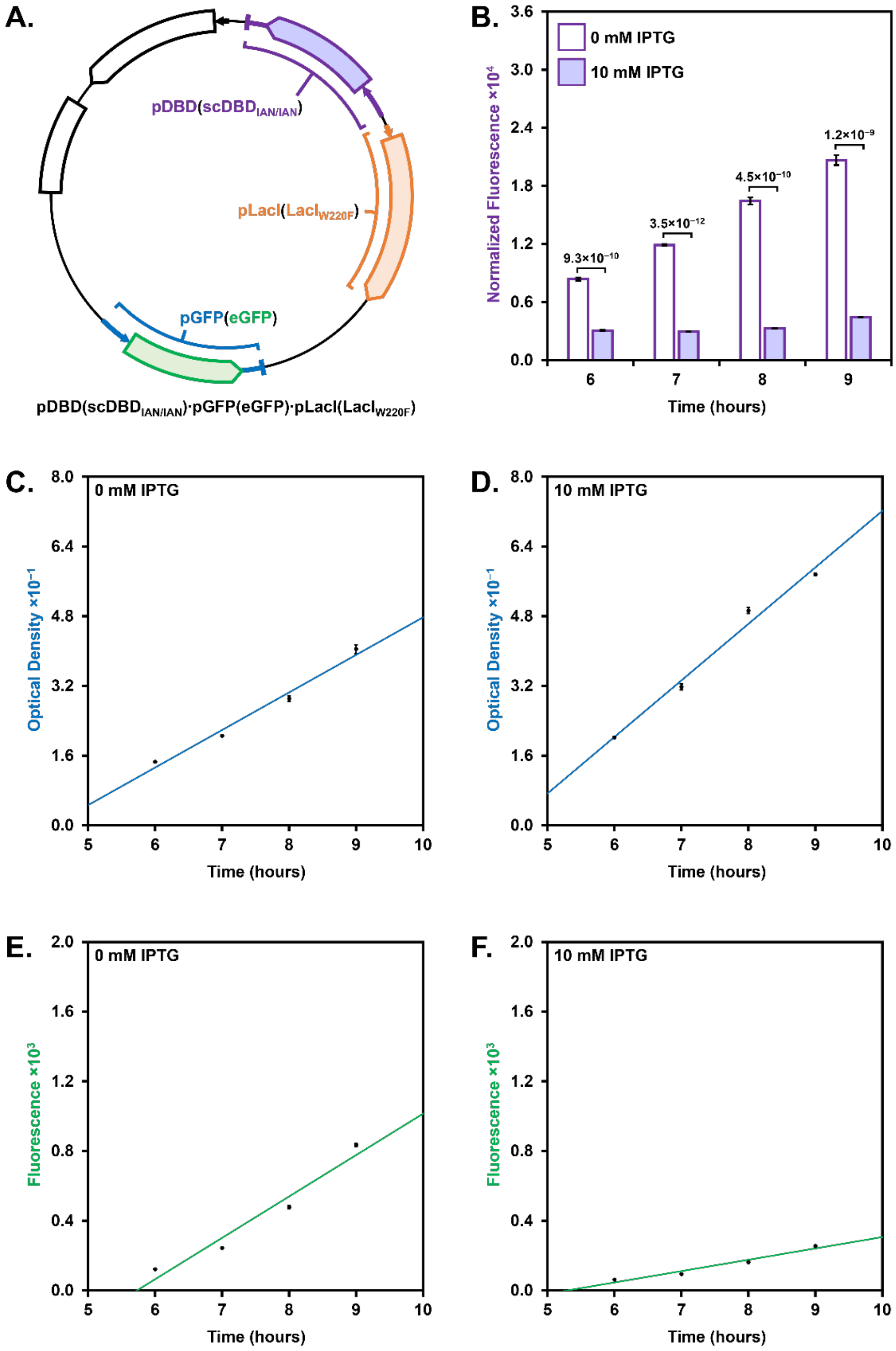

S15 Fig. Expression plots for the pDBD($scDBD_{IAN/IAN}$)·pGFP(eGFP)·pLacI($LacI_{W220F}$) genetic circuit. Data includes the plasmid map for the genetic circuit (A) and time-course summary for the normalized fluorescence from the genetic circuit (B) with *p*-values indicated for statistically significant (*p*-value < 0.001, two-tailed homoscedastic *t*-test, replicates = 4) changes in output signal. Plots include culture density (*y*-axis) as a function of growth time (*x*-axis) in the absence (C) and presence (D) of 10 mM IPTG, and culture fluorescence (*y*-axis) as a function of growth time (*x*-axis) in the absence (E) and presence (F) of 10 mM IPTG.

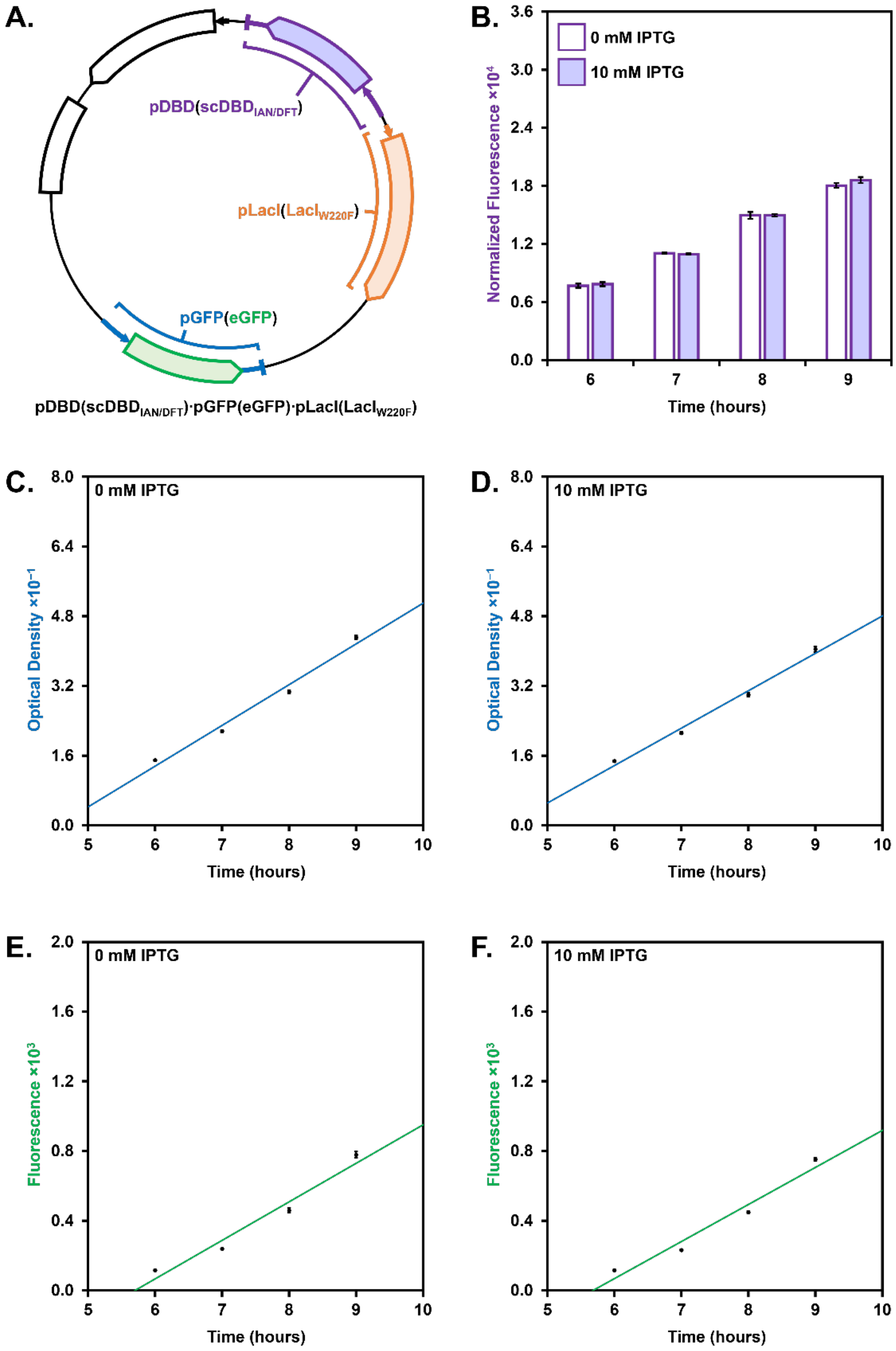

S16 Fig. Expression plots for the pDBD(scDBD$_{IAN/DFT}$)·pGFP(eGFP)·pLacI(LacI$_{W220F}$) genetic circuit. Data includes the plasmid map for the genetic circuit (A) and time-course summary for the normalized fluorescence from the genetic circuit (B) with *p*-values indicated for statistically significant (*p*-value < 0.001, two-tailed homoscedastic *t*-test, replicates = 4) changes in output signal. Plots include culture density (*y*-axis) as a function of growth time (*x*-axis) in the absence (C) and presence (D) of 10 mM IPTG, and culture fluorescence (*y*-axis) as a function of growth time (*x*-axis) in the absence (E) and presence (F) of 10 mM IPTG.

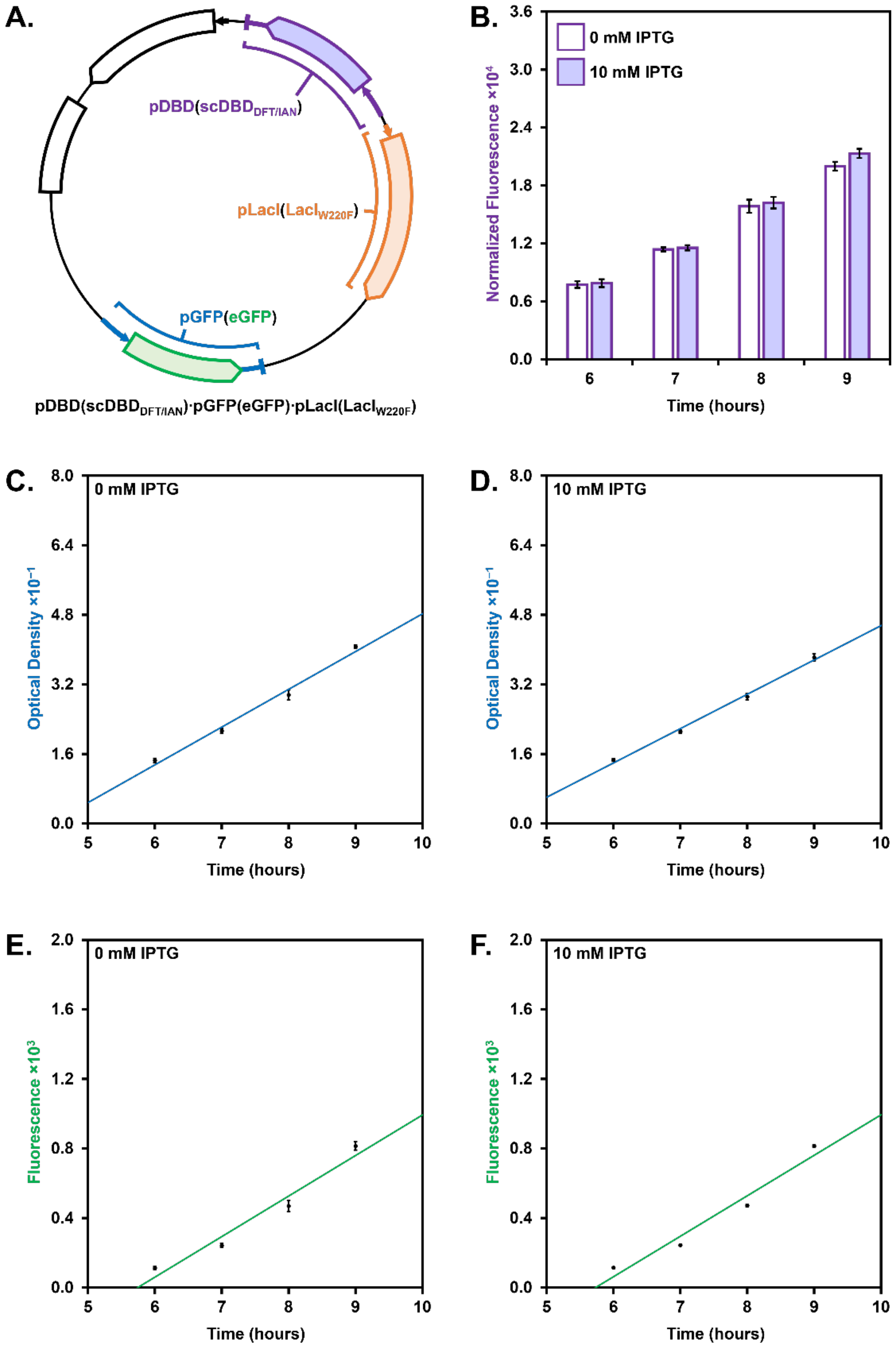

S17 Fig. Expression plots for the pDBD(scDBD$_{DFT/IAN}$)·pGFP(eGFP)·pLacI(LacI$_{W220F}$) genetic circuit. Data includes the plasmid map for the genetic circuit (A) and time-course summary for the normalized fluorescence from the genetic circuit (B) with *p*-values indicated for statistically significant (p-value < 0.001, two-tailed homoscedastic *t*-test, replicates = 4) changes in output signal. Plots include culture density (*y*-axis) as a function of growth time (*x*-axis) in the absence (C) and presence (D) of 10 mM IPTG, and culture fluorescence (*y*-axis) as a function of growth time (*x*-axis) in the absence (E) and presence (F) of 10 mM IPTG.

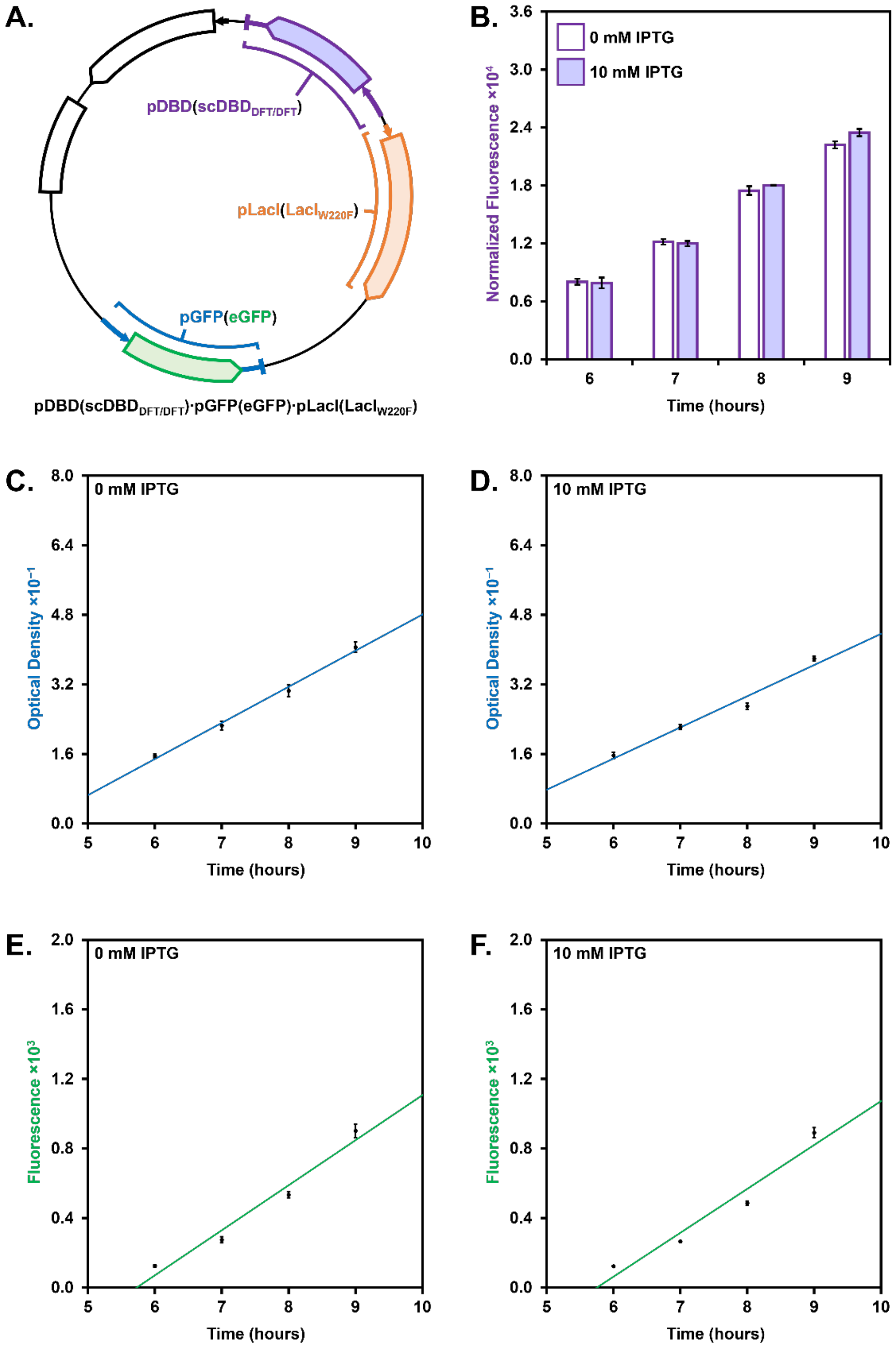

S18 Fig. Expression plots for the pDBD(scDBD$_{DFT/DFT}$)·pGFP(eGFP)·pLacI(LacI$_{W220F}$) genetic circuit. Data includes the plasmid map for the genetic circuit (A) and time-course summary for the normalized fluorescence from the genetic circuit (B) with *p*-values indicated for statistically significant (*p*-value < 0.001, two-tailed homoscedastic *t*-test, replicates = 4) changes in output signal. Plots include culture density (*y*-axis) as a function of growth time (*x*-axis) in the absence (C) and presence (D) of 10 mM IPTG, and culture fluorescence (*y*-axis) as a function of growth time (*x*-axis) in the absence (E) and presence (F) of 10 mM IPTG.

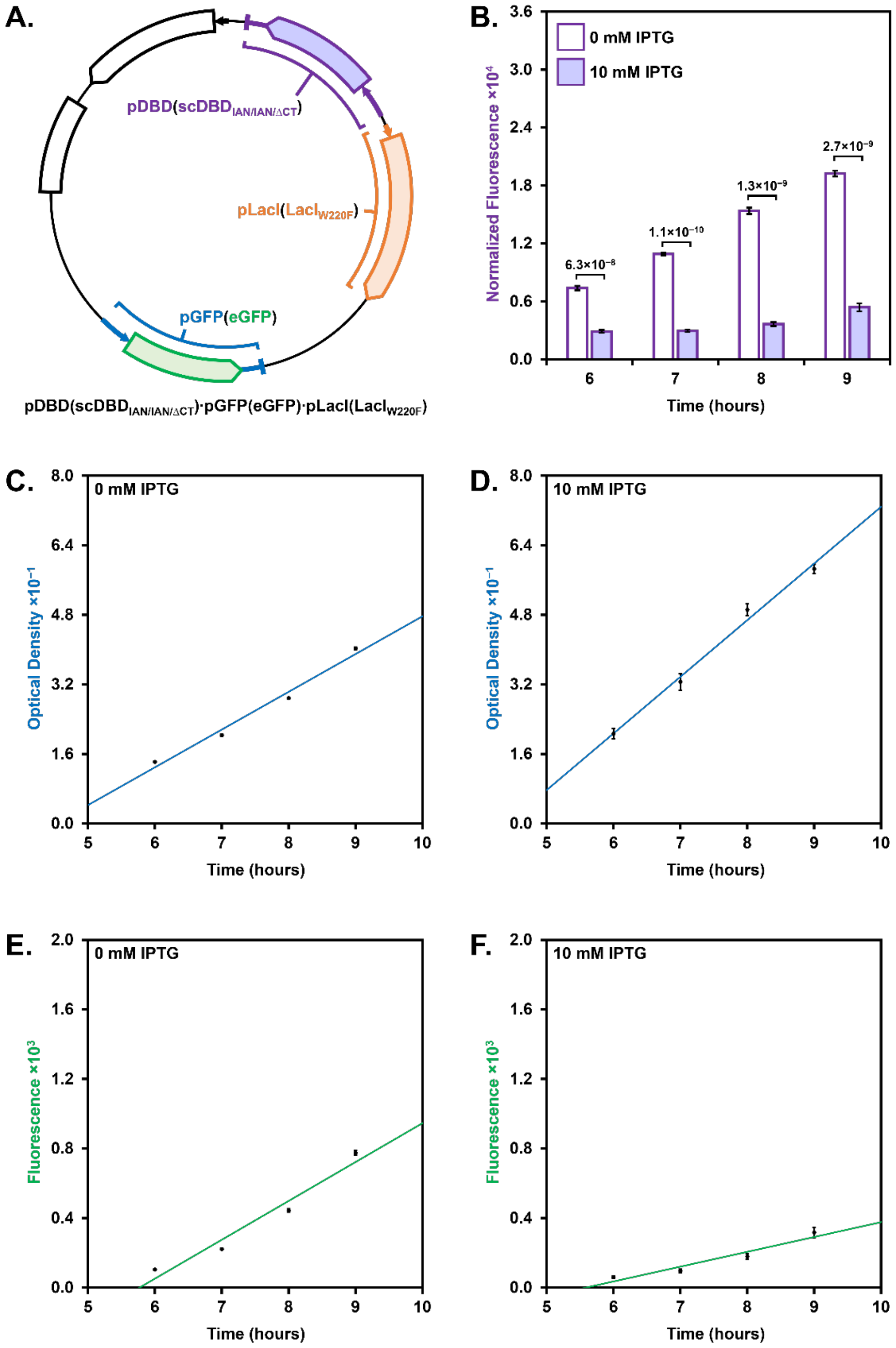

S19 Fig. Expression plots for the pDBD($scDBD_{IAN/IAN/\Delta CT}$)·pGFP(eGFP)·pLacI($LacI_{W220F}$) genetic circuit. Data includes the plasmid map for the genetic circuit (A) and time-course summary for the normalized fluorescence from the genetic circuit (B) with *p*-values indicated for statistically significant (*p*-value < 0.001, two-tailed homoscedastic *t*-test, replicates = 4) changes in output signal. Plots include culture density (*y*-axis) as a function of growth time (*x*-axis) in the absence (C) and presence (D) of 10 mM IPTG, and culture fluorescence (*y*-axis) as a function of growth time (*x*-axis) in the absence (E) and presence (F) of 10 mM IPTG.

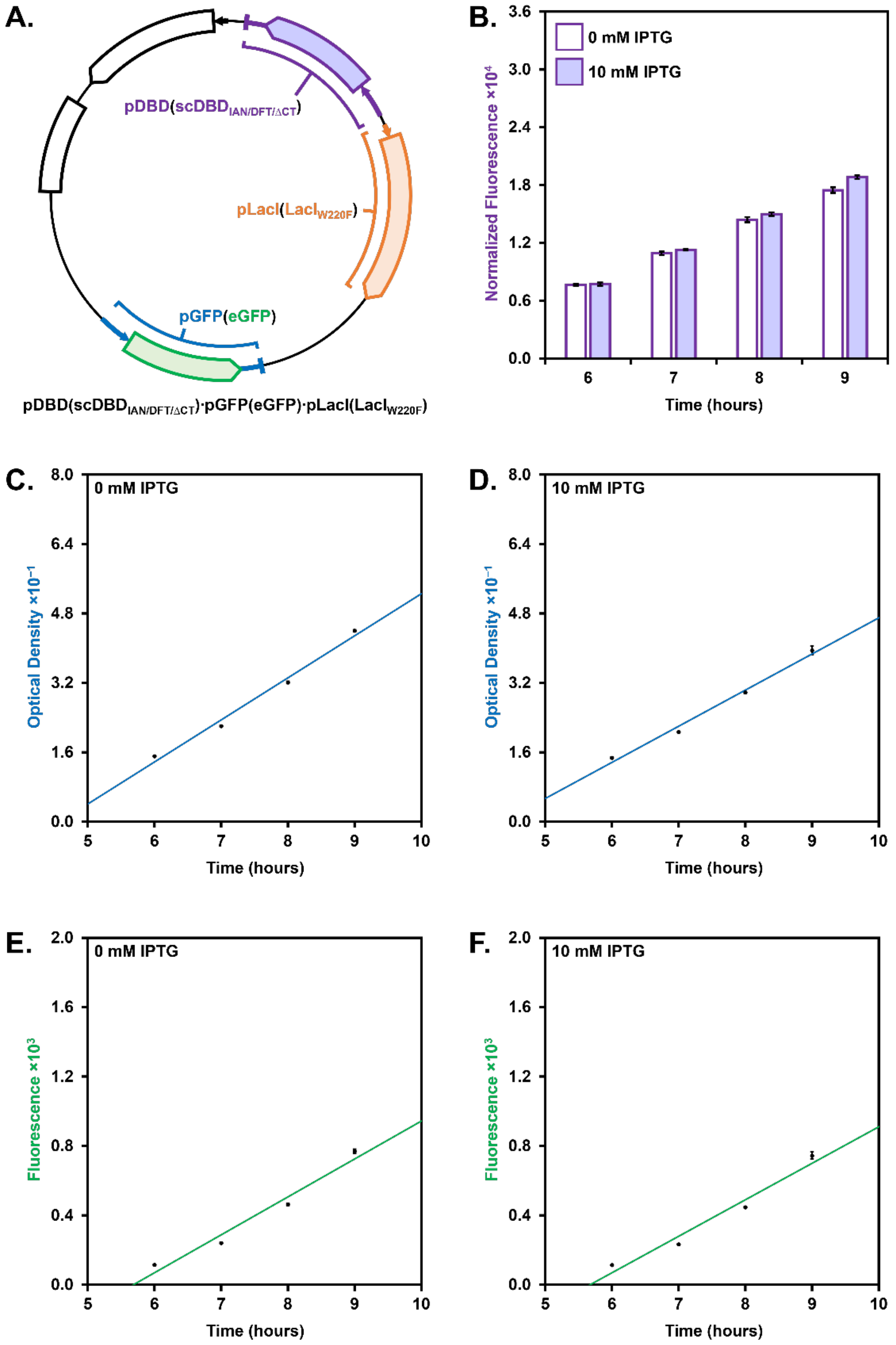

S20 Fig. Expression plots for the pDBD(scDBD$_{IAN/DFT/\Delta CT}$)·pGFP(eGFP)·pLacI(LacI$_{W220F}$) genetic circuit. Data includes the plasmid map for the genetic circuit (A) and time-course summary for the normalized fluorescence from the genetic circuit (B) with *p*-values indicated for statistically significant (*p*-value < 0.001, two-tailed homoscedastic *t*-test, replicates = 4) changes in output signal. Plots include culture density (*y*-axis) as a function of growth time (*x*-axis) in the absence (C) and presence (D) of 10 mM IPTG, and culture fluorescence (*y*-axis) as a function of growth time (*x*-axis) in the absence (E) and presence (F) of 10 mM IPTG.

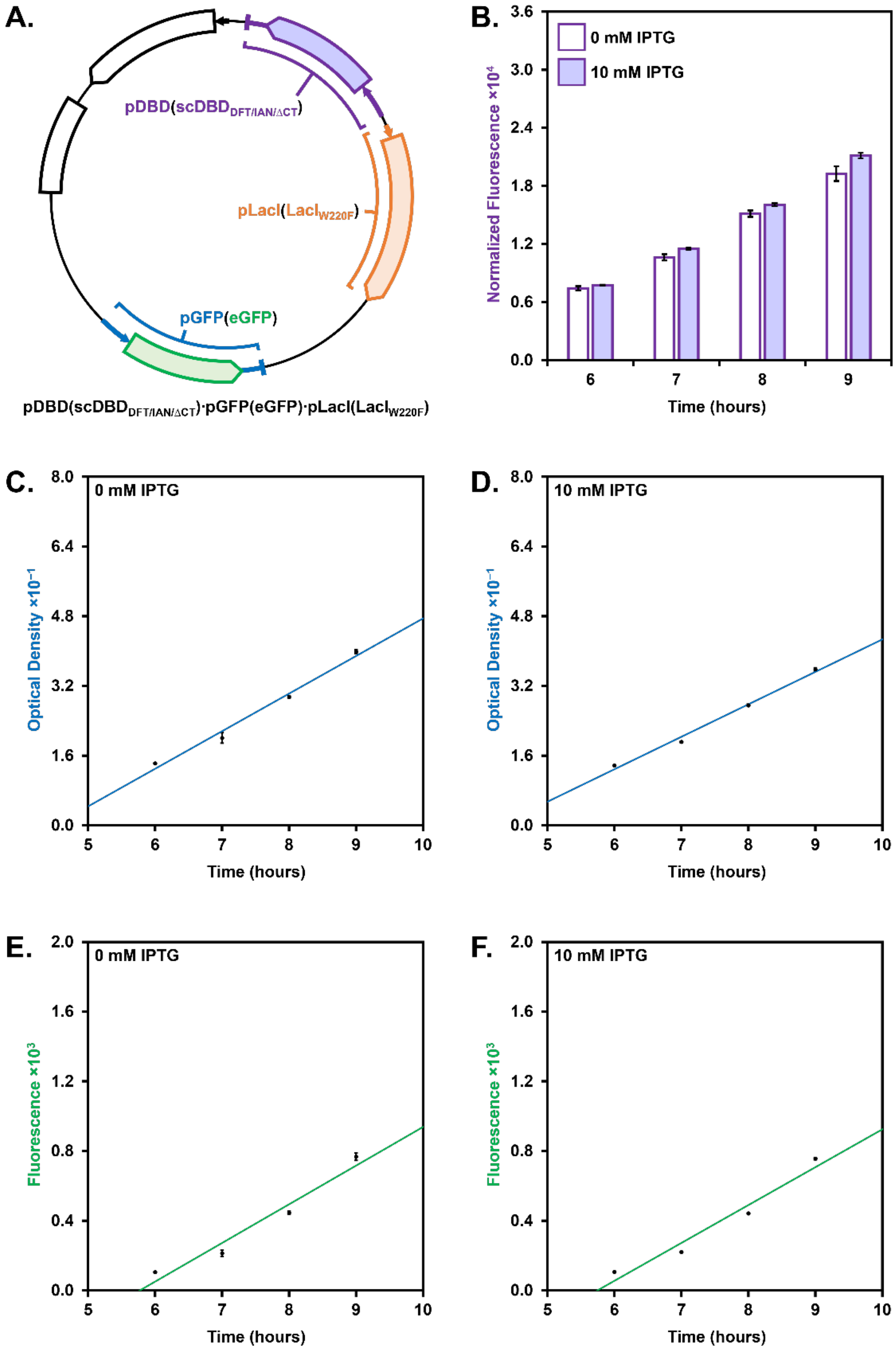

S21 Fig. Expression plots for the pDBD(scDBD$_{DFT/IAN/\Delta CT}$)·pGFP(eGFP)·pLacI(LacI$_{W220F}$) genetic circuit. Data includes the plasmid map for the genetic circuit (A) and time-course summary for the normalized fluorescence from the genetic circuit (B) with *p*-values indicated for statistically significant (p-value < 0.001, two-tailed homoscedastic *t*-test, replicates = 4) changes in output signal. Plots include culture density (*y*-axis) as a function of growth time (*x*-axis) in the absence (C) and presence (D) of 10 mM IPTG, and culture fluorescence (*y*-axis) as a function of growth time (*x*-axis) in the absence (E) and presence (F) of 10 mM IPTG.

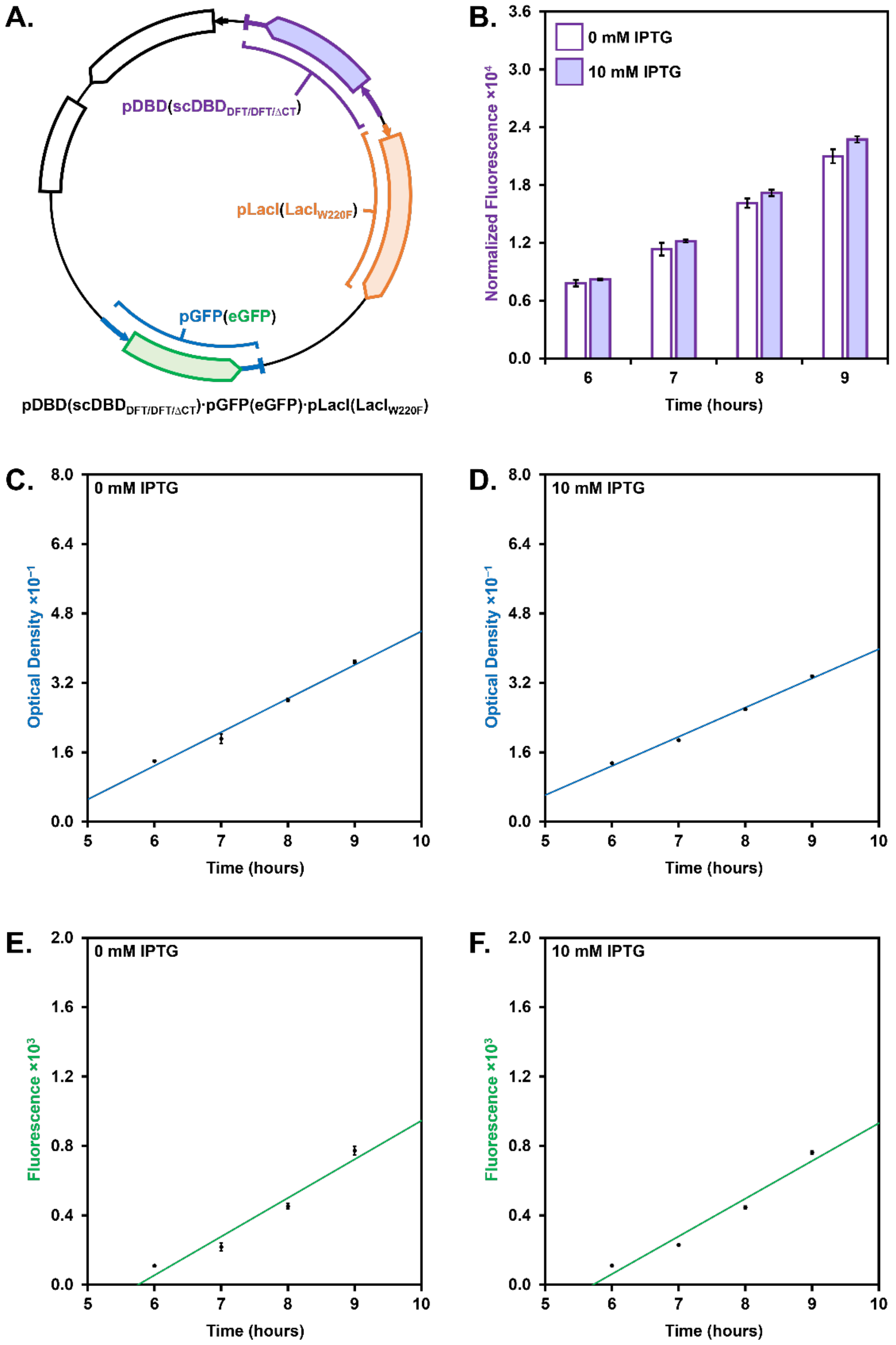

S22 Fig. Expression plots for the pDBD(scDBD$_{DFT/DFT/\Delta CT}$)·pGFP(eGFP)·pLacI(LacI$_{W220F}$) genetic circuit. Data includes the plasmid map for the genetic circuit (A) and time-course summary for the normalized fluorescence from the genetic circuit (B) with *p*-values indicated for statistically significant (*p*-value < 0.001, two-tailed homoscedastic *t*-test, replicates = 4) changes in output signal. Plots include culture density (*y*-axis) as a function of growth time (*x*-axis) in the absence (C) and presence (D) of 10 mM IPTG, and culture fluorescence (*y*-axis) as a function of growth time (*x*-axis) in the absence (E) and presence (F) of 10 mM IPTG.

S10 Table. Regression analysis of expression plots[A] for characterization of scDBD repressor function

scDBD Architecture			0 mM IPTG Culture			10 mM IPTG Culture		
**DBD.N**	**DBD.C**	**C-term**	**R^2**	**Slope**	**Intercept**	**R^2**	**Slope**	**Intercept**
***y*-axis: Optical Density (λ_{OD} = 600 nm) *as a function of x*-axis: Time (hours) $\times 10^{-1}$**								
IAN	IAN		0.981	0.86 ± 0.09	−3.84 ± 0.65	0.983	1.30 ± 0.12	−5.77 ± 0.90
IAN	DFT		0.981	0.93 ± 0.09	−4.25 ± 0.70	0.989	0.86 ± 0.06	−3.78 ± 0.49
DFT	IAN		0.988	0.87 ± 0.07	−3.86 ± 0.52	0.995	0.79 ± 0.04	−3.34 ± 0.31
DFT	DFT		0.993	0.83 ± 0.05	−3.50 ± 0.37	0.970	0.72 ± 0.09	−2.81 ± 0.68
IAN	IAN	ΔCT	0.982	0.87 ± 0.08	−3.92 ± 0.64	0.990	1.30 ± 0.09	−5.75 ± 0.71
IAN	DFT	ΔCT	0.987	0.97 ± 0.08	−4.44 ± 0.60	0.989	0.83 ± 0.06	−3.64 ± 0.47
DFT	IAN	ΔCT	0.985	0.86 ± 0.07	−3.88 ± 0.57	0.991	0.75 ± 0.05	−3.18 ± 0.38
DFT	DFT	ΔCT	0.987	0.78 ± 0.06	−3.36 ± 0.49	0.994	0.67 ± 0.04	−2.76 ± 0.28
***y*-axis: Fluorescence (λ_{ex} = 485 nm, λ_{em} = 510 nm) *as a function of x*-axis: Time (hours) $\times 10^{3}$**								
IAN	IAN		0.953	2.38 ± 0.37	−13.6 ± 2.8	0.957	0.65 ± 0.10	−3.44 ± 0.74
IAN	DFT		0.962	2.21 ± 0.31	−12.6 ± 2.4	0.963	2.13 ± 0.21	−12.1 ± 2.24
DFT	IAN		0.959	2.33 ± 0.34	−13.4 ± 2.6	0.960	2.33 ± 0.34	−13.3 ± 2.55
DFT	DFT		0.966	2.59 ± 0.35	−14.8 ± 2.6	0.947	2.52 ± 0.42	−14.5 ± 3.20
IAN	IAN	ΔCT	0.956	2.23 ± 0.34	−12.9 ± 2.6	0.935	0.85 ± 0.16	−4.77 ± 1.20
IAN	DFT	ΔCT	0.967	2.19 ± 0.29	−12.4 ± 2.2	0.966	2.10 ± 0.28	−11.9 ± 2.13
DFT	IAN	ΔCT	0.955	2.22 ± 0.34	−12.8 ± 2.6	0.959	2.17 ± 0.32	−12.5 ± 2.40
DFT	DFT	ΔCT	0.956	2.22 ± 0.34	−12.8 ± 2.6	0.960	2.17 ± 0.31	−12.4 ± 2.38
***y*-axis: Fluorescence (λ_{ex} = 485 nm, λ_{em} = 510 nm) *as a function of x*-axis: Optical Density (λ_{OD} = 600 nm) $\times 10^{4}$**								
IAN	IAN		0.994	2.79 ± 0.15	−0.31 ± 0.04	0.921	0.49 ± 0.10	−0.05 ± 0.04
IAN	DFT		0.997	2.38 ± 0.10	−0.26 ± 0.03	0.992	2.50 ± 0.16	−0.28 ± 0.04
DFT	IAN		0.991	2.71 ± 0.18	−0.31 ± 0.05	0.983	2.98 ± 0.28	−0.36 ± 0.08
DFT	DFT		0.989	3.14 ± 0.24	−0.40 ± 0.07	0.988	3.54 ± 0.28	−0.47 ± 0.08
IAN	IAN	ΔCT	0.994	2.60 ± 0.14	−0.29 ± 0.04	0.904	0.64 ± 0.15	−0.09 ± 0.06
IAN	DFT	ΔCT	0.995	2.27 ± 0.11	−0.25 ± 0.03	0.992	2.54 ± 0.16	−0.28 ± 0.05
DFT	IAN	ΔCT	0.991	2.60 ± 0.18	−0.29 ± 0.05	0.985	2.94 ± 0.26	−0.33 ± 0.07
DFT	DFT	ΔCT	0.988	2.90 ± 0.23	−0.32 ± 0.06	0.984	3.26 ± 0.29	−0.36 ± 0.07

[A]Data collected at 6-, 7-, 8-, and 9-hour time-points in quadruplicate (n = 4) as shown in panels C – F for S14 – S21 Figs. Note that triple mutation IAN (I17/A18/N22) is required for DBD recognition of the pGFP promoter and triple mutation DFT (D17/F18/T22) renders the DBD incapable of repressing the pGFP promoter.

Section 6. References for Supplementary Information

1. Cormack BP, Valdivia RH, Falkow S. FACS-optimized mutants of the green fluorescent protein (GFP). Gene 1996;173: 33−38.
2. Milk L, Daber R, Lewis M. Functional reules for lac repressor-operator associations and implications for protein-DNA interactions. Protein Sci. 2010;19: 1162−1172.
3. Cox RS III, Surette MG, Elowitz MB. Programming gene expression with combinatorial promoters. Mol Syst Biol. 2007;3: 145−155.
4. Garcia HG, Sanchez A, Boedicker JQ, Osborne M, Gelles J, Kondev J, Phillips R. Operator sequence alters gene expression independently of transcription factor occupancy in bacteria. Cell Rep. 2012;2: 150−161.
5. Barondeau DP, Putnam CD, Kassmann CJ, Tainer JA, Getzoff ED. Mechanism and energetics of green fluorescent protein chromophore synthesis revealed by trapped intermediate structures. Proc Natl Acad Sci USA. 2003;100: 12111−12116.
6. Davey JA, Wilson CJ. Engineered signal-coupled inducible promoters: measuring the apparent RNA-polymerase resource budget. Nucleic Acids Res. 2020;48: 9995−10012.
7. Gatii-Lefranconi P, Dijkman WP, Devenish SRA, Hollfelder F. A single mutation in the core domain of the lac repressor reduces leakiness. Microb Cell Fact. 2013;12: 67−76.
8. Calos MP. DNA sequence for a low-level promoter of the lac repressor gene and an 'up' promoter mutation. Nature 1978; 274: 762−765.
9. Hawley TS, Hawley RG, Telford WG. Fluorescent proteins for flow cytometry. Curr Protoc Cytom 2017;80: 9.12.1−0.12.20.
10. Rantasalo A, Kuivanen J, Penttilä M, Jäntti J, Mojzita D. Synthethic toolkit for complex genetic circuit engineering in *Sacchromyces cerevisiae*. ACS Synth Biol. 2018;7: 1573−1587.
11. Shis DL, Hussain F, Meinhardt S, Swint-Kruse L, Bennett MR. Modular, multi-input transcriptional logic gating with orthogonal LacI/GalR family chimeras. ACS Synth Biol. 2014;3: 645−651.
12. Cuccato G, Gatta GD, di Bernardo D. Systems and synthetic biology: tackling genetic networks and complex diseases. Heredity 2009;102: 527−532.
