# Supplementary material for: TReSR: A PCR-compatible DNA sequence design method for engineering proteins containing tandem repeats
Source: PLoS One. 2023 Apr 12;18(4):e0281228. doi: 10.1371/journal.pone.0281228 (PMC10096509; doi:10.1371/journal.pone.0281228)
Supplement: Attachment — Submitted filename: Response_to_reviewers.pdf [file pone.0281228.s003.pdf]

Response to Reviewers

We appreciate the insightful feedback that was provided by both reviewers and have put in our best effort to address all concerns that were raised. In the text below we have included a copy of each comment followed by an explanation of how it was addressed in the revised document.

Reviewer 1. Comment 1. Line 34. The authors state that TR motifs are "unexploited" as a class of molecular components. In fact, there are many types of repeat proteins that have been engineered, including some for commercial purposes (e.g. DARPins and TAL effectors). Maybe the word could be changed to "underexploited," though even that would be debatable. A similar overstatement is when they say manipulation of TR DNA sequences by PCR is currently "prohibitive" (line 555).

Response. We agree with the reviewer's recommendation to change "TR motifs are unexploited as a class of molecular components" (Line 34 in first version of manuscript) by substituting "unexploited" for "underexploited". We have also amended the manuscript to soften the statement about 'prohibitive' sequences by making the following changes (indicated in italics);
(Line 58 in updated version) "Despite their utility and abundance, TR sequence motifs remain *underexploited* as a class of modular components for the purposes of protein and genetic circuit engineering precisely because they are encoded by repetitive DNA sequences *thereby complicating* the routine application of PCR-based molecular biology techniques"
(Line 803 in updated version) "*The ability to improve the relative ease of* PCR *based* synthesis and manipulation of TR DNA sequences *can alleviate potential experimental complications* interfering with the routine *utilization* of TR sequences *for protein engineering applications.*"
Because Reviewer 1 has also provided us with two key examples of commercially employed TR sequences, we have opted to include DARPins and TAL effectors as specific examples of TR sequences when introducing the topic in the manuscript:
(Line 55 in updated version) "*The functional modularity of TR sequence motifs has been exploited in the development of new biotechnologies such as DARPins [PMID: 25562645] and TAL effectors [PMID: 23265998][PMID: 21960622] that allow engineering of specific protein and DNA binding functionality, respectively.*"

Reviewer 1. Comment 2. The experimental demonstration is fairly limited, just making a single repeat protein of only two units, where many repeat proteins have quite a bit more than two units. Why weren't known functional repeat proteins like DARPins or TAL effectors engineered to demonstrate the design algorithm works for more than just two-unit repeat proteins? As such, I think the authors can only claim their algorithm works for making two-unit repeat proteins.

Response. The reviewer correctly notes that our experimental demonstration of the TReSR algorithm is applied to a single example TR protein produced by duplicating the LacI DNA binding domain. This target was specifically selected for a separate project being developed in our lab to investigate whether the LacI DNA binding domain is a modular component that can also be compatibly incorporated into a TR construct, thereby expanding the library of repressor components which could be utilized when engineering sequence-specific repressors. The difficulties that prevented PCR synthesis and manipulation of these constructs led us to develop TReSR, the tool we are presenting in this manuscript in hopes that it might be helpful to fellow protein scientists encountering similar difficulties. Our demonstration of TReSR's utility required assembly PCR synthesis and functional evaluation of this new TR repressor protein, and the engineering of a new reporter genetic circuit to evaluate its function. Presentation of genetic circuit design and controls, in addition to TR repressor design, construction and optimization gave rise to the current form of our manuscript that consists of 25 main text and 56 supplementary text pages.

We therefore had concerns that the inclusion of design, synthesis, and function data for additional TR scaffolds would give rise to a more complicated story that would obscure the main purpose of the manuscript, which is to present the development and application of our algorithm. Since we agree with the reviewer's observation that our demonstration did not expand beyond our chosen target, we have made the following changes to the manuscript (indicated in italics);

(Lines 479 old version) Replace:

"The use of TReSR to create TR-containing proteins with DNA sequences that allow aPCR assembly and PCR-based manipulation opens the door to simultaneous screening of more than one module and increases the range of TR-containing proteins that can be designed." with;

(Line 688, updated version) " The *application* of TReSR to create *a new TR-containing protein from the duplication of the LacI DNA binding domain* by aPCR *demonstrates the simultaneous synthesis and* screening of *protein constructs incorporating duplicate pairs of modular-TR components. This strategy could be applied to similar protein engineering problems that investigate the scope of modular protein domains hypothesized to be compatible and interchangeable components in TR constructs*."

(Line 483 of first version) Replace:

"It is expected that our DNA sequence redesign strategy will be able to successfully accommodate the duplication of at least three domain-length sequences into a single construct, since it was possible to perform site-selective PCR-based manipulation of a single DBD in a plasmid that contained DBD sequences from both native LacI and the scDBD construct." with;

(Line 703, updated version) "While our DNA sequence redesign strategy was successfully applied to the generation of a protein containing *two* domain-length sequences, we anticipate that TReSR should be capable of generating proteins with three repeated domains since it was possible to perform site-selective PCR-based manipulation of a single DBD domain in a plasmid that contained a total of three DBD sequence copies, including the native LacI sequence and the scDBD construct".

Reviewer 1. Comment 3. Changing of codons can result in changes of expression. The authors show that their construct achieves only about 6-fold repression (my estimate based on Fig 4BC). A missing essential control is how much a construct with an exact repeat of the wildtype sequence would repress (note that there are a lot of control experiments in the supplementary, but I had a hard time understanding the authors' nomenclature and I think all these control experiments were performed with tandem repeats that were not repetitive in the DNA sequence). I would expect it to be much more that 6-fold based on the typical properties of the lac repressor, but I don't know for sure. One reason for poor performance would be poor translation due to the DNA sequence (i.e. non-optimal codons or regions of the gene). Although they say codon usage information can be incorporated into their method, they did not do so in this study (even so, codon optimization for expression is much more complicated than just choosing the often-used codons for that organism). If the authors algorithm produces a tandem duplicate gene that has poor expression, that is a caveat to the authors' method. But if the authors construct represses about as well as the non-optimized gene, then the authors method would be more useful.

Response. The reviewer anticipates that when the LacI DNA binding domain is duplicated to produce the new single-chain tandem repeat construct (scDBD) presented in this study, this scDBD construct should exhibit a repressor function that exceeds the observed six-fold decrease in reporter transcription reported (Fig 4BC). We agree with the reviewer that the use of synonymous codons to duplicate our scDBD construct will affect the translation efficiency of the scDBD construct and have provided discussion in the manuscript regarding this point (see Lines 739 – 745 of updated manuscript). However, we disagree with the expectation that scDBD repressor performance should be directly comparable to the native LacI scaffold given that:

1. the lac repressor exerts control over gene transcription by assembling as a functional homodimer while our scDBD is demonstrated to function as a single-chain construct (Fig 4B),
2. LacI and all its related family members have N-terminal arranged DNA binding domains while our scDBD construct functions as with a contiguous sequence by presenting a pair of DNA binding domains arranged from N- to C-terminal,
3. considerable experimental evidence has been collected demonstrating that the repression activity of the Lac repressor is highly sensitive to linker residue mutations, referred to rheostat positions [please see: Meinhardt *et al*., *PLoS ONE*, 2013, 8, e83502, PMID: 24386217] that alter the placement and affinity of the DNA binding domain for the operator DNA sequence,
4. the non-cognate DNA binding domain (Y17I/Q18A/R22N) belonging to the functional scDBD construct and its target DNA operator (G4T/G6A) driving expression of the eGFP reporting protein for the genetic circuit have different binding affinities and repression strengths compared to their native (cognate) counterpart [please see: Davey JA & Wilson CJ, *Nucleic Acids Research*, 2020, 48, 9995, PMID: 32890400], and
5. the performance of our genetic circuit controls (Section 4 of the SI text) suggests that the expression output from the pDBD promoter at Cloning Site I (controlling expression of the experimental scDBD construct) is well in excess of the plasmid copy number (estimated to have only 15 to 20 copies per cell).

Therefore, while the 6-fold level of suppression is lower than the levels obtained with the native LacI dimer, thanks to the development of our TReSR algorithm we have since been able to pursue functional studies of our scDBD constructs to engineer repressor specificity and activity. The results of that study, which involve mutagenesis of five repressor architectures with twelve different DNA binding domain triple mutations, is beyond the scope of the current manuscript which we have designed to remain focussed on the development and application of TReSR. Instead, our results on the scDNA family of engineered repressors will be presented in a separate paper since that contributes new repressors to the synthetic biology toolbox.

We agree with Reviewer 1 that the lower observed level of repression activity measured for our first generation scDBD construct should be noted in the manuscript, and so we have amended the discussion to include a short paragraph that addresses this point at lines 694 to 701 of the updated manuscript:

"Evaluation of repression activity reported using our genetic circuit demonstrates that the action of our *first*-generation scDBD TR construct on the pGFP promoter exerts an approximate 6-fold decrease in expression output relative to the unrepressed pGFP promotor. Comparison of the modest repression activity exhibited by scDBD with results from mutagenesis studies of rheostat positions [PMID: 32780837] identified in the linker sequence of LacI [PMID: 24386217] suggests suboptimal arrangement and association of the scDBD DNA binding domain pair with the target DNA operator sequence. It is likely that future mutagenesis studies, made possible by the application of our TReSR algorithm, may produce TR constructs with increased repressor activities by optimizing the DNA binding domain pair arrangement to improve scDBD association with its DNA operator."

Reviewer 1. Comment 4. Line 242. ABS is not the best abbreviation for what is being measured. What is being measured is optical density (OD) not absorbance (i.e. the physical phenomena of why the measurement increases with cell density is light scattering, not absorbance).

Response. The reviewer is correct to note that the culture density is monitored by measuring the scattering of light. All in-text references, figures, and tables have been amended to correct this error

(removed text indicated by strike-through font and inserted replacement text indicated with underline font).

(Original manuscript Line 197) "Evaluation of in vivo reporter protein expression was performed using a SpectraMax M2 plate reader (Molecular Devices) to record ~~absorbance~~optical density (~~λ_{ABS}~~λ_{OD} = 600 nm) and fluorescence (λ_{ex} = 485 nm, λ_{em} = 510 nm, fixed gain = medium, 30 flashes per read) from 100 µL aliquots of cell culture in black-walled and clear-bottomed 96-well format microplates (Greiner)."

(Original manuscript Line 234) "The resulting culture plate setup allows for twelve separate transformants to be grown in quadruplicate at a 0.6 mL culture volume in the presence and absence of 10 mM IPTG with a starting density of 0.005 ~~absorbance~~ units (~~λ_{ABS}~~λ_{OD} = 600 nm)."

(Original manuscript Line 238) "Linear regression analysis of culture density (Y: ~~λ_{ABS}~~λ_{OD} = 600 nm) as a function of time (X: hours), fluorescence (Y: λ_{ex} = 485 nm, λ_{em} = 510 nm, gain = medium, 30 flashes per read) as a function of time (X: hours), and fluorescence (Y: λ_{ex} = 485 nm, λ_{em} = 510 nm, gain = medium, 30 flashes per read) as a function of ~~absorbance~~optical density (X: ~~λ_{ABS}~~λ_{OD} = 600 nm) demonstrate that all measurements, regardless of absence or presence of 10 mM IPTG, were linear and recorded at steady-state (Figures S13−S20)."

(Original manuscript Line 243) "Genetic circuit expression output (F) is reported by taking the quotient of fluorescence (GFP) and density (~~ABS~~OD) from each culture measurement (Equation 1)."

(Original manuscript Line 245) "$F = \frac{GFP}{OD}$"

(Original manuscript Figure 4B and 4C) Y-axis now reads: "Normalized Fluorescence"

(SI pg 26 ¶ 2) "The output signal (*F*) for any given genetic circuit is quantified as the culture fluorescence (~~GFP~~*GFP*, λ_{ex} = 485 nm, λ_{em} = 510 nm, gain = medium, 30 flashes per read) ~~density~~, normalized by ~~cell~~ culture density, as measured by ~~absorbance~~ optical density (~~ABS~~*OD*, ~~λ_{ab}~~λ_{OD} = 600 nm), (Equation S1)."

(SI pg 26 Equation S1) "$F = \frac{GFP}{\sout{ABS}OD}$"

(SI pg 26 ¶ 5) "As shown in Figures S6D – S6F and S8D – S8F (data in Tables S4 and S6, respectively) at non-inducing concentrations of IPTG (*i.e.,* < 10 [illegible]M), ~~absorbance~~density-normalized fluorescence was very low across all time-points tested (*i.e.,* 7, 8 and 9-hours)."

(SI pg 27 ¶ 3) "However, growth in the presence of inducing concentrations of IPTG (*e.g.,* >1 mM) showed significantly reduced ~~absorbance~~ normalized fluorescence for both pLacI^{Q} constructs compared to their counterparts with pLacI. For example, induction of pLacI^{Q}(LacI) and pLacI^{Q}(LacI$_{W220F}$) genetic circuits with 10 mM IPTG produced 506 ± 7 and 613 ± 6 density-normalized ~~culture~~ fluorescence units, having only 1.35 ± 0.03 and 1.66 ± 0.03 fold gain over baseline expression output (1 µM IPTG), respectively."

(SI pg 28 ¶ 4) "For this reason, the culture density, approximated by ~~absorbance~~optical density (*OD*), and fluorescence output (GFP) were plotted as a function of time using samples taken at 6-, 7-, 8-, and 9-hour time-points for each genetic circuit (Fig S6-9, panels B and C)."

(SI pg 28 ¶ 4) "All of the pDBD(eGFP)·pGFP(dGFP) genetic circuits exhibited linear relationships in these plots, with R^2 correlating ~~absorbance~~optical density as a function of time calculated between 0.952 and 1.000, and R^2 correlating fluorescence as a function of time between 0.805 and 0.999."

(SI pg 30 Table S4) "**~~Absorbance~~Optical Density ×10^{-1} (~~λ_{ABS}~~λ_{OD} = 600 nm)^{b}**"

(SI pg 30 Table S4) "**Normalized Fluorescence ~~÷ Absorbance~~×10^{3} (λ_{ex} = 485 nm, λ_{em} = 510 nm, ~~λ_{ABS}~~λ_{OD} = 600 nm)^{d}**"

(SI pg 30 Table S4) “[b]linear regression analysis performed for ~~absorbance~~optical density (*y*-axis) as a function of time (*x*-axis) plotted in Figure S6B”

(SI pg 30 Table S4) “[d]linear regression analysis performed for fluorescence (*y*-axis) as a function of ~~absorbance~~optical density (*x*-axis)”

(SI pg 31 Figure S6B) “**~~Absorbance~~Optical Density $\times 10^{-1}$**”

(SI pg 31 Figure S6D, S6E, S6F) “**Normalized Fluorescence ~~÷ Absorbance~~$\times 10^{4}$**”

(SI pg 31 Figure S6 Caption) “Data includes the plasmid map for the genetic circuit (A), plot of ~~absorbance~~ optical density at 600 nm (B), and culture fluorescence at 510 nm (λ_{ex} = 485 nm) (C), as a function of growth time over the range of IPTG concentrations indicated by the colour scheme, and plots of ~~cell density~~ normalized fluorescence as a function of inducer concentration for 7- (D), 8- (E), and 9- (F) hour time-points”

(SI pg 32 Table S5) “**~~Absorbance~~Optical Density $\times 10^{-1}$ (~~λ_{ABS}~~λ_{OD} = 600 nm)[b]**”

(SI pg 32 Table S5) “**Normalized Fluorescence ~~÷ Absorbance~~$\times 10^{2}$ (λ_{ex} = 485 nm, λ_{em} = 510 nm, ~~λ_{ABS}~~λ_{OD} = 600 nm)[d]**”

(SI pg 32 Table S5) “[b]linear regression analysis performed for ~~absorbance~~optical density (*y*-axis) as a function of time (*x*-axis) plotted in Figure S7B”

(SI pg 32 Table S5) “[d]linear regression analysis performed for fluorescence (*y*-axis) as a function of ~~absorbance~~optical density (*x*-axis)”

(SI pg 33 Figure S7B) “**~~Absorbance~~Optical Density $\times 10^{-1}$**”

(SI pg 33 Figure S7D, S7E, S7F) “**Normalized Fluorescence ~~÷ Absorbance~~$\times 10^{2}$**”

(SI pg 33 Figure S7 Caption) “Data includes the plasmid map for the genetic circuit (A), plot of ~~absorbance~~ optical density at 600 nm (B), and culture fluorescence at 510 nm (λ_{ex} = 485 nm) (C), as a function of growth time over the range of IPTG concentrations indicated by the colour scheme, and plots of ~~cell density~~ normalized fluorescence as a function of inducer concentration for 7- (D), 8- (E), and 9- (F) hour time-points”

(SI pg 34 Table S6) “**~~Absorbance~~Optical Density $\times 10^{-1}$ (~~λ_{ABS}~~λ_{OD} = 600 nm)[b]**”

(SI pg 34 Table S6) “**Normalized Fluorescence ~~÷ Absorbance~~$\times 10^{3}$ (λ_{ex} = 485 nm, λ_{em} = 510 nm, ~~λ_{ABS}~~λ_{OD} = 600 nm)[d]**”

(SI pg 34 Table S6) “[b]linear regression analysis performed for ~~absorbance~~optical density (*y*-axis) as a function of time (*x*-axis) plotted in Figure S8B”

(SI pg 34 Table S6) “[d]linear regression analysis performed for fluorescence (*y*-axis) as a function of ~~absorbance~~optical density (*x*-axis)”

(SI pg 35 Figure S8B) “**~~Absorbance~~Optical Density $\times 10^{-1}$**”

(SI pg 35 Figure S8D, S8E, S8F) “**Normalized Fluorescence ~~÷ Absorbance~~$\times 10^{4}$**”

(SI pg 35 Figure S8 Caption) “Data includes the plasmid map for the genetic circuit (A), plot of ~~absorbance~~ optical density at 600 nm (B), and culture fluorescence at 510 nm (λ_{ex} = 485 nm) (C), as a function of growth time over the range of IPTG concentrations indicated by the colour scheme, and plots of ~~cell density~~ normalized fluorescence as a function of inducer concentration for 7- (D), 8- (E), and 9- (F) hour time-points”

(SI pg 36 Table S7) "**~~Absorbance~~Optical Density ×10^{-1} (~~λ_{ABS}~~λ_{OD} = 600 nm)[b]**"

(SI pg 36 Table S7) "**Normalized Fluorescence ~~÷ Absorbance~~×10^{2} (λ_{ex} = 485 nm, λ_{em} = 510 nm, ~~λ_{ABS}~~λ_{OD} = 600 nm)[d]**"

(SI pg 36 Table S7) "[b]linear regression analysis performed for ~~absorbance~~optical density (*y*-axis) as a function of time (*x*-axis) plotted in Figure S9B"

(SI pg 36 Table S7) "[d]linear regression analysis performed for fluorescence (*y*-axis) as a function of ~~absorbance~~optical density (*x*-axis)"

(SI pg 37 Figure S9B) "**~~Absorbance~~Optical Density ×10^{-1}**"

(SI pg 37 Figure S9D, S9E, S9F) "**Normalized Fluorescence ~~÷ Absorbance~~×10^{2}**"

(SI pg 37 Figure S9 Caption) "Data includes the plasmid map for the genetic circuit (A), plot of ~~absorbance~~ optical density at 600 nm (B), and culture fluorescence at 510 nm (λ_{ex} = 485 nm) (C), as a function of growth time over the range of IPTG concentrations indicated by the colour scheme, and plots of ~~cell density~~ normalized fluorescence as a function of inducer concentration for 7- (D), 8- (E), and 9- (F) hour time-points"

(SI pg 40 ¶ 5) "Linear regression analysis was performed for the set of ~~absorbance~~optical density and fluorescence outputs from each of the four control genetic circuits, demonstrating linear (*i.e.,* steady-state) correlations as a function of time (Table S9)."

(SI pg 40 ¶ 5) "Specifically, linear regression reveals strong time-dependent correlations for both ~~absorbance~~optical density (R^2 = 0.983 − 0.995) and fluorescence (R^2 = 0.845 − 0.996), irrespective of the presence of inducer."

(SI pg 41 Figure S10B) "**Normalized Fluorescence ~~÷ Absorbance~~×10^{4}**"

(SI pg 41 Figure S10C, S10D) "**~~Absorbance~~Optical Density ×10^{-1}**"

(SI pg 41 Figure S10 Caption) "Data includes the plasmid map for the genetic circuit (A) and time-course summary for the ~~absorbance-~~normalized fluorescence from the genetic circuit (B) with p-values indicated for statistically significant (p-value < 0.001) changes in output signal."

(SI pg 42 Figure S11B) "**Normalized Fluorescence ~~÷ Absorbance~~×10^{4}**"

(SI pg 42 Figure S11C, S11D) "**~~Absorbance~~Optical Density ×10^{-1}**"

(SI pg 42 Figure S11 Caption) "Data includes the plasmid map for the genetic circuit (A) and time-course summary for the ~~absorbance-~~normalized fluorescence from the genetic circuit (B) with p-values indicated for statistically significant (p-value < 0.001) changes in output signal."

(SI pg 43 Figure S12B) "**Normalized Fluorescence ~~÷ Absorbance~~×10^{4}**"

(SI pg 43 Figure S12C, S12D) "**~~Absorbance~~Optical Density ×10^{-1}**"

(SI pg 43 Figure S12 Caption) "Data includes the plasmid map for the genetic circuit (A) and time-course summary for the ~~absorbance-~~normalized fluorescence from the genetic circuit (B) with p-values indicated for statistically significant (p-value < 0.001) changes in output signal."

(SI pg 44 Figure S13B) "**Normalized Fluorescence ~~÷ Absorbance~~×10^{4}**"

(SI pg 44 Figure S13C, S13D) "**~~Absorbance~~Optical Density ×10^{-1}**"

(SI pg 44 Figure S13 Caption) "Data includes the plasmid map for the genetic circuit (A) and time-course summary for the ~~absorbance-~~normalized fluorescence from the genetic circuit (B) with p-values indicated for statistically significant (p-value < 0.001) changes in output signal."

(SI pg 45 Table S9) "**Optical Density~~Absorbance~~ vs Time[c,d] (hours)**"

(SI pg 45 Table S9) "**Fluorescence vs Optical Density~~Absorbance~~**"

(SI pg 45 Figure S14) "**Normalized Fluorescence ~~÷ Absorbance~~$\times 10^{4}$**"

(SI pg 47 Figure S15B) "**Normalized Fluorescence ~~÷ Absorbance~~$\times 10^{4}$**"

(SI pg 47 Figure S15C, S15D) "**~~Absorbance~~Optical Density $\times 10^{-1}$**"

(SI pg 47 Figure S15 Caption) "Data includes the plasmid map for the genetic circuit (A) and time-course summary for the ~~absorbance-~~normalized fluorescence from the genetic circuit (B) with p-values indicated for statistically significant (p-value < 0.001) changes in output signal."

(SI pg 48 Figure S16B) "**Normalized Fluorescence ~~÷ Absorbance~~$\times 10^{4}$**"

(SI pg 48 Figure S16C, S16D) "**~~Absorbance~~Optical Density $\times 10^{-1}$**"

(SI pg 48 Figure S16 Caption) "Data includes the plasmid map for the genetic circuit (A) and time-course summary for the ~~absorbance-~~normalized fluorescence from the genetic circuit (B) with p-values indicated for statistically significant (p-value < 0.001) changes in output signal."

(SI pg 49 Figure S17B) "**Normalized Fluorescence ~~÷ Absorbance~~$\times 10^{4}$**"

(SI pg 49 Figure S17C, S17D) "**~~Absorbance~~Optical Density $\times 10^{-1}$**"

(SI pg 49 Figure S17 Caption) "Data includes the plasmid map for the genetic circuit (A) and time-course summary for the ~~absorbance-~~normalized fluorescence from the genetic circuit (B) with p-values indicated for statistically significant (p-value < 0.001) changes in output signal."

(SI pg 50 Figure S18B) "**Normalized Fluorescence ~~÷ Absorbance~~$\times 10^{4}$**"

(SI pg 50 Figure S18C, S18D) "**~~Absorbance~~Optical Density $\times 10^{-1}$**"

(SI pg 50 Figure S18 Caption) "Data includes the plasmid map for the genetic circuit (A) and time-course summary for the ~~absorbance-~~normalized fluorescence from the genetic circuit (B) with p-values indicated for statistically significant (p-value < 0.001) changes in output signal."

(SI pg 51 Figure S19B) "**Normalized Fluorescence ~~÷ Absorbance~~$\times 10^{4}$**"

(SI pg 51 Figure S19C, S19D) "**~~Absorbance~~Optical Density $\times 10^{-1}$**"

(SI pg 51 Figure S19 Caption) "Data includes the plasmid map for the genetic circuit (A) and time-course summary for the ~~absorbance-~~normalized fluorescence from the genetic circuit (B) with p-values indicated for statistically significant (p-value < 0.001) changes in output signal."

(SI pg 52 Figure S20B) "**Normalized Fluorescence ~~÷ Absorbance~~$\times 10^{4}$**"

(SI pg 52 Figure S20C, S20D) "**~~Absorbance~~Optical Density $\times 10^{-1}$**"

(SI pg 52 Figure S20 Caption) "Data includes the plasmid map for the genetic circuit (A) and time-course summary for the ~~absorbance-~~normalized fluorescence from the genetic circuit (B) with p-values indicated for statistically significant (p-value < 0.001) changes in output signal."

(SI pg 53 Figure S21B) "**Normalized Fluorescence ~~÷ Absorbance~~$\times 10^{4}$**"

(SI pg 53 Figure S21C, S21D) "~~**Absorbance**~~**Optical Density** **×10^{-1}**"

(SI pg 53 Figure S21 Caption) "Data includes the plasmid map for the genetic circuit (A) and time-course summary for the ~~absorbance~~ normalized fluorescence from the genetic circuit (B) with p-values indicated for statistically significant (p-value < 0.001) changes in output signal."

(SI pg 54 Figure S22B) "**Normalized Fluorescence** ~~**÷ Absorbance**~~**×10^{4}**"

(SI pg 54 Figure S22C, S22D) "~~**Absorbance**~~**Optical Density** **×10^{-1}**"

(SI pg 54 Figure S22 Caption) "Data includes the plasmid map for the genetic circuit (A) and time-course summary for the ~~absorbance~~ normalized fluorescence from the genetic circuit (B) with p-values indicated for statistically significant (p-value < 0.001) changes in output signal."

(SI pg 55 Table S10) "***y*-axis: ~~Absorbance~~Optical Density (λ_{OD} = 600 nm) *as a function of x*-axis: Time (hours) ×10^{-1}**"

(SI pg 55 Table S10) **"*y*-axis: Fluorescence (λ_{ex} = 485 nm, λ_{em} = 510 nm) *as a function of x*-axis: ~~Absorbance~~Optical Density (λ_{OD} = 600 nm) ×10^{4}"**

Reviewer 1. Comment 5. Variables should be italicized in text (e.g. line 244 the variables in the equation should be italicized and line 247 t-test and p-value, the t and p should be italicized.)

Response. All in-text, figure, and table occurrences of "*p*-value","*t*-test", "*x*-axis", "*y*-axis", and associated sigmoid regression variables have been italicized.

Italicized instances of variable *F*: Original manuscript: Line 330, Line 332 (Equation 1), Supporting Information: SI pg 26 ¶ 2, SI pg 27 ¶ 5, SI pg 39 ¶ 2 (5-times), SI pg 39 ¶ 4 (twice), SI pg 40 ¶ 1 (5-times), SI pg 40 ¶ 3 (4-times), SI pg 40 ¶ 6 (6-times)

Italicized instances of variable *GFP*: Revised manuscript: Line 331, Line 332 (Equation 1), Supporting information: SI pg 26 ¶ 2, SI pg 26 Equation S1

Italicized instances of variable *OD*: Revised manuscript: Line 331, Line 332 (Equation 1), Supporting information: SI pg 26 ¶ 2, SI pg 26 Equation S1, SI pg 28 ¶ 4

Italicized instances of variable *p*-value: Revised manuscript lines 335, 336, and 663, Supporting information: SI pg 27 ¶ 1 (twice), SI pg 27 ¶ 3, SI pg 39 ¶ 2, SI pg 39 ¶ 4 (twice), SI pg 40 ¶ 1 (twice), SI pg 40 ¶ 3 (twice), SI pg 40 ¶ 6, SI pg 41 Figure S10 Caption (twice), SI pg 42 Figure S11 Caption (twice), SI pg 43 Figure S12 Caption (twice), SI pg 44 Figure S13 Caption (twice), SI pg 45 Figure S14 Caption (twice), SI pg 47 Figure S15 Caption (twice), SI pg 48 Figure S16 Caption (twice), SI pg 49 Figure S17 Caption (twice), SI pg 50 Figure S18 Caption (twice), SI pg 51 Figure S19 Caption (twice), SI pg 52 Figure S20 Caption (twice), SI pg 53 Figure S21 Caption (twice), SI pg 54 Figure S22 Caption (twice)

Italicized instances of variable *t*-test: Original manuscript: Line 335, 664 and Supporting information: SI pg 27 ¶ 1 (twice), SI pg 27 ¶ 3, SI pg 39 ¶ 2, SI pg 39 ¶ 4 (twice), SI pg 40 ¶ 1, SI pg 40 ¶ 3 (twice), SI pg 45 Figure S14 Caption (twice), SI pg 47 Figure S15 Caption, SI pg 48 Figure S16 Caption, SI pg 49 Figure S17 Caption, SI pg 50 Figure S18 Caption, SI pg 51 Figure S19 Caption, SI pg 52 Figure S20 Caption, SI pg 53 Figure S21 Caption, SI pg 54 Figure S22 Caption

Italicized instances of variable *y*-axis: Supporting information: SI pg 27 ¶ 5, SI pg 30 Table S4 (thrice), SI pg 41 Figure S10 Caption (twice), SI pg 42 Figure S11 Caption (twice), SI pg 43 Figure S12 Caption (twice), SI pg 44 Figure S13 Caption (twice), SI pg 47 Figure S15 Caption (twice), SI pg 48 Figure S16 Caption (twice), SI pg 49 Figure S17 Caption (twice), SI pg 50 Figure S18 Caption (twice), SI pg 51 Figure S19 Caption (twice),

SI pg 52 Figure S20 Caption (twice), SI pg 53 Figure S21 Caption (twice), SI pg 54 Figure S22 Caption (twice), SI pg 55 Table S10 (thrice)

Italicized instances of variable *x*-axis: Supporting information: SI pg 27 ¶ 5, SI pg 30 Table S4 (thrice), SI pg 41 Figure S10 Caption (twice), SI pg 42 Figure S11 Caption (twice), SI pg 43 Figure S12 Caption (twice), SI pg 44 Figure S13 Caption (twice), SI pg 47 Figure S15 Caption (twice), SI pg 48 Figure S16 Caption (twice), SI pg 49 Figure S17 Caption (twice), SI pg 50 Figure S18 Caption (twice), SI pg 51 Figure S19 Caption (twice), SI pg 52 Figure S20 Caption (twice), SI pg 53 Figure S21 Caption (twice), SI pg 54 Figure S22 Caption (twice), SI pg 55 Table S10 (thrice)

Italicized instances of sigmoid regression variables: Supporting information: SI pg 38 Table S8

Reviewer 2. Comment 1. How the size of the proteins will effect on the total protein expression in this case?

Response. The reviewer presents an interesting point which is a challenging question presently being addressed by the protein science community: "how does protein length affect the total amount of expressed protein in a cell?". A search of the published literature reveals that it is believed that mRNA abundance and the influence of codon bias are indirectly correlated with gene length [please see: Urrutia AO & Hurst LD, *Genome Res*, 2003, 13, 2260, PMID: 12975314; Lemos *et al*., *Mol Biol Evol*, 2005, 22, 1345, PMID: 15746013]. Regarding the expression of recombinant protein in *E. coli*, it is typical practice to restrict genetic constructs to single domains or favour smaller genes to improve titres of soluble protein [please see: Graslund *et al*., *Nat Methods*, 2008, 5, 135, PMID: 18235434]. We have not amended the content of of our manuscript in response to this question because this is a challenging generalization to directly apply to our genetic circuit and scDBD construct which lies beyond the scope of the manuscript (*i.e.,* to present the development and application of the TReSR algorithm).

Reviewer 2. Comment 2. How much important is sequence length?

Response. The reviewer raises an important consideration regarding the application of TReSR to redesign TR sequences having various lengths. It is important to note that the success of TReSR is grounded in its ability to identify combinations of contiguous codons that sufficiently reduce the complementarity of genes encoding the same amino acid sequence, thereby rendering those genes amenable to synthesis by PCR based methodologies. While it is expected that larger genes should afford more variability in the codon space encoding the same amino acid sequence, it is also expected that larger genes would require the incorporation of an increasing number of primers for synthesis, increasing the probability that off-target annealing of primer pairs may form concatenated or reduced length constructs. Because these considerations impact the applicable scope of the method, we have devoted considerable text to provide guidelines regarding the implementation of TReSR in the section titled "Implementation of the TReSR Protocol" (revised manuscript Lines 406 – 457) and a paragraph in the discussion (Lines 703 – 729).

Reviewer 2. Comment 3. Have the authors tested repressors other than LacI?

Response. Consistent with the first reviewer's second comment, reviewer number two asks whether we have tested alternative repressors to the experimental TR repressor construct produced by duplicating the LacI DNA binding domain. To date we have employed TReSR to design a set of four smaller dimeric LacI DNA binding domain repressor architectures which exert repression via their dimerization mediated by anti-parallel leucine zippers. We have not yet applied TReSR to design and explore repressor constructs outfitted with alternative DNA binding domains. To ensure that this manuscript is easily interpreted, we have presented information relevant to the TReSR code and its implementation along with its application toward the design, construction, and experimental validation of a single experimental TR repressor.

Reviewer 2. Correction. Please supply high quality images for all figures as they become blurred upon zooming.

Response. When we view the high-resolution version of the images that are downloaded through the links provided in the compiled pdf file, we do not seem to experience this problem (i.e. the zoomed versions are not blurry). We have submitted our figure files to the PACE digital diagnostic tool to ensure that they meet PLOS ONE requirements, and confirmed that they are 300 dpi in the maximum size allowed.
